# The *Lancet* Nigeria Commission: investing in health and the future of the nation

**DOI:** 10.1016/S0140-6736(21)02488-0

**Published:** 2022-03-19

**Authors:** Ibrahim Abubakar, Sarah L Dalglish, Blake Angell, Olutobi Sanuade, Seye Abimbola, Aishatu Lawal Adamu, Ifedayo M O Adetifa, Tim Colbourn, Afolabi Olaniyi Ogunlesi, Obinna Onwujekwe, Eme T Owoaje, Iruka N Okeke, Adebowale Adeyemo, Gambo Aliyu, Muktar H Aliyu, Sani Hussaini Aliyu, Emmanuel A Ameh, Belinda Archibong, Alex Ezeh, Muktar A Gadanya, Chikwe Ihekweazu, Vivianne Ihekweazu, Zubairu Iliyasu, Aminatu Kwaku Chiroma, Diana A Mabayoje, Mohammed Nasir Sambo, Stephen Obaro, Adesola Yinka-Ogunleye, Friday Okonofua, Tolu Oni, Olu Onyimadu, Muhammad Ali Pate, Babatunde L Salako, Faisal Shuaib, Fatimah Tsiga-Ahmed, Fatima H Zanna

**Affiliations:** a UCL Institute for Global Health, London, UK; b The George Institute for Global Health, UNSW Sydney, Sydney, Australia; c Department of Medical Social Sciences, Northwestern University Feinberg School of Medicine, Chicago, IL, USA; d School of Public Health, University of Sydney, Sydney, NSW, Australia; e Department of Community Medicine, Bayero University, Nigeria; f Aminu Kano Teaching Hospital Kano, Nigeria; g Department of Infectious Diseases Epidemiology, London School of Hygiene and Tropical Medicine, London, UK; h Department of Epidemiology and Demography, KEMRI-Wellcome Trust Research Programme, Kilifi, Kenya; i Department of Paediatrics and Child Health, College of Medicine, University of Lagos, Lagos, Nigeria; j Vesta Healthcare Partners Nigeria Limited, Ikoyi, Lagos, Nigeria; k Health Policy Research Group, University of Nigeria Enugu Campus, Enugu, Nigeria; l Department of Community Medicine, College of Medicine, University of Ibadan, Nigeria; m Department of Pharmaceutical Microbiology, Faculty of Pharmacy, University of Ibadan, Ibadan, Nigeria; n Center for Research on Genomics and Global Health, National Human Genome Research Institute, National Institutes of Health, Bethesda, USA; o National Agency for the Control of AIDS, Abuja, Nigeria; p Vanderbilt Institute for Global Health, Vanderbilt University Medical Center, Nashville, TN, USA; q Infectious Disease and Microbiology, Addenbrookes Hospital, Cambridge University Hospitals NHS Foundation Trust, Cambridge, UK; r Division of Paediatric Surgery, National Hospital, Abuja, Nigeria; s Department of Economics, Barnard College, Columbia University, New York, NY, USA; t Dornsife School of Public Health, Drexel University, Philadelphia, PA, USA; u Nigeria Centre for Disease Control, Abuja, Nigeria; v Nigeria Health Watch, Abuja, Nigeria; w Hospital for Tropical Diseases, University College London Hospitals NHS Foundation Trust, London, UK; x National Health Insurance Scheme, Abuja, Nigeria; y Department of Pediatric Infectious Diseases, University of Nebraska Medical Center, Omaha, Nebraska, USA; z International Foundation Against Infectious Diseases in Nigeria, Abuja, Nigeria; aa Centre of Excellence in Reproductive Health Innovation, University of Benin, Benin City, Nigeria; ab University of Medical Sciences, Ondo City, Nigeria; ac MRC Epidemiology Unit, University of Cambridge, Cambridge, UK; ad Research Initiative for Cities Health and Equity, School of Public Health and Family Medicine, University of Cape Town, South Africa; ae Nuffield Department of Primary Care Health Sciences, University of Oxford, Oxford, UK; af Health, Nutrition and Population (HNP) Global Practice and Global Financing Facility for Women, Children and Adolescents, World Bank, Washington DC, WA, USA; ag Harvard T Chan School of Public Health, Boston, MA, USA; ah Nigeria Institute for Medical Research, Lagos, Nigeria; ai National Primary Health Care Development Agency, Abuja, Nigeria

## Executive summary

Health is central to the development of any country. Nigeria's gross domestic product is the largest in Africa, but its per capita income of about ₦770 000 (US$2000) is low with a highly inequitable distribution of income, wealth, and therefore, health. It is a picture of poverty amidst plenty. Nigeria is both a wealthy country and a very poor one. About 40% of Nigerians live in poverty, in social conditions that create ill health, and with the ever-present risk of catastrophic expenditures from high out-of-pocket spending for health. Even compared with countries of similar income levels in Africa, Nigeria's population health outcomes are poor, with national statistics masking drastic differences between rich and poor, urban and rural populations, and different regions.

Nigeria also holds great promise. It is Africa's most populous country with 206 million people and immense human talent; it has a diaspora spanning the globe, 374 ethnic groups and languages, and a decentralised federal system of governance as enshrined in its 1999 Constitution. In this Commission, we present a positive outlook that is both possible and necessary for Nigeria to deliver equitable and optimal health outcomes. If the country confronts its toughest challenges—a complex political structure, weak governance, poor accountability, inefficiency, and corruption—it has the potential to vastly improve population health using a multisector, whole-of-government approach.

Major obstacles include ineffective use of available resources, a dearth of robust population-level health and mortality data, insufficient financing for health and health care, sub-optimal deployment of available health funding to purchase health services, and large population inequities. Nigeria's demographic dividend has unguaranteed potential, with a high dependency ratio, a fast-growing population, and slow reduction in child mortality. Effective, quality reproductive, maternal, and child health services including family planning, and female education and empowerment are likely to accelerate demographic transition and yield a demographic dividend.

This Commission was written in the context of the COVID-19 pandemic, which has laid bare the inability of the public health system to confront new pathogens with threats to human health. However, despite a history of weak surveillance and diagnostic infrastructure, the scale up of COVID-19 diagnostics suggests that it is possible to rapidly improve other areas with sufficient local effort and resources.

The *Lancet* Nigeria Commission aims to reposition future health policy in Nigeria to achieve universal health coverage and better health for all. This Commission presents analysis and evidence to support a positive and realistic future for Nigeria. The Commission addresses historically intractable challenges with a new narrative. Nigeria's path to greater prosperity lies through investment in the social determinants of health and the health system.

### Addressing multiple, intersecting disease burdens in a diverse population requires an equal balance between prevention and care

Nigeria is not making use of its most precious resource—its people—by not adequately enacting policies to address preventable health problems. Health is influenced by access to quality health services, but other influencing factors lie outside this sphere. Huge gains in health can and must be made by ensuring adequate sanitation and hygiene, access to clean water, and food security, especially for children, and by addressing environmental threats to health, including air pollution.

Nigeria has a young population, yet, despite spending more on health than many countries in west Africa (mostly from out-of-pocket payment), Nigerians have a lower life expectancy (54 years) than many of their neighbours. Nigeria's lower life expectancy is partially due to having more deaths in children of 5 years and younger than any other country in the world, including more populous India and China and countries experiencing widespread long-term conflict, such as Somalia. Chronic diseases and a high infectious disease burden, and an ever-present risk of epidemics of Lassa fever, meningitis, and cholera, present additional challenges. A rising population and inadequate infrastructure development over the past 30 years have contributed to increasing deaths from trauma through road injuries and conflicts driven by inequitable distribution of resources.

Addressing Nigeria's health challenges requires a whole-of-government and whole-of-society approach to prevent ill health. This means investing in highly cost-effective health-promoting policies and interventions, which have extremely high cost–benefit ratios, and offering clear political benefits for implementation. Interventions are needed to improve child nutrition, reduce indoor and outdoor air pollution, address unmet family planning needs, and improve access to safe drinking water and sanitation.

**Key messages**

• We call for a new social contract centred on health to address Nigeria's need to define the relationship between the citizen and the state. Health is a unique political lever, which to date has been under-utilised as a mechanism to rally populations. Good health can be at the core of the rebirth of a patriotic national identity and sense of belonging. A commitment to a “One Nation, One Health” policy would prioritise the attainment of Universal Health Coverage for the most vulnerable subpopulations, who also bear the highest disease burden.
• We recommend that prevention should be at the heart of health policy given Nigeria's young population. This will require a whole-of-government approach and community engagement. An explicit consideration of equity in the implementation of programmes and provision of social welfare, education and employment opportunities should be paramount.
• We propose an ambitious programme of healthcare reform to deliver a centrally determined, locally delivered health system. The goal of government should be to provide health insurance coverage for 83 million poor Nigerians who cannot afford to pay premiums. Implementation of a reinvigorated National Strategic Health Development Plan (NSHDP III) should be supported by structured and explicit approaches to ensure that Federal, State and Local Governments deliver and are held accountable for non-delivery. NSHDP III should be supported by a ring-fenced budget and have a longer horizon of at least a decade during which common rules should apply to all parts of the system.
• At the same time, the system should encourage innovation. Future health system reform should engage communities to ensure that existing nationally driven schemes have local buy-in and are sustainable. Further, since more than 50% of health services are provided in the private sector, often with poor quality and high costs, reforming the policy and regulatory landscape to unleash the market potential of the private sector is important.
• We outline options for improving health financing and ensuring better accountability and distribution of resources. The rationalised governance schemes we have proposed should improve the efficient use of existing resources devoted to health. Ultimately, the proportion of spending allocated to health needs to be increased. We envision a future of Nigeria's health without foreign aid. This will require substantial increase in domestic investments. Foreign aid (multilateral, bilateral, and philanthropic) has led to fragmentation of the already complex health development landscape, with huge asymmetries in legitimacy between foreign actors and the Nigerian state as well as weak accountability. Defragmenting and decolonizing the Nigerian health landscape requires domesticating health financing.
• We recommend a whole system assessment of the invest-ment needs in Nigeria's health security. The COVID-19 pandemic has exposed the weaknesses of Nigeria's health security system. Nigeria needs better manufacturing capacity for essential health products, medicines and vaccines, the provision of diagnostics, surveillance and preventive public health measures in health facilities and community settings, as well as other preventive and curative measures.
• We call on the Federal Government, working with state governments, to fund and lead the development of standards for the digitisation of health records and better data collection, registration and quality assurance systems. A National Medical Research Council with 2% of the health budget and central government funding to award competitive peer reviewed grants will support high quality evidence and innovation.

### Governance and prioritisation of health are the first places to start

We call for the thoughtful use of existing institutions as an approach to achieve better governance and prioritisation of health. Although corruption has undermined the Nigerian health system, we can harness existing institutions for the benefit of population health. All levels of Government in Nigeria (federal, state, and local), and traditional leadership structures, civil society, the private sector, religious organisations, and communities, influence health.

Efforts towards a balance between centralisation and localisation should focus on common policies, standards, and accountability. Concurrently, there is an equal need for localisation of implementation, meaning actual community and local government ownership of health service delivery. All three levels of Government are crucial, and we provide recommendations for each level. Differences in regional needs and context must also dictate programmes and interventions. What is needed in the northeast, in a context of ongoing insecurity and a crisis of internally displaced persons, is quite different from needs in wealthier, more secure urban centres, or in the face of the different level of insecurity found in oil-producing areas in the Niger Delta.

Prioritisation of health requires additional funds. We have provided a clear investment case on health to convince politicians and governments that improved population health will reap political, demographic, and economic dividends. Our call for a whole-of-government approach to health will allow the delivery of multisectoral policies to address the social determinants of health, prioritise health-care expenditure to major causes of burden of diseases, and substantially increase healthy and productive lifespans.

### Leapfrogging the health system into the 21st century

Nigeria's health system was built in an ad hoc way, layering traditional community health systems with colonial medicine aimed at maximising resource extraction. This origin has resulted in inbuilt inequalities, a dysfunctional focus on curative care, and a detrimental social distance from users and communities. Post-independence policies to redress problems have only been partially implemented.

However, the current health system is sprawling, multifarious, disintegrated, and frequently inaccessible, with very minimal financial risk protection and low financial accessibility of services. Nigerians variously seek care from medical personnel and auxiliaries, community health workers, medicine vendors, marabouts and spiritual healers, traditional birth attendants, and other informal providers. The system relies on a mixture of quasi-tax-funding, fee-for-service, and minimal health insurance coverage.

What kind of health system do Nigerians deserve, and should the country's leaders work towards? The core need of most Nigerians today is for accessible basic health services, and for this to be achieved, improvements in public sector delivery supported by an enhanced complementary private sector, including faith-based organisations, is the way forward.

We lay out a path for Nigeria to move towards a system that, although remaining diverse, better serves the needs of the population. Within this diversity, we believe there is an opportunity for a “one nation and one health” approach, whereby Nigeria guarantees a minimum standard and delivery of health care for all with an emphasis on strengthening public and private (including faith-based and non-profit) systems. Nigeria should also leverage the private sector for certain functions, such as expanding innovation, discovery, and manufacturing capacities to claim a leadership role on the African continent and globally. Government investment in private industry should be mission-driven, supporting innovation and claiming dividends for society from its investments.

Core functions of the health system require immediate attention, in particular, good quality health data. This Commission strongly recommends better recording, storage, and use of data. Paper systems are unworkable. A drive towards digitisation can result in major improvements, for both patient care and devolved health decision-making. Mobile digital technologies should allow a relatively rapid expansion of population health data and linked existing datasets. Human resources in rural and poor regions of the country are worsened by brain drain. We propose prioritising the optimal development and redistribution of health workers at all levels.

### Financing health for all by rationalising contributions from insurance, out-of-pocket payments, donor funding, and taxes

A viable health system requires dedicated, efficient, and equitable health financing mechanisms, complemented by optional health insurance. Countries with systems comparable with Nigeria's, such as Ethiopia and Indonesia, have planned or implemented ambitious programmes to deliver health insurance coverage.

Nigeria's public health system should be supported by a comprehensive health insurance system for all people, funded using through both contributions and taxation, with trials underway in states such as Anambra. Access to health insurance for society's most vulnerable people must be government funded. Considerable political will is needed to bring a greater proportion of the informal sector accessed by most Nigerians under government governance mechanisms.

There is also a need to expand the fiscal space by increasing overall government revenue, which will lead to higher health funding, allowing health and the determinants of health to be addressed. Achieving these financing goals will require an optimistic political economy approach, considering current context, alongside future steps. A starting point could be explicit declaration by governments at all tiers that the achievement of universal health coverage is a priority goal.

Nigeria is a country with so much wealth in terms of human talent and potential, but also beset by challenges, including inadequate provisions for optimal health-care delivery and well-being of its people. For Nigeria to fulfil its potential, the leaders and people alike must embrace the implications of what they know already—that health is wealth.

## Section 1: introduction

Nigeria is at an important crossroad. Nigeria's population is projected to increase from approximately 200 million people in 2019 to an estimated 400 million in 2050, and 733 million people by 2100,^1^ becoming the world's third most populous country after India and China. These estimates assume that the average number of children per mother will decline from 5·1 currently to 3·3 on average by 2050 and 2·2 children on average by 2100. If this projected decline in fertility is to fall short by half a child per mother, Nigeria's population will reach 985 million by 2100. The potential gain from this expansion will only be possible if population growth is managed and supported by equitably distributed prosperity. A rapidly rising population, coupled with the absence of reliable access to high-quality health care, education, and other public services will serve only to increase the potential for unrest, drive large-scale unplanned migration, and consequent regional and even global destabilisation. A large population of uneducated and unemployed youth risks further instability and security challenges.^2^ These demographic and socio-economic challenges are further compounded by climate vulnerability. Nigeria is one of the ten countries most vulnerable to climate change^3^ due to extreme weather, rising sea levels, and increasing land temperature.^4^

Conversely, a healthy and secure Nigerian population living within planetary boundaries could make untold contributions to human progress, now and in the future. Accordingly, integrated efforts to address health inequalities and climate vulnerabilities is a crucial priority for the country.^5^ If the right policies are implemented, Nigeria is poised to become a global superpower.^6^ Nigeria's significance to global health and the health of Africans is self-evident, particularly considering its large and mobile population.^7^ Major health gains in Nigeria should improve health outcomes in Africa by directly improving health security and through the sharing of good practice and policy to neighbouring nations.

But Nigeria faces numerous challenges in confronting both population growth and climate vulnerability ensuring a healthy future for its population. The country did not achieve any of the health-related Millennium Development Goals (MDGs), and progress towards health-related Sustainable Development Goal (SDG) targets has been modest at best.^8^ According to almost all health metrics, Nigeria's health outcomes are dismal with inadequate progress made over the past three decades for the majority of its population. Investment in health is low at 4% of GDP in 2018,^9^ whereas substantial resources continue to be spent fighting insecurity without addressing its root causes, and sustaining a large and complex governance structure, with too little left over for health and education. The macro-fiscal environment is not favourable, with only modest economic growth and a sharp worsening of the economic outlook due to the COVID-19 pandemic.^10^ Conversely, given Nigeria's low starting base, reforms towards achieving universal access to high-quality public health services have the potential to achieve large positive effects on population health outcomes.

Despite its considerable human and material assets, achieving universal health coverage will be challenging. The modest resources allocated to health have been mismanaged by successive governments since independence in 1960. A series of national plans, strategies, and policy documents have only ever been partially implemented, with missed opportunities to apply health as a tool for development. Given the scale of the challenge, there has also arguably been an inadequate focus, with the most recent plan outlining 48 strategic objectives.^11^ Several policy documents allude to “quality, effective, efficient, equitable, accessible, affordable, acceptable and comprehensive health care services” for all Nigerians,^11^ yet these goals are elusive. Nigeria's most recent development plan ended in 2020 with, at best, partial success,^12^ presenting an opportunity to better frame health as a determinant of national achievement in the next plan. There are immense opportunities to alter Nigeria's population health and economic development trajectory, if only they can be seized. Reducing maternal and child mortality and unmet need for family planning are basic first steps to improve families' well-being, with implications for security, resource utilisation, economic growth, and shared prosperity. Reducing the burden of HIV, tuberculosis, malaria, and other communicable diseases will change the epidemiological landscape, allowing greater scope to simultaneously tackle rising non-communicable diseases. Taking bold multisectoral preventive action on the determinants of health can in turn prevent and even reverse the rise of non-communicable diseases. Government action needs to move away from treating disease to creating health. And importantly, such efforts must be integrated with climate action for healthy resilient futures.

The *Lancet* Nigeria Commission aims to reposition future health policy in Nigeria to achieve universal health coverage and better health for all. A detailed critical evaluation of the historical and current challenges facing the health of the country is presented to contextualise recommendations for the future. There is a distinct opportunity to redefine the national social contract using health benefits to the most vulnerable households as a key element of the relationship of citizens to the state. And despite the country's reputation for intractable governance, developments over the past two decades have shown that positive reforms are possible. The initiation of the Basic Health Care Provision Fund scheme and the introduction of state health insurance have provided an important starting point for future reform towards universal health coverage. Improvements in infectious disease surveillance led by the Nigeria Centre for Disease Control (NCDC) have resulted in timely national data reporting on outbreaks of COVID-19^13^ and monkeypox.^14^ Similarly, the completion of the largest ever population-based HIV/AIDS survey by the National Agency for the Control of AIDS (NACA)^15^ within the allocated budget and on time illustrates what is feasible. Success will depend on effective implementation of a coherent set of policies, by translating evidence into action and measuring effects on population health. In this Commission, we aim to present a new path to better health with consequences on development, wealth creation, and strengthening of human capital, notably by proposing comprehensive approaches for improving all components of health care in Nigeria (panel 1).

Panel 1 **Overview of Commission recommendations**

Nigeria has a great opportunity to implement policies that effectively promote population health. Well-intended and seemingly well-designed policies, including most recently the second National Strategic Health Development Plan (2018–22), have often struggled in the past to meet objectives due to poor coordination of a complex multipartner system in the implementation phase and insufficient stakeholder and community engagement, inadequate legal frameworks, perverse incentives, insufficiently robust accountability mechanisms, inadequate adaptation to Nigeria's federal structure, and suboptimal allocation and utilisation of funds. There is now a chance that Nigeria can build on lessons of the past to chart a new course into the future.

Our recommendations build on lessons from within Nigeria's national and state health systems and the experiences of countries that have been more successful in tackling similar challenges. Some of these would require modest resources and be easier to implement, whereas others would require consultation and more fundamental reform. Building on the work of colleagues, we reiterate and reinforce recommendations from previous policy documents, adding evidence-based views on how they can be implemented and by whom.

We call for a new social contract centred on health as a transformational way to define Nigeria's relationship between the citizen and the state. One approach to achieve this goal is committing to a “One Nation, One Health” policy, by offering Universal Health Coverage through greater allocation of ring-fenced resources underpinned by strong accountability systems. This slogan also makes a rhetorical link to the One Health paradigm, which recognises the interconnection between all people, animals, plants, and their shared environment, and the need for a collaborative, multisectoral, and transdisciplinary approach at the local, state, and national levels. Nigeria should also radically revisit its strategy in seven key areas connected to health.

**Recommendation 1**

Political leadership should operationalise previous recommendations to adopt a multisectoral response to health (ie, Health in All Policies) via cabinet-level orders to implement a whole-of-government approach. Each government agency should define goals and indicators aligned to achieving health targets, led by the presidency and with strong funded coordination of the complex multipartner structure by the Health Ministry, National Economic Council, and state governments to:

• Prioritise health investments to address key social determinants of health including adequate sanitation, access to clean air and water, and food security, especially for children
• Consider a standing multisector council on hygiene to coordinate actions of various stakeholders towards prevention
• Enforce existing government policy and regulation on products that are known to be detrimental to health and elevate the risk of non-communicable diseases including sugar-sweetened beverages, ultraprocessed foods, skin lightening cosmetics, and tobacco as outlined in the 2019 National Multisectoral Action Plan for the Prevention and Control of non-communicable diseases, through non-regressive levies and taxation
• Address population growth through improving access to modern contraceptive methods at all health-care levels, female education, and increasing the age of sexual debut
• Adopt an integrated planetary health governance approach, including tackling sources of indoor and outdoor air pollution, and other environmental risk factors, in rural areas and urban centres with a focus on protecting the poor through improved housing and access to clean cooking fuel and enforcing limits on pollution from industrial and transportation sectors, and incentivising transition to renewable energy sources such as solar energy—Kenya provides an example that could be emulated, with the inclusion of health impacts and health in climate adaptation measures into Nationally Determined Contributions, placing health at the centre of policies to reduce emissions in the energy, food, agricultural, and transport sectors for both health and economic returns.^16^
• Integrate health and health service delivery into climate adaptation strategies (examples include integrated surveillance of flood risk and water-related illnesses, climate vulnerability assessments of primary care clinics in communities to limit service delivery interruption in the event of extreme weather events, and urban design that ensures equitable access to green space to reduce land surface temperatures and heat-related illnesses)
• Ensure a multisectoral response that is implemented by Functional Health units, which should be opened in all ministries, departments, and agencies at the federal and state levels where they do not exist, which would ensure that all ministers have a planetary health portfolio with the explicit responsibility of assessing the human health and ecological impact of any decisions, strategies, and policies;^17^ using the performance of this portfolio as an indicator against which all sectors are assessed to encourage and support health creation that is cognisant of climate realities
• Explicitly require equity assessments in the implementation of programmes and provision of social welfare, education, and employment opportunities by federal and state governments
• Systematise a delivery approach across the spectrum—performance management and accountability systems, building on successful application of Emergency Operations Centres as delivery units.

**Recommendation 2**

Federal government, with full engagement of the National Assembly, state and local governments, civil society organisations, the private sector, community groups, development partners, and technical oversight and funded coordination of implementation by the Ministry of Health, should lead a comprehensive reform of the health sector, led by the presidency, to inform the next National Strategic Health Development Plan (NSHDP III) premised on a collectively determined but locally delivered health service, building on policy reforms over the past two decades

• Unify national health delivery standards, improve supply chains, and incentivise manufacturing through national legislation, with funding from the federal level in consultation with state governments
• Build the capacity of local government health officials to deliver basic health services and products based on minimum national standards through state government legislation to prioritise health and support local government implementation
• Define responsibility for governance, purchasing and provision in the health system with oversight and policy formulation led by the Federal Ministry of Health at the national level and State Ministries of Health at the state level
• Maintain high-quality state-government services and enable private sector-run health-care services that are evaluated using federally-led performance management systems for monitoring, evaluation, and quality assurance, using best practices for incentives and penalties linked to targets
• Ensure that auditable public financial management and accountability mechanisms for commissioning and purchasing are developed to improve transparency, efficiency, and equity, and eliminate corruption in the deployment and use of resources
• Build on the improvements in national and state level surveillance and diagnostics achieved through the response to COVID-19 and allocate specific resources to ensure sustainability
• Unlock the potential of health-care markets across the value chains

**Recommendation 3**

The federal government should lead efforts to improve health financing (ie, revenue mobilisation, pooling and management of funds, and purchase of services), aligning the investment case with political incentives, levers of accountability, and the rhetorical appeal of “health for wealth” among the Nigerian population. To achieve these improvements the government should:

• Establish legally ring-fenced predetermined health budgets outside of the electoral cycle, which occurs every 4 years to ensure sustainable funding and strategic planning building on the Basic Health Care Provision Fund, and using the third National Strategic Health Development Plan to reach the goal of 15% of the annual budget allocated to health
• Establish structural reforms to withdraw inequitable subsidies towards financing health and social services building on lessons from the Presidential Task Force on COVID-19 (eg, 1·5 trillion Naira in petroleum subsidy can free up fiscal space to be redirected towards health)
• Fund health insurance coverage to all Nigerians by paying the estimated 15 000 Naira per capita annual premium for 83 million least wealthy individuals (approximately 40% of the population) with revenue raised through the Basic Healthcare Provision Fund, taxation, and levies, and each state to fund residents through their state health insurance scheme supported by a national mechanism to assure quality; today, it would cost 1·2 trillion Naira or 9% of the current budget to cover individuals who cannot afford to pay current premiums in National and State Health Insurance Schemes
• Improve the efficiency of systems for pooling and purchasing of health finances by establishing national and state purchasing organisations with oversight for allocation of funds, raised through revenues generated from taxation, levies, or donors, and the payer at each level should use strategic health purchasing to provide more health services using available resources
• Increase the national fiscal space for health through more efficient tax collection (company profit tax, and capital gains) and through innovative health financing such as levies on commercial services (eg, mobile phone use, financial transactions, and air travel) to reach the existing goal of reducing the proportion of out-of-pocket expenditure to below 30% by the end of NSHDP III and improve health outcomes
• The federal government should anticipate donor transition and prepare for post-aid status in which technical assistance, knowledge, and learning are more relevant than donor projects, which will require domesticating financing of health, research, and development, to achieve health independence and decolonise the Nigerian health space. Local institutions must be prepared to step up.

**Recommendation 4**

Federal and state governments should leverage public–private partnerships based on accountability, mutual trust, information sharing, and joint planning to overhaul Nigeria's dilapidated hospital infrastructure and support manufacturing by:

• Creating an enabling environment for functional health markets while protecting the poor, supported by sound policies, regulations, and access to long term capital
• Using private sector capital to modernise and expand the capacity of hospitals with appropriate accountability mechanisms
• Investing in a coherent state supported and private sector-led approaches to increase local production of vaccines, medicines, and other health products and services

**Recommendation 5**

Federal and state governments should collaborate to address deficiencies and imbalances in the health workforce by engaging in dedicated planning to train and retain adequate numbers of staff at all levels.

• State Ministries of Health should, based on national standards, engage in regular workforce planning reviews to determine the number and type of staff needed at regular time horizons and establish incentive systems to allocate health workers appropriately and specifically increasing incentives to work at primary health care level
• State governments should work with the main health worker regulatory bodies to ease the process of licensure and tracking of members at state level
• The federal government, through the Ministry of Education, should consider expansion of quality medical and allied health professional training to boost human resources and talent
• Federal and state governments should jointly develop and implement strategies to retain staff through career development support, appropriate remuneration and other measures to discourage brain drain between rural and urban areas and internationally

**Recommendation 6**

Federal and state governments should actively manage the demand for health and health services by engaging communities, with a focus on areas such as vaccine hesitancy and the quality and acceptability of government-provided maternity services

• Using adapted guidance from the federal level, local governments should conduct regular consultations to gain a full understanding of community health needs and desires and co-create delivery systems that respond to these needs while ensuring minimum national standards
• State and local governments should establish state-level health forums that meet every 6 months to address local issues with membership drawn from Ward Development Committees across the state
• The federal government should create a national health forum as a deliberative platform for bringing together a wide and inclusive range of stakeholders to discuss complex health challenges, and to provide meaningful and substantive input to NSHDP III
• The federal government should strengthen the voice of citizens using technological and mobile platforms to amplify voices of citizens on needed reforms and accountability

**Recommendation 7**

Define and urgently implement enhanced research and data systems to support planning, monitoring, and accountability at all levels

• The federal government should create a Nigeria Medical Research Council, with permanent federal funding, to strengthen and coordinate health and health-care research; the establishment of the council should be informed by a thorough review of existing research to know where the gaps are. A competitive funding programme targeting investigators at universities, hospitals, and research institutions and complementing other extramural funding systems such as the TETFund should identify research areas based on Nigeria's burden of disease, with priority given to conditions affecting the poorest and most vulnerable
• The federal government, through the Ministry of Health and National Bureau of Statistics, should set national standards for the digitisation of health records, building on existing systems such as District Health Information System version 2, the Surveillance Outbreak Response Management and Analysis System, and the National Health Logistics Information System to improve preventive and curative care, support decision making, and guide system management at all levels. Local and state governments should maintain ownership of local digital infrastructure, using federal funds. Data assurance mechanisms based on a non-blame culture and regular audit cycles improving the quality and timeliness of information, combined with rapid feedback and local use of all collected data, will show value. Federal and state governments should co-fund these data systems, including the cost of internet access for all health workers and access to appropriate technology. The evidence we have reviewed in this Commission suggests digitisation is good value for money
• Federal laws should link access to services and entitlements with registration of births and deaths, and systems to show the value of such data for the economy and to achieve the engagement of civil society. Federal, state and local governments should review existing legislation and develop an action-oriented implementation plan to improve vital registration systems in close collaboration with local stakeholders and institutions such as religious and traditional leaders

**Post Commission phase**

This Commission aims to inspire the next phase of Nigeria's health journey, using evidence-based recommendations to influence the programme of work of the Nigerian Government and its development partners. In tandem with this Commission, we have designed a programme of strategic engagement with key influencers in and out of government, at federal and state levels, to ensure wide dissemination and uptake of key messages among the broader community of policy actors, including the National Assembly. For specific policy makers, we will disseminate the evidence generated through policy briefs and policy roundtables. We will also use the report to generate further discussions using targeted convenings, innovative science-arts approaches, media outreach including via high profile opinion pieces and social media-based delivery of tailored messages for key audiences, including civil society and development partners.

We also hope and expect that co-production of the evidence presented here with members of the target audience, the Nigerian health policy community, will facilitate appropriate and prompt dissemination of our recommendations. We also acknowledge that positive attitudinal change by both the leaders and citizens is key in achieving optimal health outcomes and prosperity in the country. Several senior leaders in the health sector and beyond are contributing to the public engagement strategy to ensure the Commission reaches the right actors. It is intended that through liaison with the two major political parties and by influencing the planned Health Reform Committee of the current government, this Commission will directly set the pace for changes over the next decade. We will continue as a group of experts to work with civil society groups, the Nigerian Government and the legislature to advocate for the changes recommended in this Commission and summarise progress towards implementing the recommendations in future reports and on the *Lancet* Nigeria Commission website. By setting out the challenges, synthesising the evidence, and outlining bold recommendations for action, this Commission presents an opportunity for Nigeria to achieve optimal health outcomes and prosperity ensuring that “health is wealth”.

Our Commissioners combine expertise in the diverse disciplines required to shape national health policy, including public health and epidemiology, political science, history, economics, public policy, sociology, demography, law, anthropology, and health systems. We ensured representation with respect to gender and local origin, included a range of political and health policy views among experts based within and outside Nigeria, and consulted with a diverse group of policy stakeholders to provide insight into the challenges of delivering health and health care in Nigeria. From the outset, we set a 10-year timeframe for our analyses, looking beyond the lifespan of the current Nigerian Government, to ensure relevance to current and future administrations in Nigeria. The core values that underpin this Commission are fairness, equity, pragmatism, and evidence-driven approaches.

The Commission focuses on generation and synthesis of evidence to inform policy and programme implementation, with a view to building a strengthened health system that meets the needs of all Nigerians. First, we review Nigerian history to understand current structures and systems by rooting them in pre-colonial, colonial, and modern-day trends and events. Second, we analyse the country's disease burden, the major causes of morbidity and mortality based on the best available data and models, and projected future trends where possible. Third, we analyse Nigerian health systems and policy, and intersectoral governance and policies that influence health beyond health care, and articulate key challenges and suggested systems-level leverage points. Fourth, we combine health economic analyses with the work on disease burden to generate evidence on the most cost-effective combination of interventions to achieve health goals and summarise approaches to improve health financing. Our concluding section brings together these analyses in the form of specific recommendations and an agenda for action. Finally, we use case studies throughout the Commission to illustrate the lessons, gaps, and opportunities for action. Well-functioning health systems generally prevent maternal, neonatal, and child deaths, and thus we have presented one case study per section of the Commission on this subject.

## Section 2: evolution of a health system skewed away from population needs

### Pre-colonial community health systems provided broad access to holistic care

Organised systems of health-care delivery and disease control have long been present in the territory now known as Nigeria. In the centuries preceding colonial rule, this region was governed by the Hausa States and Kanem-Bornu Kingdoms in the north and the Oyo and Benin Kingdoms in the south. In the southeast, the Igbos used an alternative, more decentralised, governance system, as did numerous other ethnic groups (over 350 ethnic groups inhabit the country today).^18^ Each sovereign area operated its own form of traditional medical care. The *dibia* of the Igbo peoples, *wombai* of the Hausa, and the *adahunse* of the Yoruba peoples were widely trusted to deliver traditional medical care.^19^ Although belief systems and social structures, including health care, varied across these societies, the practice of divination, incantations, exorcism, and other spiritual practices to provide care was commonplace in pre-colonial Nigeria.^18^ Diagnosis and treatment were based on notions of complete therapy and cure, accounting for the individual patient's cultural, social, and physical environment. In particular, diagnosis included sociocultural analysis of the patient's situation, and therapy was sometimes an avenue to cement fragmented relationships between individuals and offended spirits.^20^, ^21^ As many still do today, traditional medical practitioners used leaves, roots, tree bark, animal parts, and minerals from the soil in preparing remedies to heal or prevent unpleasant health events in the lives of their clients.^19^

Health systems were structured so that every community had a full-time healer and nearly every extended family had a part-time healer who could treat common minor ailments.^20^ More serious health problems were referred to a qualified medicine man or woman, as indeed the agricultural surpluses of pre-colonial economic systems allowed for the creation of specialist trades.^20^ Although some healers possessed an integrated body of knowledge of the causes and treatments of diverse illnesses, others (eg, medicine men, diviners, midwives, magicians, bonesetters, and barber-surgeons) concentrated on specific biopathological and social aspects of health. In each community or village, it was not uncommon to find specialists who attended to pregnancy and birth, child health, general welfare, bewitchment, diarrhoeal diseases, and complicated cases such as arthritis.^20^ Consequently, all members of society had access to some basic health services from a healer.^19^, ^22^, ^23^ In modern terms, the health-care delivery could be said to follow a community-based approach, rendering it accessible to much of the population,^24^ with referral systems to specialised healers. Patients with chronic illnesses or incapacitated individuals (eg, people with severe mental illness and leprosy) stayed in special treatment rooms in the practitioner's compound until they were better, whereas those with acute or non-incapacitating illnesses (eg, childbirth or delivery complications, accidents, and bewitchment) were usually treated in their own homes.

However, pre-colonial Nigeria was not a classless society and so access to some health services was unequal. Although treatment and care were sometimes paid for in kind,^19^ those with greater wealth, power, and prestige had more access to the most expensive forms of medicines available, such as for bewitchment.^25^ During the period of state formation that preceded colonialism, population inequities grew substantially. Currency devaluations (in currencies including cowrie shells and copper manillas) and the increasingly virulent trade in enslaved people resulted in societies that were more militarised, stratified, and predatory, sowing the seeds of mistrust of government authorities that is still a factor in state–society relations today.^26^ Yet during this era, the role of the traditional healer was not (fully) commodified; in all ethnic groups, healers had a common role of providing care to all individuals in their communities.

### Colonial health care services laid the foundation for today's inequitable health system

The introduction of Western medicine to Nigeria's pre-colonial societies began with the first incursions of western European traders in the early 15th century and was linked to primarily commercial and extractive ends. During the transatlantic slave trade beginning in the 16th century, doctors were brought to deliver health-care services to slave traders and later to guarantee traders' investments by assessing enslaved people's fitness for travel.^27^ Endemic diseases such as malaria largely impeded European incursions into the interior of the continent. However the use of quinine as prophylaxis and therapy for malaria beginning in the mid-19th century was a major boon for the imperial agenda,^27^ making it easier for Europeans to stay longer, venture further inland, and engage local chiefs and kings in treaties of commerce and so-called friendship, ultimately allowing effective colonisation.

Early colonial authorities established health facilities in cities and towns near the Atlantic coast in Lagos and Calabar, and in Lokoja, the capital of the British Northern Nigeria protectorate, for the use of European merchants, military men, colonial officials, and, much later, Africans employed in mining and construction.^27^ As colonialism gained momentum from the 1860s onwards, mission hospitals also began to appear. Like colonial government hospitals, these were concentrated in Lagos (table 1). Mission hospitals were rarely funded by the colonial government, yet they served the political interest of promoting colonial rule, as preferential treatment was given to Nigerians associated with the Christian mission.^29^ With health services concentrated in urban areas, there was little to no provision for people in rural areas who were less economically valuable to the imperialists. The colonial state-organised Rural Health Units were stifled by funding shortages.^30^ Indigenous healers were still the main providers of health services to most of the population.

**Table 1:** Regional distribution of Nigerian hospitals (1895–1960)^21^ alongside estimated population (1963)

	**North**	**East**	**West**	**Lagos**	**Total**
Government	39	26	24	12	101
Mission	31	16	1	70	118
Unknown*	14	11	5	1	31
Total (%)	84 (34%)	53 (21%)	30 (12%)	83 (33%)	250 (100%)
Population – Total (%)†	29·8 million (54%)	12·3 million (22%)	12·8 million (23%)	675 000 (1%)	55·6 million (100%)

* Leprosarium, nursing homes, and others. Could include some government-owned and mission-owned facilities.

† Data are from the Institute of Current World Affairs^28^

Colonial medical services established the basis for Nigeria's medical and nursing schools, and introduced primary health care (PHC) and hospital care grounded in allopathic medicine. However, the extractive colonial agenda shaped Nigeria's nascent allopathic health system in other ways. The colonial state showed a particular concern for maternal and child health as reproduction promoted population growth and therefore, the expansion of British imperial interests.^31^ Somewhat paradoxically, British health services otherwise maintained a curative bias, with much less emphasis placed on preventive activities such as immunisation, health education, and environmental sanitation. Environmental health campaigns were generally aimed at protecting the European population; for example, Governor of Lagos' massive antimalaria campaign that was initiated around 1900 drained swampy areas and sprayed insecticide to prevent mosquito breeding. Boots, nets, and quinine were distributed only to government officials and their families. Even in the late colonial era, campaigns like the Rockefeller Foundation-funded Yellow Fever Initiative in West Africa, were motivated more by global biosecurity concerns stemming from the rampant spread of yellow fever in countries including the USA, rather than by concern for Africans' health.^32^

The human resource demands of the pre-colonial health system were initially met primarily by European doctors and medical staff. Under the sponsorship of the Church Missionary Society, James Africanus Beale-Horton and William Broughton Davis were the first Nigerian doctors trained in Scotland in 1858, although neither practiced in Nigeria.^33^ The first Nigerian doctor to practice within the country was Nathaniel King who qualified in 1874.^33^ It was not until 1930 that the Yaba Medical Training College in Lagos began training assistant medical officers, and it was not until 1952 that the first teaching hospital in Nigeria, the University College Hospital Ibadan, was established, finally allowing for domestic training of medical and nursing personnel. After the World War 1 and World War 2, many Nigerian physicians became members of the Nationalist Movements demanding better conditions and equality from the colonial government.^34^, ^35^ One response of the colonial government to nationalist agitations was to extend modern health services to all Nigerians, one of several factors leading to the issuance of the 10-year National Development Plan (1946–1956), which projected the building of new hospitals, rural health centres, and nursing training schools. It was during this timeframe that a Ministry of Health was established and health services from all stakeholders, such as the missionaries, colonial government, and trading companies, became centralised. However, the plan continued the prior emphasis on curative services, and there was no budgeted funding for sanitation, health education, or other preventive health-care services. Most new health facilities were in the southern region of Nigeria, mainly in urban areas, as opposed to the rural areas where they were most needed.^20^

### Independent Nigeria's recurring crises and governance challenges hinder efforts to improve population health

Nigeria's independence in 1960 ushered in new hopes to realign state and society and re-orient public spending and governance towards the good of the population. For example, in the 1960s, following the Ashby Commission report,^36^ second-generation medical schools were established in Zaria in the north of Nigeria, Lagos and Ilé-Ifé̀ in the west, and Enugu in the east. Unfortunately, recurring economic crises and ongoing political instability, with a series of military coups in 1966, 1975–76, 1983, 1985, 1993 and persisting until 1999, created a challenging environment for sustained reform. Since the return to democracy in 1999, the political situation has arguably stabilised, albeit with ongoing popular agitation rooted in grievances about the allocation of political and economic benefits. Insecurity is still a major problem in many parts of the country, as are fragile and incomplete democratisation and fiscal weakness. Taken together, these trends have complicated durable progress towards improving population health.^27^

The development of the PHC system in the 1980s and the 1990s under the leadership of Professor Olikoye Ransome-Kuti is a notable exception. Prof Ransome-Kuti, as the health minister, helped develop the first National Health Policy in 1988, and led the introduction of the PHC model in 52 pilot Local Government Areas (LGAs), with the primary focus of promoting preventive medicine at the community level. Among other successes, child immunisation coverage reached over 80% by 1990, meeting the Universal Child Immunisation target.^37^ To ensure the continued progress of PHC service delivery, the National Primary Health Care Development Agency (NPHCDA) was established in 1992. However, the 1993 military coup d'état hastened the collapse of the PHC system and brought an end to the giant strides recorded under the leadership of Ransome-Kuti from 1985 to 1992, and other successes from that period, for example in immunisation coverage, have also not been sustained to the present day. Although PHC is a focus of health reforms—for example with the 2011 Primary Health Care Under One Roof policy, which integrates PHC service delivery under one authority—implementation by states and local governments has been slow and fragmentary.

Key informants familiar with the development of the Nigerian health system in the post-independence period offered varying explanations, many of which appear linked to underlying political issues such as citizens' inability to hold leaders to account (panel 2). Although the colonial inheritance of a generally weak, unequal, curative-oriented system offered a poor start to independent Nigeria, there has arguably been a failure to re-establish a social contract, including an underlying ethos and expectation of the government's duty to provide health-creating conditions, including a functioning basic health system.

Panel 2 **Key informants' views of constraints to the development of the Nigerian health system in the modern era**

We interviewed key informants with direct personal knowledge of the development of the Nigerian health-care system in the modern post-colonial era. Respondents included professors of medicine, former ministers of health, and traditional rulers. They identified key determinants in the development of the national health system:

• Political volatility and constant shifts in political structures meant that positive health reforms were not sustained and consolidated into durable health systems improvement
• Development plans were ineffectively implemented and deployed due to delayed execution, neglect of community stakeholders, non-involvement or limited consultation with medical experts, inadequacy of resource commitment, and neglect of integral policies and institutional structures to enable continuity
• Infrastructural projects were prioritised instead of fundamental development projects designed to effectively tackle Nigeria's persistent health system deficiencies
• Political leaders perceived that investing in the development of efficient health structures would not yield immediate returns, both economically and in terms of goodwill and attribution of success from the served population
• Inadequate funding resulted in degradations of infrastructure and human resources, including the so-called brain drain of qualified personnel
• Constitutional provisions for health are vague, resulting in unclear distribution of responsibilities across governance levels
• Primary health care reform attempts were undermined by a failure to engage with communities to raise awareness, clarify individual responsibilities, or solicit inputs in the reform processes
• Attempts to restructure the three tiers of health-care service delivery occurred in complete isolation, leading to the overburdening of whichever tier was most functional at the period in question
• Political attitudes characterised by narrow individualism and widespread corruption have played a major role in perpetuating the current dysfunctional state of the health sector

See appendix for methodology

One key challenge has been Nigeria's complex, opaque, and poorly specified governance arrangements, which obscure constitutional responsibility and accountability. Since 1979, Nigeria's federal presidential system has divided responsibilities between federal, state, and LGAs, and although the 1999 constitution asserts that “The State shall direct its policy towards ensuring that there are adequate medical and health facilities for all persons” ^38^ (a provision it transparently does not meet), little further detail is enshrined about how this entitlement is meant to be delivered. Since the 2014 National Health Act, the tertiary level of care is nominally the responsibility of the federal government, states manage secondary healthcare, and the primary level, including PHC centres, are managed by LGAs. In reality, the separation is non-existent as states still have their own tertiary care facilities, whereas for primary care, the federal government provides a regulatory advisory function, alongside centralised provision of some services (such as immunisation) and finances infrastructural improvements through the NPHCDA. The poor delineation of responsibilities among these levels has resulted in a complex and contested distribution of resources, a referral system widely agreed to be defective, and an unclear responsibility structure that frequently results in neglect at all three levels. This division of responsibilities partly explains why primary care is generally weak in Nigeria as responsibility for this critical level of care has been devolved to the weakest level of government (ie, LGAs) while control of primary care resources is driven by the state governors.

**Figure 1:**
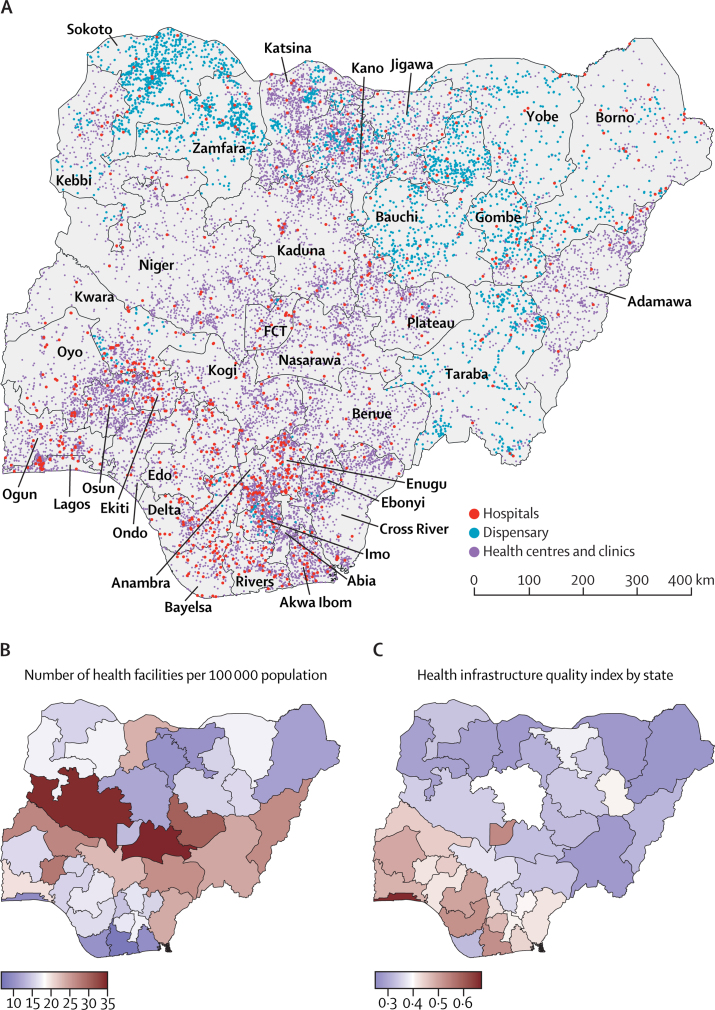
(A) Distribution of public hospitals, health centres/clinics and dispensaries in Nigeria (2019), (B) Number of health facilities per 100 000 population and (C) Health infrastructure quality index by state, 2012

The division between federal, state, and local obligations also risks entrenching historical inequities between geographical regions, with areas that were formerly highly centralised and autonomous during colonial rule (eg, in the north of Nigeria) resisting federal autocratic regimes, which has led to retaliation through under-investment in federal services.^39^ These trends can help explain some of the greater concentration of hospitals (managed at tertiary level) and other formal health structures in the south as compared with the north (figure 1), despite the proportionally larger population in the north. Subsequent investment by the state governments and the private sector further ensured that the density of hospitals and health centres in the south of Nigeria improved and diverged further post-independence. Furthermore, although the rural population constitutes about 50% of residents, it is served by fewer health facilities.^40^, ^41^

Further compounding these issues, population health has not been highly prioritised in national and state budgets throughout Nigeria's modern history. It is difficult to escape the conclusion that the political will to deliver “health for all”, including universal health coverage, has been grossly inadequate, due to in part the population's limited ability to effectively demand improved health services. Since the 1970s, financial gains from oil revenues have been a funding source for health, albeit one that political leaders have repeatedly failed to harness. Political turnover has not been an impetus for change; for example, the dire state of the health system was cited as one of the reasons for the 1985 coup overthrowing General Muhammadu Buhari,^42^ however his successor, General Ibrahim Babangida, allocated only 2·7% of the national budget to the health sector.^35^ Following the collapse of petroleum prices thereafter, Nigeria was subjected to the well-documented ravages of the Structural Adjustment Programme, during which both allocation to the health sector and per capita expenditure on health were reduced.^43^ Out-of-pocket payments have since become the most common mechanism of financing health care for individuals and households,^44^ creating a cost barrier and decreasing the use of health-care services and adherence to medications. Prospective patients are thus driven to use traditional medicine, which is easily accessible and relatively affordable.

Government health expenditures have risen somewhat under the Fourth Republic, however, Nigeria's total government spending as a share of overall health spending was at 4·6% in 2017, lower than the African average of 7·2% and the world average of 10·3%.^45^ In contrast, out-of-pocket expenditure is extremely high, at 77% of total health spending in Nigeria, compared with 37% for the African average, and a much lower 18% for the world average. Compounding Nigeria's health inequities are low in investment in water and sanitation infrastructure compared with other low-income and middle-income countries (LMICs),^46^ as well as generally low government spending across sectors.

Overall, Nigeria's model of health-care financing since the First Republic has gradually transformed into one focused on the generation of revenue for hospital management through the charging of user fees. Public health centres have been pseudo-commercialised as they are restructured to generate funds to work efficiently and independently. In the public and organised private sectors, neoliberal reforms have led health-care provision to be more market-oriented, even though 60% of the Nigerian population are estimated to have minimal disposable income.^47^ As a result of underfunding, the capacity and quality of government health facilities and health services dwindled due to the persistent unavailability of drugs and equipment, resulting in increasing reliance on home treatment, medicine sellers, traditional medical systems, and faith healing by the Nigerian populace.^48^ The accumulated results of this history can be traced throughout the health system, with a case study of maternal health services providing an example of the resulting challenges and opportunities (panel 3).

Panel 3 **The effect of historical trends on maternal health services**

The delivery of maternal health-care services relies on the entire health system, and systems-level issues influence access to and uptake of services, quality of care, and health outcomes.^49^ Therefore, the historical construction of the Nigerian health system can be examined through the lens of maternal mortality, and the diagnosis is dire. Nigeria's maternal mortality ratio (814 per 100 000 livebirths in 2019) is among the world's highest, and the country accounts for 20% of the world's maternal deaths.^50^ Large inequities in access to perinatal health services (including antenatal care, delivery, and post-natal care), are found along the political, social, and economic fault lines characterising the allopathic health system since its origins in the early colonial period. There are marked disparities between geopolitical zones,^51^ with women in northern Nigeria less likely to deliver in a health facility than those in southern Nigeria,^52^ and also between urban and rural areas,^51^, ^53^ with rural residents twice as likely as their urban counterparts to drop out between antenatal care and delivery. Distance to the health facility is a common barrier to accessing antenatal care and facility delivery,^53^, ^54^ compounded by poor road access and unavailability of transport late at night or during the day, especially in rural areas.^55^ The poorly functioning referral system, with unclear repartition of responsibilities between the three levels of governance leads to late presentation and consequent adverse maternal outcomes in tertiary facilities.^56^

The ability of women to seek maternal health services is substantially moderated by the cost of care, an unsurprising finding given that out-of-pocket payments have become the main source of financing of basic healthcare. High cost is a major factor hindering both the use of antenatal care services and the decision or ability to give birth in a facility.^57^, ^58^ Cost as a barrier is most pronounced in rural and semi-urban areas, and the challenge is even greater when women are referred to a higher level of care. The most common reasons for discontinuation of treatment include the cost of services, drugs, and laboratory expenses, and scarcity of transportation to the hospital (which is also often an issue of cost).^59^ These problems are not irremediable; pilot federal government-led initiatives that include financial incentives in the form of subsidies for antenatal care services or free maternal and child health services increase antenatal care utilisation.^54^

Problems of access for ordinary Nigerians, including pregnant women, are measured not only in miles or naira, but also in the social and cultural distance between traditional, holistic care systems and formal medicine, which has only widened since the pre-colonial period. In the formal health system, the poor attitude and behaviour of health facility staff influence antenatal care, formal delivery, and postnatal care service use.^57^ A systematic review^60^ found a broad range of disrespectful and abusive behaviours towards parturient Nigerian women, ranging from non-dignified care to physical abuse, which sowed distrust and undermined service utilisation. Aversion to such abuse is only one component of the psycho-social barriers to access. In the context of Nigeria's pluralist health system, there is often a strong preference for accessing traditional maternal care, which differentially effects maternal morbidity and mortality, and contributes to health inequities. Rather than in formal facilities, women frequently deliver in herbal or traditional maternity homes, on church premises, or at home due to cultural beliefs, avoidance of the patriarchal (medical) system, affordability and ease of payment, convenience, strong interpersonal relationships with healers, and fear of medical procedures, such as blood collection for investigations and pelvic examinations during delivery.^54^, ^55^ There is a strong sense of trust in traditional birth attendants, especially in rural communities, as they are perceived to be more compassionate than formal health workers and provide options such as home delivery, presenting an opportunity to skill up community-based providers who can support normal deliveries and refer pregnant women needing further care.

## Section 3: an evolving burden of disease challenges a system focused on curative care

### Burden of disease

#### Demographic context

With an estimated population of 206 million, of which almost 44% is aged under 15 years, Nigeria is both the most populous nation in Africa and one of the youngest.^61^ Almost 111 million Nigerians are of working age (25–64 years) compared with 95 million of non-working age, and the size of the workforce is projected to grow substantially. Although the UN Population Fund refers to such a situation in which the share of the working-age population is larger than the non-working-age group as a demographic dividend with the potential to drive economic growth into the future,^62^ no country has tapped into these benefits while faced with unchecked population growth. East Asian countries (eg, Singapore, Indonesia and South Korea) used demographic changes to achieve economic development by incorporating new workers and driving up incomes, productivity, and development indicators.^63^ However, these countries also simultaneously tackled population growth by lowering fertility rates; between the 1950s and 2010, the total fertility rate in east Asia declined from 5·8 to 2·3.^64^

Unfortunately, Nigeria still ranks very low on the World Bank's Human Capital Index 2020, as one of only 24 countries out of 174 globally with a score below 0·4.^65^ Indeed Nigeria's score of 0·36 out of 1 means a Nigerian child born today will only be “36 percent as productive when she grows up as she could be if she enjoyed complete education and full health”.^66^ Therefore, investments need to be made now to enable the demographic dividend and to avoid population growth outstripping economic growth and pushing more people into poverty.

The country can harness its human resources by ensuring population growth is managed well and a demographic dividend is realised. By investing in reducing unmet need for family planning, closing the gender gap in education through increasing education of the female population, increased opportunities for women's participation in the labour market, and reducing child mortality, Nigeria's fertility rate can decrease towards replacement levels (2·1 children per woman) so that the large proportion of children and youths today at the bottom of the population pyramid become the engine of the economy (Onwujekwe O, unpublished). Such a bulge in the middle of the population structure if realised would mean a high workforce to dependents ratio and can drive growth. This growth is conditional on large investments in good education and health now so that the potential workers of tomorrow are skilled and healthy.

**Figure 2:**
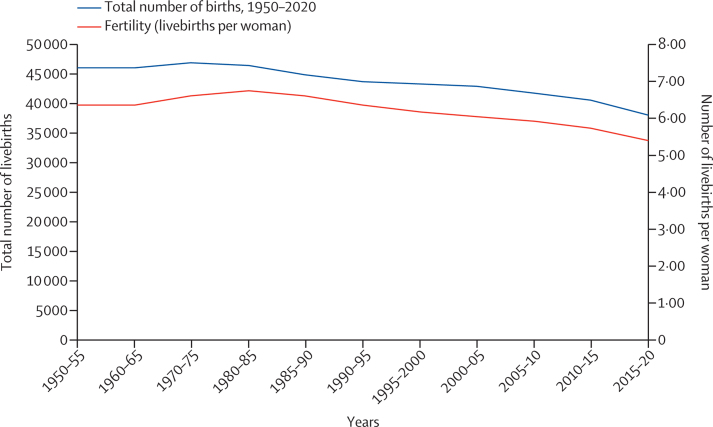
Fertility rate and total births in Nigeria, 1950–2020 Data are from the UN Department of Economic and Social Affairs.^61^

**Figure 3:**
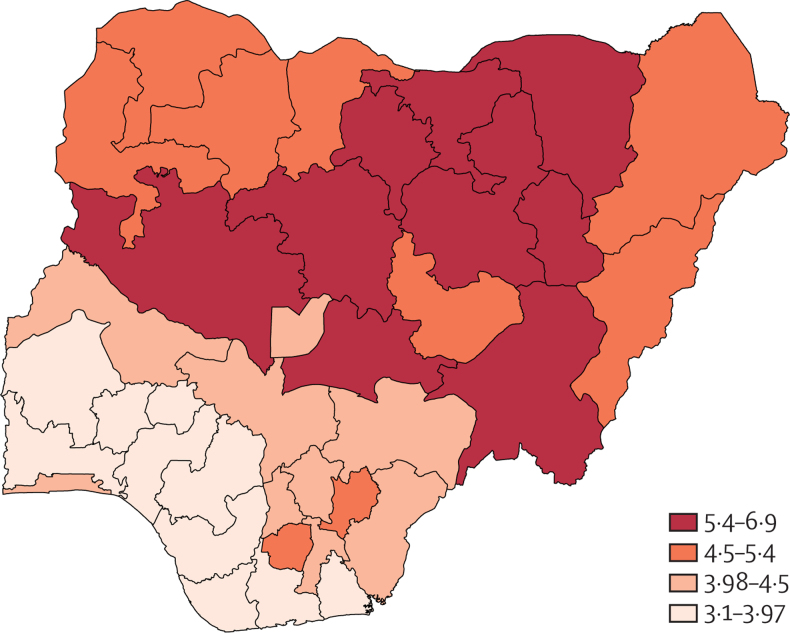
Nigeria Average Household Size by State from the National HIV/AIDS Indicator and Impact Survey, 2018/19—Sample Size

Despite modest decreases in fertility over the past four decades, the fertility rate is high relative to the global average, at around five livebirths per woman (figure 2). The number of births across the country has continued to increase,^67^ leading to population growth of 2·6% a year, a rate that will lead to a doubling of the population within less than 27 years, placing extensive pressure on communities and social services.^61^ This figure masks substantial variations in fertility across the country. Various states in the northern geopolitical zone, rural and poorer households, and specific sociocultural and religious groups report higher fertility rates.^68^ Data from the National HIV/AIDS Indicator and Impact Survey reported an average household size ranging from 3·8 in the South-South Region to 5·9 in the North-East Region (figure 3), and lower in urban areas compared with rural regions.

Faster decreases in fertility driven by family planning and female education, especially in regions and groups with the highest growth rate, will be required for Nigeria to effectively reap its demographic dividend.^64^, ^69^ Ensuring access to family planning and contraception is vital to ensuring gender equality and human rights, reducing unplanned pregnancies and achieving broader improvements in health, education, and economic outcomes.^70^, ^71^ Yet, unmet need for modern contraception in Nigeria is estimated at over 20%, with only slight decreases in the past two decades.^72^ Unmet need among married women is estimated to be lower than among unmarried women but still high.^73^ Meeting the demand for contraception and increased investment in and access to education services is therefore essential, and will require wide-reaching efforts to overcome gender inequities across the population^74^ and taking into account sociocultural challenges that drive high fertility.

**Figure 4:**
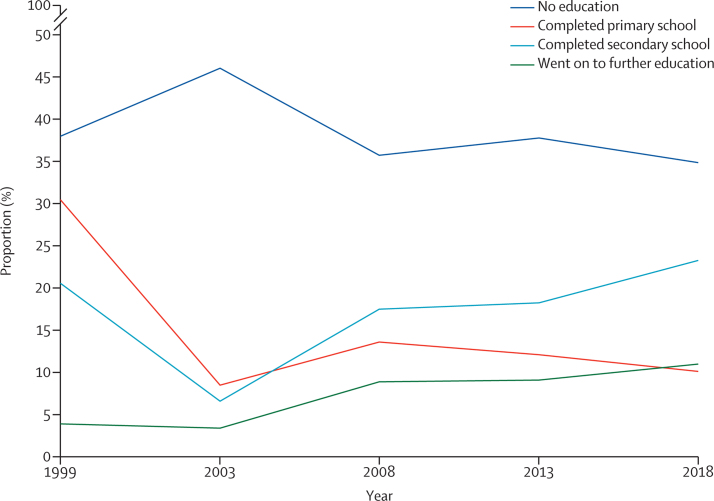
Trends in percent distribution of women aged 15-49 years by highest level of schooling completed, Nigeria DHS 1999–2018 Data are from the National Population Commission (Nigeria]) and ICF 74.

Indeed, the link between the empowerment of women and increased demand for modern contraception has been shown around the world. More broadly, education has been shown to be a key determinant of health seeking behaviour, with the effect particularly pronounced for girls.^75^ Although the proportion of Nigerian women aged 15–49 years with no education has reduced between 1999 and 2018, it is still relatively high, with 34·9% of women having no formal education as of 2018 (figure 4). The percentage of women with secondary education or higher is highest in the south of Nigeria and lowest in the north (appendix p 32), indicating the long distance to travel in improving female education and in addressing the country's regional disparities.^76^

#### Healthy life expectancy, morbidity, and mortality

Nigeria continues to bear an extremely high burden of death, disease, and disability, even compared with other LMICs. The UN estimates life expectancy at birth in Nigeria to be just over 54 years, the fifth lowest in the world (appendix p 33).^61^ The burden of death and disability in Nigeria has historically been dominated by communicable, maternal, and neonatal diseases along with nutritional deficiencies, which continue to be the case in 2019, although non-communicable diseases are having an increasing effect on the population over time.^67^ Much of Nigeria's disease burden is uncertain given the near absence of relevant data; for example, in its latest SCORE assessment, WHO estimates that only 10% of deaths in Nigeria are registered.^77^, ^78^ The paucity of data is strongly indicative that decision making is rarely based on appropriate evidence, an enormous challenge that is nonetheless surmountable provided key hurdles are scaled. Panel 4 summarises the data sources, challenges, and suggests areas for improvement.

Panel 4 **Data systems and quality in Nigeria**

This Commission has relied heavily on the Global Burden of Diseases (GBD) Injuries and Risk Factors study,^79^, ^80^ which provides ongoing estimates of the mortality and morbidity burden attributable to a wide array of conditions and exposure to risk factors in all countries. In addition to the use of GBD results, the Commission undertook bespoke data collection and assessed the quality of existing data to inform future disease burden estimates. Population level data (demographic surveillance sites and census information), national facility-based databases (eg, District Health Information System version 2 [DHIS2]), surveys and surveillance databases eg, Surveillance Outbreak Response Management and Analysis System (SORMAS), Nigeria HIV/AIDS Indicator and Impact Survey, and National Primary Health Care Development Agency immunisation coverage data), and morbidity and mortality records from hospitals across the country were requested.

The process of collating data was not without its challenges, beginning with identifying where the data was situated and requesting permission to access it, due to insufficient institutional memory and frequent leadership changes. Although some organisations were confused as to who the rightful guardian of the data was, others had several custodians with numerous channels to permission, each of whom had to consent for data to be released. Approval processes were therefore complex and slow. Where data existed, as in many health facilities, it was not captured using electronic medical record systems, and was therefore often incomplete and marred with inaccuracies. Furthermore, despite approval from the National Health Research Ethics Committee, each organisation had its own guidelines, which were expected to be fulfilled before data issuance. Reluctance to share data in some institutions was based on concerns about opportunities to publish their own data, cost of extracting data, and apprehensions about privacy and data misuse.

To illustrate the limitations of existing data, we undertook a data quality audit of DHIS2—a key source of input for GBD estimates—in 31 districts across Nigeria selected to achieve geographical representation and data quality spread. We selected districts in the following regions or states: Cross River, South South; Ebonyi, South-East; Oyo, South-West; Kano, North-West; Yobe, North-East; and Nasarawa, North-Central. The results of this audit showed that during the period January to March, 2020, facility-reported data completeness on the DHIS2 platform varied between 58·3% and 71·7%. To illustrate the level of missingness, some public tertiary facilities, responsible for caring for a large proportion of cases out of six facilities audited in each state, did not report any data to the DHIS2 platform. The absence of tertiary hospital data is particularly important for conditions that can only be diagnosed in such centres. In addition to these quality gaps, DHIS2 does not include data from the private sector in most states, where a large proportion of Nigerians access care.

However, some states, such as Lagos, have implemented initiatives to ensure private hospital data are compiled.

Our review of data sources, quality, and analysis support the following measures to improve disease burden estimation and policy making. First, there is a need to create a value proposition for data supported by multiway data communication. As we sourced data for the Commission, we found that institutions often had a shortage of resources and motivation to compile and collate information useful for sub-regional and national planning. Any attempts to sustainably improve data availability, access, and use must feed information back to stakeholders to establish the value proposition. Second, electronic record keeping and digitisation should be implemented. Although there has been some progress in digitising data for health (eg, the recently rolled out SORMAS system during the 2014–15 Ebola virus outbreak for communicable diseases), the vast majority of health records and health-related data are collected on paper and accessible only within health facilities. Nigeria urgently needs to digitise health records at all levels of care. Data-handling and security advances, low cost and portable hardware, new population identifier programs, and National Identification Numbers, combined with a young, digitally-adaptable and under-employed workforce provide favourable conditions for comprehensive health system digitisation. These changes will additionally strengthen supply chains, enable forecasting, and reduce waste. Third, data systems will benefit from strengthening at all stages including collection, collation, and analysis in all sectors. In addition to data on disease epidemiology, health-care staffing, costs, and expenses, Nigeria struggles to collect and manage data around vital registration, demographics, economic activity, educational attainment, and other key development metrics. Population-level data are scarce in Nigeria, census data are rare, and most private sector providers are not part of government data systems. Consequently, there is little information available to provide denominator data for surveys or to parameterise models. Community-led strengthening of vital registration will be essential for future economic and health planning, a step which will require appropriate legislation and enforcement of laws, supported by a digital infrastructure linking data from villages and districts to the National Population Commission and the National Bureau of Statistics. Finally, Nigeria should better support existing Health and Demographic Surveillance System (HDSS) sites and establish additional ones. Worldwide, countries use HDSS sites to collect rich data sets or nest studies to answer pertinent questions that cannot be addressed with national data that have less depth. Nigeria only has two partially functional HDSS sites in operation. Multiple attempts at establishing HDSS sites have collapsed, in part due to insufficient initial funding. A structured, government-funded effort to initiate or re-initiate HDSS sites is needed.

**Figure 5:**
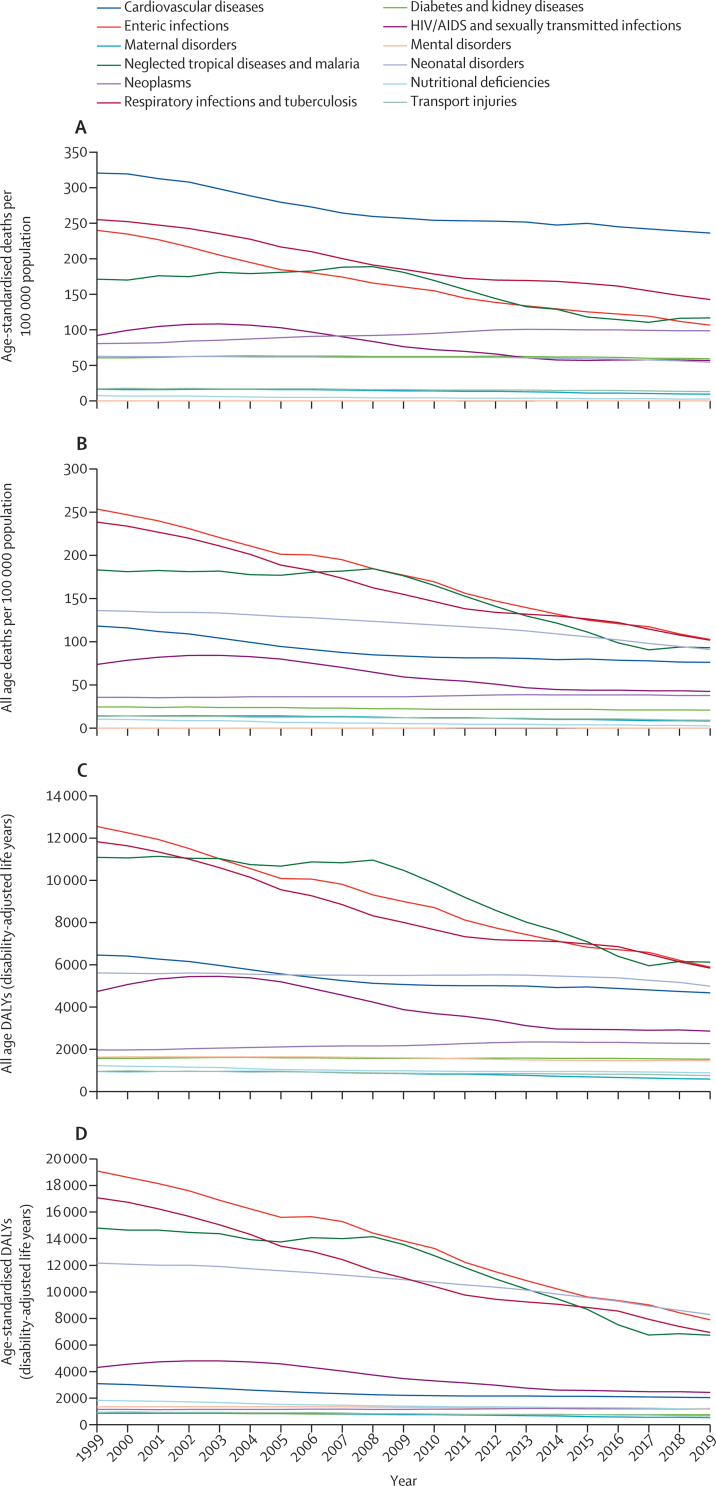
Deaths and disability-adjusted life-years for key conditions in Nigeria, 1998–2019 Data are from the Institute for Health Metrics and Evaluation**.** (A) Age-standardised mortality. (B) All age mortality. (C) Age-standardised disability-adjusted life-years. (D) All age disability-adjusted life-years.

To focus the work of the Commission in understanding and analysing Nigeria's complex burden of disease, an e-Delphi process was conducted in late 2020 with twenty-three commissioners and key Nigerian policy makers to identify the conditions and risk factors most important to address to improve population health in Nigeria (appendix). Eleven conditions and five risk factors were prioritised as particularly important to the Nigerian health system (figure 5). We present GBD data for these prioritised conditions, including communicable diseases and non-communicable diseases, diseases with epidemic potential, and maternal and child health. A comprehensive analysis of the burden of disease in Nigeria compared with other west African countries is presented in full elsewhere, with key results referenced here.^81^

Nigeria has achieved substantial improvements in the rate of morbidity and mortality of historically leading causes of death. The three leading causes of death in 1998 have shown large declines in age-standardised mortality rates; diarrhoeal diseases have reduced by 59% from 227 deaths to 92 deaths per 100 000 population, malaria from 161 to 112 deaths per 100 000 population (–30%), and lower respiratory infections from 148 to 97 deaths per 100 000 population (–34%). Malaria had the highest age-standardised mortality rate in Nigeria in 2019, however, the importance of cardiovascular diseases has grown with ischaemic heart disease (105 deaths per 100 000 population), the second leading contributor to age-specific mortality, and stroke the fifth (91 deaths per 100 000 population), despite decreases in the mortality rates of 18% for ischaemic heart disease and 31% for stroke since 1998. Taken together, cardiovascular diseases were the leading contributor to age-standardised mortality over the comparison period. Infections and neonatal disorders (panel 5) were the largest contributors to age-standardised years of life lost and disability-adjusted life-years across the population in both 1998 and 2019, reflecting their effect on younger population groups (panel 5). This progress over the past 25 years is salutary, but the burden of communicable diseases is untenably high in Nigeria and the rate of progress has slowed considerably in the past decade. Nigeria has not leveraged water, sanitation, and hygiene (WASH) interventions nor deployed technologies such as enteric and respiratory vaccines, which could have driven a further decline.

Panel 5 **Burden of maternal and neonatal diseases is unacceptably high despite some successful programmes**

Nigeria has stated its longstanding commitment to maternal, neonatal, and child health; however, mothers and children continue to bear a major portion of the national burden of ill-health. Neonatal conditions are in the top three causes of years of life lost in Nigeria, showing the constraints of health-care delivery underpinned by a highly vertical and centralised system. Despite the ongoing burden, Nigeria has achieved substantial reductions in neonatal, under-5, and maternal mortality over the past four decades and there are numerous examples of successful programmes to reduce this burden, which could provide lessons for future programmes to build on. Programmes have been able to increase the uptake of skilled maternal health attendants and encourage the use of facility-based maternal and child health services,^82^ reduce mother to child HIV transmission,^83^ prevent strokes in children with sickle-cell disease,^84^, ^85^ and generally improve collaboration and promote uptake of evidence-based interventions.^86^, ^87^, ^88^

Nonetheless, rates are well above the levels required to meet SDG 3·1 and 3·2 to substantially reduce maternal, neonatal, and child mortality. UN estimates of neonatal mortality in 2019 were of 36 per 1000 livebirths and GBD estimates of 49 per 1000 livebirths, both at levels far above the targets. 12 per 1000 livebirths have been set for 2030 under the SDGs in spite of some reductions in neonatal mortality rate.^81^, ^89^ Levels of stillbirth and under-5 mortality per 1000 livebirths are equally high based on UN and WHO estimates of 117 in 201 9^89^ for under-5 mortality and 43 in 201 5^90^ for still births, far above SDG targets. Estimates for maternal mortality range from 91 7 per 1000 livebirths^91^ from the UN and WHO in 2017 to 528 (351–815) from the GBD study in 2019 compared with the SDG target of 140. These rates of child and maternal mortality are among the worst in west Africa, contrasting sharply with Nigeria having among the lowest mortality rates for males over the age of 50 years in the region.^92^ Further analysis of GBD data suggests that the leading cause of maternal mortality in 2019 was maternal haemorrhage (accounting for 5000 deaths) followed by maternal abortion and miscarriage (almost 2600), and late maternal deaths (over 2200 deaths).

The local burden of disease study analysis^92^, ^93^ shows subnational geographical variation with an increasing gradient of deaths from the south to the north of Nigeria. Between 2000 and 2017, most of southern Nigeria had seen considerable improvement in child deaths, with only the north central states and some coastal communities experiencing a high disease burden.

Among infections, diseases with epidemic potential were also prioritised through the e-Delphi process. Nigeria has experienced outbreaks of cholera, meningococcal meningitis, Lassa fever, and monkey pox over the last decade, with variable case fatalities. Nigeria's epidemic detection and response mechanism is becoming more responsive,^94^ with improved laboratory diagnostics, a better trained cadre of field epidemiologists who can be rapidly deployed when needed, and better coordination from the NCDC. WHO's Joint External Evaluation of Nigeria's pandemic preparedness suggested an improvement in the score from 39% in 2017 to 46% in 2019,^94^ leaving much room for continued improvement. Some of these gains have the potential to be leveraged for other diseases of epidemic and pandemic concern including Ebola haemorrhagic fever, and, most recently, COVID-19. The COVID-19 pandemic has however shown how easily this growing capacity can be strained and there is need to continue to develop the response capacity, and resilience within it, because Nigerians are at risk of several epidemic-prone diseases in addition to the threat from future pandemics.

Non-communicable diseases accounted for an age-standardised 567 deaths per 100 000 people in 2019, overtaking group 1 (communicable, maternal, neonatal, and nutritional diseases) in 2015 as the leading contributor to mortality in Nigeria. After the most prominent communicable and neonatal diseases, cardiovascular diseases are the next leading cause of death accounting for over one-third of deaths caused by non-communicable diseases. Neoplasms, responsible for almost 17% of deaths, were the only prioritised disease group that had a higher death rate in 2019 than 1998 (98 per 100 000 in 2019 as opposed to 80 in 1998). Transport injuries accounted for 4% of deaths. Federal Road Safety Corps data obtained for this Commission suggests that at least 10 966 individuals died in 2019, with a further 71 962 injured on Nigerian roads. The Council for Foreign Relations terrorism tracker estimates a cumulative total of 70 000 deaths from violence between 2011 to 2021.^95^

More broadly, there is an urgent need to boost disease testing infrastructure. Within health facilities, Nigeria needs to better deploy diagnostics, including point-of-care tests that can be used at the primary health-care level. In addition to enhancing individual patient care, these tests will provide the precision necessary to generate high-quality health data^96^ and support epidemic preparedness. Presently, only 13·8% of children under 5 years with a fever had blood taken for malaria testing.^76^ Blood cultures, which are needed to diagnose a range of diseases such as typhoid fever, meningococcal and pneumococcal infections among others, are only available at a handful of sentinel laboratories. Available evidence suggests that the burden from other communicable diseases, which can only be reliably confirmed with laboratory testing, such as pneumococcal disease, is likely to be high and is largely unknown.^97^

Infrastructure deficits negatively effect access to life-saving procedures (eg, emergency caesarean sections and supplemental oxygen supplies for infants with pneumonia) at times of critical need, exacerbating the consequences of late referrals. Simultaneous with addressing the root causes of maternal mortality within health facilities is the pressing need to increase access and acceptability of antenatal and maternity care as an estimated 33% of mothers did not receive antenatal care from a skilled provider.^76^

### Health is made within communities and at home: health creation and disease prevention

Since the late 1990s, it has been widely accepted that improved health outcomes observed across the world's population, and particularly in LMICs, resulted predominantly from improvements in socioeconomic and environmental factors including education, income, and progress in overcoming entrenched social inequalities.^75^, ^98^, ^99^, ^100^ Although a well-functioning and resourced health system will be vital to improve the health of Nigerians, much of the disease burden experienced across the country results from factors that lie outside the health system. A multisectoral or Health-in-all-Policies (HiaP) approach to address key risk factors and social determinants of health including nutrition, access to clean water and sanitation, family planning, and healthy environments would be a cost-effective approach to improve population health outcomes and drive sustainable development, while simultaneously relieving pressure on health services. Cities in particular concentrate many of these social and environmental exposures that adversely affect health. Accordingly, without due attention to health equity, the rapid rate of urbanisation occurring in Nigeria coupled with climate vulnerability can further accelerate health inequities.

### Key risk factors driving the burden of ill-health in Nigeria

Through the e-Delphi process, the *Lancet* Nigeria Com-missioners and key Nigerian policy makers prioritised five risk factors (table 2) alongside the deaths and disability-adjusted life-years (DALYs) attributed to each.^67^ Child and maternal malnutrition accounted for the most deaths and DALYs in both 1998 and 2019, with almost 420 000 (26% of total) deaths and over 39 million DALYs (34%) in 2019. Over this 21-year period, there was a reduction in the rate of death and DALYs per 100 000 people for prioritised risk factors, except high plasma glucose and blood pressure, which increased. Most deaths in children aged under 5 years can be attributed to three risk factors: malnutrition (estimated to account for 54% of under-five deaths), unsafe WASH (20%), and air pollution (15%). In the older age groups, metabolic risks are more prevalent.^81^ Action to address the key risk factors for both child health and for the broader population, as well as other social determinants of health (eg, access to education, overcoming gender inequality, and environmental sustainability) will be important to improving the health of Nigerians.

**Table 2:** Deaths and disability-adjusted life-years attributable to key risk factors in Nigeria in 1998 and 2019

	**Deaths (95% CI)**		**Disability-adjusted life-years (95% CI)**	
	1998	2019	1998	2019
Air pollution	223 951 (179 960–283 558)	197 567 (160 424–240 680)	15 252 238 (12 056 035 –19 663 184)	12 643 592 (9 997 069– 15 930 925)
Child and maternal malnutrition	554 763 (480 200–625 969)	419 866 (330 659–537 308)	49 655 623 (43 079 827–5 931 935)	39 037 560 (31 241 661– 49 104 528)
High fasting plasma glucose	36 861 (28 525–47 899)	57 698 (43 014–74 008)	948 140 (755 737–173 865)	1 535 009 (1 183 849– 1 918 815)
High systolic blood pressure	75 204 (56 323–101 955)	114 125 (89 995–140 573)	1 790 920 (1 339 473–2 463 974)	2 877 768 (2 241 093– 3 602 326)
Unsafe water, sanitation, and handwashing	311 528 (224 820–395 596)	212 217 (162 226–271 595)	23 482 301 (17 289 431–29 399 440)	16 042 318 (12 143 327– 20 910 914)

Data are from the Institute for Health Metrics and Evaluation**.**

### Water, sanitation, handwashing, and nutrition

By addressing maternal and child malnutrition, and ensuring access to clean water, facilities for handwashing, and sanitation for all Nigerians, the country could substantially decrease preventable neonatal and child deaths.^81^ Poor access to WASH predispose Nigerians, and in particular vulnerable children, to enteric infections, among the most common causes of under-five mortality. ^81^ Both trachoma and schistosomiasis, which can be controlled by improving WASH, are endemic in the poorest communities of Nigeria; cholera outbreaks in some parts of the country are also attributable to poor access to WASH.^101^, ^102^ Insufficient or non-existent water supply in health facilities is also a major barrier to infection prevention and control, which predisposes for hospital-acquired infections, and are an important risk factor for maternal and neonatal mortality. Infection Prevention and Control gaps are exacerbated by Nigeria's long-standing infrastructural deficits such as poor access to running water, even for health facilities, and chronic, seemingly intractable, electric power shortages. These in turn make it challenging to implement hand hygiene and other requirements of Nigeria's most recent Infection Prevention and Control policy (SITAN studies).^103^

### Environmental and cardiometabolic risk factors

In line with the increased burden of non-communicable diseases on the Nigerian population, a growing number of deaths and DALYs in Nigeria are attributable to cardiometabolic risk factors (appendix p 33).^81^ Although the majority of this burden falls on older Nigerians, evidence suggests that cardiometabolic risk factors are occurring at increasingly earlier ages.^104^, ^105^ As such, the risk factors for cardiometabolic disease need to be addressed at younger ages, not later, as is commonly believed.

As the Nigerian population continues to grow, the relationship between the population and the environment will be increasingly important. Almost 200 000 deaths were estimated to be attributable to air pollution across Nigeria in 2019 (12% of total deaths). Air pollution was included as a key driver of 24% of neonatal deaths and half of all deaths resulting from lower respiratory infections.^67^ Air pollution is also an important cause of the increased burden of non-communicable diseases alongside the metabolic risks identified, with 31% from ischaemic heart diseases, 38% from stroke, 23% from diabetes, and 58% from chronic obstructive pulmonary disease being attributed to air pollution.^67^ Despite these alarming contributions of air pollution to mortality in Nigeria, previous institutional and legislative frameworks have often focused on mitigating pollution from the oil and gas sector, including transport and generators, even though there is substantial ambient air pollution from other sources such as cooking.^106^

The built environment is also a key determinant of population health, in large part because it affects physical activity. Although there is little to no population-level data on physical activity among Nigerians today, about one-quarter of all deaths are due to non-communicable diseases that can be related to low levels of physical activity.^107^ Increasing levels of physical activity across the population will require widespread, equitable access to open space and opportunities for safe physical activity. The key interventions to address the risk factors presented by both air pollution and low rates of physical activity must be undertaken outside the health sector. For example, road transport is the primary source of ambient air pollution, and transport networks are a key determinant of people's everyday physical activity, so working with the transport sector offers the promise of substantial health gains. Panel 6 shows some examples of potential interventions, however, more research is needed to produce a rigorous evidence base of efficacious and cost-effective initiatives that have been subjected to thorough impact and economic evaluation. Considerable inventiveness will also be needed to implement analogous interventions in Nigeria's other cities, which represent more difficult terrain with access to fewer resources than those pilot areas, and to develop appropriate programmes for rural areas. For Nigeria's urban and rural poor, indoor pollution due to cooking is a major risk. State and local governments should support and incentivise efforts to build well ventilated cooking areas or kitchens and open-air cooking. Governments and civil society should also work towards reducing the use of solid fuel (ie, firewood) to more environmentally friendly fuel sources.

Panel 6 **Case study on intersectoral norm-shifting initiatives in Lagos and Abuja**

**Lagos Urban Development Initiative**

The World Bank estimated that Lagos has the highest premature death rate of any West African city due to ambient air pollution leading to approximately 11 200 deaths in 2018.^108^ Over half of these deaths were in children whereas adults had cardiovascular disease, chronic obstructive pulmonary disease, and pulmonary cancer. The estimated loss from air pollution in 2018 was $2·1 billion (2·1% of the state's GDP). The Lagos Urban Development Initiative is a non-governmental organisation (NGO) that advocates for a more inclusive, liveable, and sustainable Lagos. The team runs three major initiatives to increase walkability, bicycle-friendliness, and promote an eco-friendly environment in Lagos:

• Walkability and bikeability in Lagos Island: this project attempts to implement simple and affordable measures at the local government level to make roads safer for school children, in alignment with the Lagos Non-Motorised Transport Policy^109^ initiated in 2018, which aims to create an environment that supports increased accessibility by prioritising the use of walking, cycling, and public transport. The policy seeks to achieve a more equitable allocation of road space by incorporating non-motorised and public transport in the planning, design, management, and budgeting stages of transport projects. The overall aim is to reduce reliance on personal motor vehicles and subsequently environmental pollution, congestion, and other health and safety challenges such as traffic accidents. The programme is undergoing evaluation by the University of Lagos and the Lagos Island Local Government
• Linear Parks Projects: this project aims to reap the rewards of non-motorised transportation and increased parkland within Lagos through the conservation of wetlands and promotion of bikeability and climate-smart agriculture. This dual objective seeks to increase the health and resilience of the city while rehabilitating degraded ecosystems, increasing the number of trees, and constructing infrastructure for non-motorised transport. In 2018, the Lagos Urban Development Initiative carried out a study to explore the feasibility of the project and its potential benefits for government and citizens. The study produced a design that included a 4 km off-road bicycle trail, parklands, and a so-called agri-tainment centre. This project is being carried out in collaboration with multiple government departments, agencies, and the private sector
• Cargo bikes feasibility study: this project is carrying out a feasibility study on the use of cargo bikes for delivery purposes by the commercial and informal sector within some mapped areas in Lagos Island. The focus is mostly to improve delivery of goods to and accessibility in low-income communities, however, by influencing decisions and regulations by relevant authorities in Lagos Island, it also aims to reduce traffic congestion and pollution and make the roads safer. The project is in its second year and is undergoing evaluation. The Lagos Urban Development Initiative aims to replicate this intervention in other parts of Lagos state if successful.

**Ochenuel Mobility in Abuja**

Ochenuel Mobility is an NGO that aims to develop smart and sustainable mobility solutions in Africa that are people-centred and environmentally friendly. The organisation runs two initiatives to improve walkability and cyclability, and reduce air pollution and its effect in Abuja.

**OpenStreets in Abuja**

OpenStreets is a free event that brings together cycling and walking advocates, community groups, residents, and local businesses to temporarily close major urban roads and streets in the Abuja city centre to motor traffic, and open the road up for people walking, cycling, skating, dancing, and playing. OpenStreets initially started a few years ago in Colombia and is held in different cities across the world, including Cape Town in South Africa and Addis Ababa in Ethiopia, in alignment with the SDGs and the New Urban Agenda. In Abuja, the initiative seeks to overcome the city's car-oriented development, which neglects active mobility modes such as walking and cycling, and hinders mobility for persons with disabilities with a view to enabling residents and visitors to explore neighbourhoods in a safe, fun, family-friendly manner. On an OpenStreets day, a portion of a major urban road (2 km –10 km) is closed to motor vehicles. Musical bands provide live music for participants with distribution of educational materials. In addition, blood pressure and blood glucose screenings are carried out on the street by health professionals. Relevant political leaders are invited to increase the profile of the event and build a culture of returning the city back to the people, in collaboration with the government (Ministry of Health) and NGOs, civil society, private entities, and local community members.

The climate crisis poses an increasingly broad and severe set of threats to the health of the population going forward. The spread of vector-borne diseases is predicted to increase as a result of climate change, potentially increasing the effect of malaria (estimated to account for 12% of deaths in 2019) and other diseases already placing a large burden on the Nigerian population.^110^ At the same time, the widespread effects of climate change on various sectors, ranging from food production to natural disasters to communal conflicts, will have profound effects on the health outcomes of the Nigerian population.^111^, ^112^ Desertification in northern Nigeria is already effecting agricultural ecosystems and food security, and rising sea levels are increasing flooding risk in the Niger Delta and major coastal cities such as Lagos.

These vulnerabilities are set against a backdrop of rapid urbanisation and severe environmental degradation, with the fast-growing population placing further pressures on the very environments needed for healthy living. The interlinked climatic and health hazards emerging from, and accelerated by, unsustainable population growth, urbanisation, and climate change result in acute shocks and chronic stressors that can widen health inequalities by increasing the burden of existing health problems, while creating conditions for the emergence of new diseases and a more dangerous environment, with increased flooding, heat islands, and droughts. These factors further affect health through exposure to unhealthy environments, increased demand on health-care systems, increasing socioeconomic vulnerability, and displacement and conflict that compromise the ability of communities to effectively adapt. Measures to mitigate these effects will be crucial to the health and development of the nation. Research partnerships should evaluate the short-term and longer-term effect of initiatives such as these, and support large scale implementation of evidence-informed interventions that address key environmental risk factors to equitably create health in Nigeria.

## Section 4: health system reform—a pathway to universal health coverage

### Achievements and flaws of the current health system

**Figure 6:**
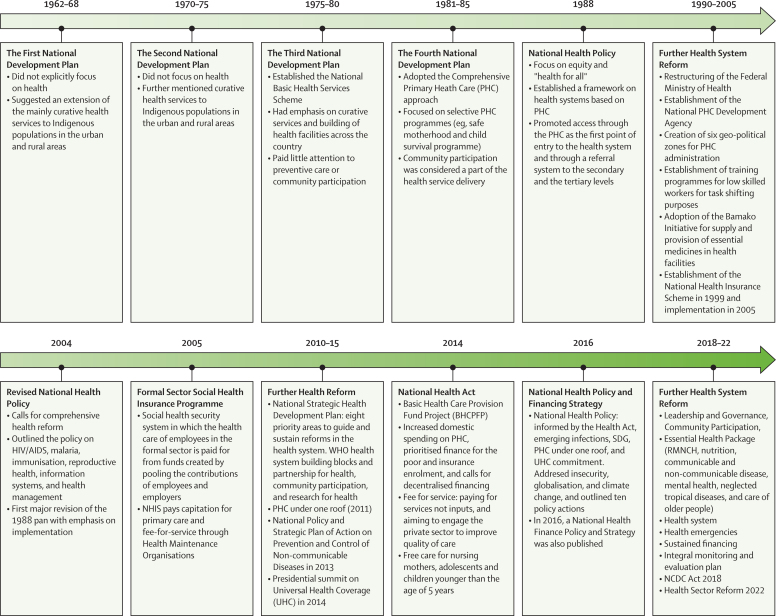
Overview of Nigerian health plans, strategies and policies from independence to present

Indicators of health outcomes and coverage of basic health services in Nigeria show long-standing underperformance. However, the overall trend of indicators such as infant and child mortality since independence in 1960 indicate a slow decline (panel 5). In the past decade, successes against Guinea-worm disease, poliomyelitis, and Ebola virus disease are areas of high performance despite systemic weaknesses.^113^ Numerous health policies and development plans in Nigeria (figure 6) culminated in the National Health Act of 2014, which has the potential to improve Nigeria's health system, by guaranteeing federal funding through the Basic Healthcare Provision Fund and defining state government responsibility for financing and delivery of PHC. Implementation of health policies as intended is a core challenge as illustrated by the incomplete realisation of the Second National Strategic Health Development Plan (NSHDP II),^114^ partly due to governance challenges, with the division of responsibilities between federal, state and local governments. Additionally, the role of development partners in initiating some policies through vertical funding often leads to a lack of ownership by national and sub-national policy makers, and the policy-making process itself not taking formal and informal political considerations into cognisance.

As discussed in Section 2, the three levels of health service delivery in Nigeria (primary, secondary, and tertiary) do not function equally well, and many potential patients bypass the PHC level when not available, trusted, affordable, or of sufficient quality. Individuals who can afford it enter the system at a higher level, and those who cannot afford care at higher levels sometimes resort to seeking informal care from drug shops, pharmacies, and traditional healers, or seek no care at all.^115^ The weakness of the PHC system is linked to the divided allocation of responsibilities between federal, state, and LGA level. The weak state of PHC places a heavy burden on tertiary hospitals, especially where secondary care is also weak or mostly provided by the private sector, such that the bulk of patients are seen at the general outpatient departments of tertiary hospitals staffed by family physicians (ie, general practitioners).^116^

A 2019 survey of all federal government-owned tertiary care hospitals and five state-owned tertiary institutions across Nigeria recorded a national monthly average of about 42 000 visits by patients per facility.^117^ Only 3% of these visits were due to referrals from other facilities, which is consistent with the observed trend of patients bypassing primary care. In addition, less than 45% of these visits led to specialist referrals within the surveyed facilities (table 3) further confirming that these tertiary institutions served as PHC facilities for the patients. These statistics further reflect a heavy burden on general outpatient departments of tertiary hospitals in Nigeria.

**Table 3:** General outpatient department visits and referrals by region—2019 nation-wide assessment of Federal Tertiary Healthcare Institutions (the Hospital Modernisation Pathway questionnaire)

	**Number of patients visiting per day**	Number of patients visiting per month*	**Monthly referrals received by facility**	**Referrals per visit (%)**	**Referrals per 1000 visits**	**Specialist referrals within facility (%)**†
Federal Capital Territory and north central	336	10213	297	2·9%	29	29%
Northeast	402	12 242	1087	8·9**%**	89	21**%**
Northwest	1801	54 826	3996	7·3**%**	73	70**%**
Southeast	3917	119 218	411	0·3**%**	3	53**%**
South	685	20 846	481	2·3**%**	23	40**%**
Southwest	834	25 398	1339	5·3**%**	53	41**%**
National average per facility	1374	41 808	1418	3·4**%**	34	44**%**

* Estimated from values in the preceding column (number of patients visiting per day) based on the assumption that a month is equal to 30·437 days.

† Referrals from General Outpatient Departments to specialists within the same facility.

Previous efforts to provide federal support for PHC have been largely unsustainable—one example is the Midwives Service Scheme. The ambitious nationally implemented government-run Midwives Service Scheme was designed such that each of the three levels of government made monthly contributions towards the salary and support of midwives posted to rural communities to improve the quality of maternal health services. As salaries were inconsistent and insufficient, and in some cases not provided at all (with federal contributions proving the most reliable), the Midwives Service Scheme led to deep dissatisfaction among the midwives employed.^118^

A legislative measure to improve PHC delivery has been to centralise the governance of PHC at the state level, so that state governments rather than local governments take primary responsibility for PHC, in addition to their responsibility for secondary care. Delivering on these responsibilities requires each state government to give due political priority to PHC and increase their health budgets accordingly.

A similar federal initiative to the Midwives Service Scheme, the Free Maternal and Child Health programme, was implemented in 12 states from 2009 to 2015.^119^ This pilot initiative, which provided insurance coverage for mothers and children in selected LGAs in those states, had a similar fate as the Midwives Service Scheme. State governments defaulted on payment of their agreed counterpart funds, which made the federal government terminate the project at the end of the pilot phase. The Free Maternal and Child Health programme was not scaled up within pilot states or to other states. However, it has been shown that the insufficient funds provided for the Basic Health Care Provision Fund could be used to revitalise and scale-up the Free Maternal and Child Health project if mothers and children most in need are targeted for coverage, while sourcing for additional funds to ensure universal coverage of maternal and child health services.^120^

### Rationalisation of policy making at federal, state, and local government levels

To strengthen domains for action and policy in the health system in Nigeria, we propose a reformed set-up of centrally determined but locally delivered systems. Nigeria urgently needs to digitise its health system at all levels. Centrally, there is a need to standardise services, pool and streamline resources, and improve supply chains, manufacturing and data management for products. Concurrently, there is a need to strengthen local production of basic products, allocation decisions, defining basic health services packages to align with local risk factors, and modes of community service delivery sensitive to sociocultural norms.

#### Centralisation

With regards to information systems, evidence and experience indicate the need to nationalise guidelines on completion and use of national surveillance forms, utilisation of data, health information system training and mentoring, data quality assurance processes, and supervision manuals. These guidelines need to be created and made available nationally, with resources provided centrally to support their implementation. Implementation support could be phased out over time and means-tested, based on the level of available financial and technical resources in each state, with appropriate federal assistance. However, during the development of the national guidelines, the specific roles and responsibilities of the three levels of government, and of health facilities in health information system management, should be clearly outlined. Alongside digitising the health information system and centralisation of responsibility, capacity to use information system across Nigeria will require reliable nationwide internet coverage.^121^, ^122^ Stable internet connectivity (which also requires constant electric power), technology hardware, and continuous technical support are core prerequisites for the implementation and utility of electronic information system tools—these requirements are yet to be met in Nigeria.^123^, ^124^, ^125^, ^126^

The vaccine supply chain system (as with other centrally purchased health commodities) receives inadequate and unreliable government funding and is stymied by a complex multilayered governance architecture that depends on several decision-makers at the federal, state, LGA, and health facility levels, and a poorly executed mix of push and pull distribution mechanisms. Re-designing supply chain systems can reduce costs and gaps in cold storage capacity—^127^ for example, by decreasing the number of levels of vaccine in cold storage, increasing the use of local data in procurement of vaccines, and implementing a central (ie, federal or state government) push mechanism and a local (ie, LGA and health facility) pull mechanism.^128^, ^129^ The push mechanism involves pushing vaccines (within a state) directly from a few state stores to PHC facilities equipped with solar refrigerators, thus bypassing LGA cold stores. PHC facilities draw from LGA stores when needed and only when transport can be organised, which occurs often in PHC facilities with a shortage of resources,^130^ resulting in frequent stock outs. In the re-designed system, frontline health workers no longer have to leave their posts to collect vaccines. Reverse logistics, such as waste collection from service points, is a complementary intervention that can be added to the direct delivery programme.^131^ As most vaccines, diagnostics, medicines, and other health care consumables are currently imported, centralising supply chains will also help to streamline and strengthen the logistics and quality assurance of border transactions on importation. A long-term strategy to produce vaccines, diagnostics and other consumables in Nigeria is however necessary, as indicated by the challenge of procuring and distributing personal protective equipment and vaccines in the COVID-19 response.

To increase the demand for and use of health services, the federal government can develop national approaches to shifting normative practices, and improving and standardising quality of care. National guidelines and quality standards could be used to formalise processes and standards of care, for example, beginning with a national pilot of protocols and guidelines for particular services such as maternal health services, which would include antenatal care, delivery, and postnatal care. These protocols and guidelines need to be matched with a national adoption and scale-up strategy, including electronic mobile phone platforms through which their use will be facilitated, and the training, supervision, and monitoring processes to facilitate their use, all made available nationally to all health-care staff, preferably electronically and in a format that would allow for local adaptation.

#### Localisation

PHC workers at the community level are the mainstay of an effective and functional surveillance system. To localise information systems, attention should be paid to strategies to increase the quality (including completeness and timeliness) of data collected, beginning at local community level. One strategy is for health workers to analyse and use the data they collect to make informed decisions in the communities they serve.^131^, ^132^ The goal of data collection should not be solely for transmission to higher levels for analysis and decision making, rather, information should be used at the level at which it is collected. Local use of information can improve data quality as corrections can easily be made at the point of collection in addition to helping health providers monitor their performance. This strategy requires training and ongoing mentoring on data use and interpretation at the local level. Introducing mobile technologies at local level can further improve data quality due to its potential to reduce data entry error and increase speed of reporting, thus enhancing the accountability that local use of data collected at local level can trigger.

Although streamlining and centralisation of supply chain systems is an effective approach to improving the supply side of access to commodities, it is crucial to ensure that the system as a whole is responsive and adaptable to what is happening on the ground—for example, through locally-driven and determined pull processes. Localisation is necessary to optimise centralisation and to avoid creating new barriers to access and coverage. Consideration of contextual factors including local political economy and culture that would influence decisions or guide implementers and policy makers on approaches for implementing direct vaccine delivery at local level is important in retaining a sense of ownership and accountability at local and community level.^130^, ^133^

Careful consideration of context is integral to promoting the uptake and community demand for health services and products (eg, vaccines and contraceptives), especially given that cultural and socioeconomic factors that influence demand and uptake vary greatly across Nigeria. A one-size-fits-all approach will not fare well. Community-based approaches to promotion and delivery of health services would differ due to varying degrees of public security and trust in authorities, and sensitivities or preferences based on religious beliefs and cultural practices.

### Rationalisation of links between public and private sectors

#### Strengthen public sector primarily but leverage private sector for specific tasks

To develop the overall health system landscape in Nigeria, it is important to strengthen the public sector (especially at the PHC level) and expand the capacity of private sector providers to increase their competitiveness in terms of breadth, quality, and cost of services.^134^ Public sector financing should address taxation corruption while increasing health expenditure. Details on economic, and systemic budget reforms needed to strengthen the overall Nigerian health system are presented in Section 5. At the moment, many state governments struggle to mount the required funding to support their health systems. In many instances, when there are limited functional PHC facilities within communities, the private sector fills the service delivery gap in the form of for-profit services (for those who can afford it), non-profit services (by non-governmental organisations [NGOs] and faith-based organisations sometimes aimed at individuals who cannot pay for services) and informal service providers.^135^, ^136^ To improve health care at the PHC level, state governments could strategically secure funding for health systems development with international donors and NGOs in addition to their federal allocation (including specifically for health, guaranteed through the legislative change that centralised PHC governance at the state level).^137^, ^138^ Although foreign funding is not sustainable, with evidence-based planning and foresight, programmes supported by international NGOs and bilateral or multilateral organisations can lead to sustainable health system improvements by establishing health system infrastructure, especially technical and physical infrastructure.^139^

Nigeria has a dynamic private sector that can reposition the health system and improve access and quality of care, as shown during the COVID-19 pandemic. The partnership built between the private sector-led Coalition Against COVID-19 (CACOVID) and the government during the pandemic was a potent force in the country's response to COVID-19, successfully mobilising over 200 donors, including large corporate bodies, to generate 96% of an initial ₦40 billion resource target (Aliyu S, unpublished). CACOVID worked closely with the national and state COVID-19 task force teams to support a wide array of interventions (from case management to economic recovery), representing the first time that the private sector took health system strengthening initiatives to scale, and delivered them in an efficient and coordinated manner, without the encumbrances of public sector bureaucracy, but with appropriate supervision and evidence based decision making from the Presidential Task Force on COVID-19.^140^ CACOVID, through its partners, contributed to the establishment of 39 isolation centres and donated about 400 000 test kits early in the pandemic, supplied food relief materials to an estimated 5% of the poorest Nigerian population, facilitated the vaccine roll out through the provision of logistics and IT support, and provided storage facilities nationwide for food and medical equipment. The logistic systems operated by CACOVID provided broad-based innovative solutions to the challenges of the COVID-19 vaccine and oxygen distribution in the country, thus enabling a more cost-effective and efficient delivery system for goods. CACOVID also helped set up the Nigeria International Travel Portal using their existing IT platforms and skilled staff to expedite the reopening of international airports and economic recovery.

It is important to note, however, the different contexts represented by the pandemic and the provision of health services on an ongoing basis. Evidence and experience suggest that effective contracting of services to the private sector will require a substantial capacity in the government sector to monitor and oversee these providers. Private providers have been found to more frequently deviate from evidence-based practice, have poorer patient outcomes, and be more likely to provide unnecessary testing and treatment in LMICs.^141^ Data on the comparative performance of private and public providers in Nigeria are scarce, although treatment at private providers has been shown to be associated with higher levels of catastrophic health expenditure,^142^ poorer patient satisfaction and prescription of poor-quality medications.^143^ To reinforce public and private sector collaboration in Nigeria, the framework should be contingent on partnerships that are based on mutual trust, sharing of information, joint planning, policy formulation, implementation and evaluation, and joint financing of programmes and activities.^144^

As a starting point to an effective partnership between the public and private sectors, it is important to establish an up-to-date and comprehensive registry of private sector providers in the health system. Such transparency is a central element of good engagement. Encouraging informal providers to at least undertake basic registration is crucial to accurately map the scale and scope of who is doing what in the health system. This registration will be effective if it is simple, cheap, and fast.^145^ Also, it is important to strengthen the mechanism for ongoing dialogue with the private health sector to define common priorities. Furthermore, capacity for strategic financing and contracting of services to the private health sector needs to be properly developed,^146^, ^147^ with the aim of filling access gaps that exist in specific health services across Nigeria, including incentives to provide preventive services, which the private sector often does little of as most of the revenue comes from out-of-pocket payments for curative services.^145^ Finally, it is important to include the considerations for the private health sector in the development of public resources. Reliable and affordable infrastructure are essential for private sector operations even though they are not primarily directed at the private health sector. For health service businesses, access to electricity, water, and sewerage facilities are core technical inputs. Efficient government production of these services has a tangible benefit in terms of creating an enabling environment for private providers.^144^

#### Strategies for human resources and ameliorating brain drain

Deficits in human resources contribute to disparities in health and health-care access. Nigeria has a large health workforce training capacity (and one of the largest health workforce stocks) in Africa, but still has substantial deficits. These deficits are partly due to a correspondingly large emigration of skilled health workers from the country, known as brain drain, which is predominantly due to push factors.^148^ It is not so much that the professionals plan from the beginning (ie, as students) to leave because they are seeking greener pastures, but the conditions (eg, of life and work) in the country typically experienced after graduation that makes them want to leave. Emigration occurs at all stages of professional life, from early to late, so what really matters in the long run is for Nigeria to have a health system that works, so that they can stay. In addition, there are substantial disparities in the supply and distribution of health professionals across states and geopolitical zones. There are no national or sub-national policies guiding the postings and transfers of health workers in Nigeria. Within states and LGAs, deployment is often based on the discretion of administrative officers with multiple influences and competing interests.^149^, ^150^ Such policies are required at different levels of government.

The share of health personnel is relatively low. As of 2010, there are around ten doctors per 100 000 population on average in each state in the country, a figure that is lower than the sub-Saharan African average of 17 doctors per 100 000 population, according to WHO Global Health Workforce estimates.^151^ Community Health Extension Workers are local residents who receive training to provide basic health services to their communities. They generally have less medical training than doctors or nurses and make up the vast majority of healthcare workers at PHC facilities, consisting of up to 57% of staff at health facilities in LGAs, much more than than the proportion of doctors (8%), nurses (14%) and nurse-midwives (21%) at health facilities at LGAs. There are opportunities to up-skill Community Health Extension Workers, creating a professional career path linked to PHC facilities with defined income, roles and responsibilities, and reporting and supervision arrangements, potentially improving access to health services.

To minimise brain drain and maldistribution, it is important to establish local structures for the main regulatory bodies so as to ease the process of licensure, employing, and tracking of health professionals at state level.^152^ Also, state and federal level technical support (eg, digitised registers) should be provided to regulatory bodies to track exit of health workers effectively. These registers need to capture internal and external migration, as well as retiring and deceased health workers, with data periodically updated. Further, human resources for health tracking and data management systems should be set up at state ministries of health and linked to all training institutions and service delivery points, including the private sector, to facilitate human resources for health planning.

Inter-professional rivalries (eg, among pharmacists, doctors, nurses, and laboratory scientists) undermine collaborative care and the ability of the health workforce to meet the care needs of the population.^153^, ^154^ This rivalry, as well as disputes around remuneration, has led to multiple industrial actions like strikes and lawsuits in Nigeria. Such actions can last from a few weeks to several months, disrupting health-care services, worsening health outcomes, and further deteriorating working relationships among health-care professionals.^155^ These challenges require an overarching human resources for health framework developed in a way that secures the relevant buy-in of professional groups.

To improve quality of care, it is important to standardise the training of skilled health workers with national guidelines (eg, in undergraduate training institutions, postgraduate specialist colleges, and for continuing education), in the public and private training and service delivery sectors. There are examples of pilot experiences of using WHO training manuals,^154^ local training manuals,^131^ or adapting international manuals.^131^, ^132^ However, efforts to standardise training and practice will require a national process for developing (and adapting from international examples), accrediting, and disseminating in-service or pre-service training manuals, and incorporating them into nationally approved training curricula.^156^, ^157^, ^158^, ^159^, ^160^

Although the federal government could create national guidelines to improve the quality of care, it is essential that such guidelines are localised within states and LGAs. Improving relations between health workers and the communities they serve requires localised training to the peculiar context of a community; for example, on interpersonal communication or using community education to promote service uptake.^161^ To ensure that health workers can perform their function as educators of the community in matters of health, they themselves first need to learn to communicate effectively and gain community trust.

The cadres of frontline health workers that provide health services vary by primary, secondary, and tertiary providers. In most settings, non-physician caregivers have little capacity to address specific conditions such as non-communicable diseases,^162^ with access to care tightly linked to the country's uneven distribution of doctors. Nurses and community health extension workers contribute substantially in rural areas to tasks successfully shifted from physicians, but more is needed to improve non-physician health-care worker competence for treatment of non-communicable diseases and to leverage them for preventive care. Evidence exists showing that nurses can successfully manage conditions such as hypertension; such task-shifting should be scaled up.^163^ Technological innovation can be used to obviate on-site need for specific scarce skills but requires sustained improvement in power and information technology infrastructure and output. For example, electronic or mobile health (eg, eHealth and mHealth) can be used to provide decision-making support to non-physician health-care workers in decision making at a primary care level and for self-care of non-communicable diseases.^164^ Improving health literacy for self-care or family-oriented care will also contribute by supporting culturally-appropriate interventions.^165^

### Digitisation

#### Digital data for health

Access to media and technology is essential in improving health seeking behaviour. For example, inadequate access and exposure to print or digital mass media (eg, newspapers, magazines, television, radio, and mobile phones) is a consistent predictor of underutilisation of antenatal care services.^53^, ^54^, ^166^ Access to these platforms is associated with awareness of the value and availability of services.^57^, ^167^ Access to mass media tends to be lower in rural settings than in urban settings resulting in inequitable access to evidence-based health information. More efforts are needed to address inequities brought on by information and technological barriers.^168^ Broadcasting infrastructure via radio or digital signal should be prioritised to reach women in remote areas, particularly individuals who are unable to read. Electronic mass media can play a big role in targeting education and efforts to increase awareness.

Furthermore, much work needs to be done to educate Nigerians nationwide on non-communicable diseases. For instance, patients with cancer tend to present with advanced disease, even among people with high literacy. This results in ineffective curative efforts, which contributes to a community perception that cancer is untreatable, and can lead to a vicious cycle of late presentation, high mortality rates, and distrust of the medical establishments, which are then only seen as a last resort.^169^ Integration of basic preventive and curative non-communicable diseases education programmes into print or digital mass media can improve attitudes towards non-communicable disease prevention, diagnosis, and treatment, coupled with training of community health workers and other health workers involved in primary care.

#### Digital data for decision making

Digitising aspects of service delivery can promote access and cost-effectiveness. There are over 190 million active mobile phone lines in Nigeria, or about one per inhabitant, with mobile internet subscriptions of 105 million per month.^170^ The growth and penetration of mobile communications provides millions of people in rural areas access to reliable communication and data transfer technology. The handiness, widespread adoption of, and people's attachment to their mobile phones makes it an attractive platform for delivering health programmes and services. In the hands of trained health workers,^146^ mHealth devices can assist with record keeping, obviating the need for paper-based forms, and reducing wait times with electronic administration systems. The increased use of digitised data will increase its visibility, accountability, and speed of work and communication among facilities and between levels of government.^171^ mHealth devices can also facilitate the quality of service delivery by developing algorithmic clinical guidelines and job aids that could be uploaded and used from mobile phones on which health workers can access user-friendly guidelines and protocols, especially at the PHC level. Although national electronically-enabled guidelines, protocols, and standard of care could be developed centrally, they require space for local adaptation (taking into consideration local disease patterns, human resources for health availability, and task shifting realities), supported by training and mentoring to achieve desired improvements in quality of care.^172^

The benefits of digitising health information systems in a middle-income country such as Nigeria outweigh the investments necessary for its actualisation, and commitment to such a system will eventually lead to increased data quality and usage.^173^, ^174^ In Nigeria, private sector telecommunication companies, backed by international investment are constructing infrastructure for mobile internet access. Regulatory support by the federal government is needed for its improvement in urban areas and expansion into rural and remote parts of the country.

The Federal Ministry of Health (FMoH) has invested in the implementation of a District Health Information System version 2 (DHIS2), which is an open access, cloud-based data management system for data collection, management, and analysis in use by ministries of health in 72 LMICs.^175^ Designed for use in integrated health information systems, it has the potential to increase effectiveness and efficiency of health information management systems.^124^, ^129^ A major barrier to implementing DHIS2 software at the LGA level (ie, LGA health department office) and State Health Management Information System office level is a shortage of or absence of functional computers, internet connectivity, and budget support by states and local governments.^174^, ^176^ Private hospitals and federal public hospitals, which as noted in panel 4 are not currently uploading data to DHIS2, tend to have functional computers and internet connections (even if only in the offices of senior personnel),^122^, ^177^ and equipment which tend to be lacking in state level hospitals and PHC facilities.^177^ Further work to improve the completeness and quality of data in DHIS2 is needed. An essential part of this work will be further transparency and accountability in audits and quality improvement programmes for DHIS2.

Computers might not always be the optimal choice of health information system hardware. First, mobile phones are the most used information and communication technology device in Nigeria,^146^ and most health-care workers know how to use smartphones.^126^ Second, software such as DHIS2 supports data entry via the DHIS2 web portal, mobile Android app, or direct import, meaning that mobile technology hardware such as smartphones can be used for direct data entry by clinicians in facilities, Community Health Extension Workers in communities, or even officers in LGA offices for transmission to a computer in which accountability for it can be assured. Third, data entered through phones is more likely to be cloud-stored compared with data entered on a computer so that the loss or theft of a phone is less likely to result in large amounts of data being lost. Fourth, due to frequent and long-lasting power outages in Nigeria, computers could be impossible to use for real-time data entry, whereas phones and tablets by contrast have long-lasting batteries and require less energy for recharge. Fifth, phones offer more options for connectivity than computers. Finally, for data capture in remote areas, the portability of small mobile devices is an asset particularly when health-delivery materials (eg, vaccines) must also be transported.

Mobile data entry by different people onto the same database speeds up reporting,^126^ and can make the process more convenient for time pressed and overworked health workers. Although it could increase fragmentation in the short term, the degree of fragmentation is substantially less than with paper-based records currently in use. Health-care facilities at all levels, private and public, and State Health Management Information System offices and local governments, should be supported and incentivised to take advantage of collaborative opportunities presented by local and international hardware and software developers and engineers in testing and advancing innovative solutions to their health information and communication technology challenges. To aid progress towards routine use of electronic information systems—whether they are facility based or used for collection of population level data on health and the determinants of health—investment in homegrown health information and communication technology solutions, generation and dissemination of high-quality evidence, and standardised evidence-based guidelines for the adoption and implementation of electronic health information systems are necessary. As the benefits of electronic systems are realised by a growing number of actors in the health sector, demand for health information and communication technology will increase.^121^ Further, digitisation can enable the analysis and use of data by health workers who are also responsible for collecting the data, thus increasing the completeness and accuracy (ie, quality) of data and promoting local accountability,^127^, ^128^, ^129^ and encouraging motivation and commitment. Although digitisation can ensure centralisation of data analysis and decision making, it can also promote its localisation and facilitate completeness, accuracy, and timeliness of data collected, beginning at the community level.

However, we understand that a reformed set-up of centrally determined but locally delivered systems cannot be achieved without the buy-in of ministers of health who will be saddled with the responsibility of midwifing this idea. One key challenge of the Nigerian health system since 1960 is the nature of the qualifications and skillsets of previous health ministers, who are mostly not a good fit to provide the correct leadership and direction of the health sector in Nigeria. For instance, many of the health ministers in Nigeria from 1960 to date have been trained medical doctors, but without broader public or systems training in many cases. Others include a health economist, a professor of parasitology, an educationist, two Nigerian navy admirals, a civil engineer with a law degree, a lawyer who was also a pharmacist, and a reformist or teacher. There is still a latent understanding in Nigeria that medical doctors are more suited for the office of a health minister. As health-care delivery models have evolved, the skillset and portfolio needed in leadership positions to deliver the required improvements in the Nigerian health system need to be updated as well, with political leadership making these selections sensitised accordingly. Nigeria needs health sector leaders who are versed in health policy and administration, health economics, one health, digital health, planetary health, and with vast understanding of what works in the Nigerian context.

### Improved links with communities and traditional institutions

As discussed in Section 2, the history of the Nigerian health system, particularly given its colonial roots, has resulted in a particular estrangement from communities, traditional leadership structures, and religious entities. Yet there is compelling evidence showing that religious organisations play an important role in health promotion and service uptake. Christian and Muslim leaders promote HIV prevention through preaching, counselling, and education sessions,^178^ although religious groups tend to centre their HIV prevention messages on abstinence and faithfulness within marriage, and on punishment and condemnation for people with HIV. There are denominational differences in messaging, and urban churches have more resources for HIV prevention programmes compared with rural churches. In addition, religious leaders across Nigeria have a high level of knowledge about sickle cell disease, engage in premarital counselling on sickle cell disease, and promote genetic testing and counselling among their congregation members.^167^ There is also a tendency for a higher level of contraceptive uptake among women who had exposure to family planning messages from religious leaders compared with individuals without such exposure.^179^

The Global Polio Eradication Initiative in Nigeria was enabled by the positive role that religious leaders play in health promotion and service uptake.^180^ Religious leaders across northern Nigeria, particularly in Muslim communities, mobilised caregivers against social norms that prevent families from vaccinating their children. Muslim and Christian clerics delivered messages during sermons and other religious gatherings to dispel negative attitudes toward vaccinations and other health services. Cooperation between immunisation teams and religious leaders means that vaccines can be received at places of worship. The awareness campaigns championed by the religious leaders have contributed substantially to the polio eradication efforts and reduction in infant and maternal mortality in these states.^137^ Indeed, religious leaders of different faiths are active change agents for shaping norms and informing behaviours about health in Nigeria. As gatekeepers, they substantially influence and shape people's ideas and views about health issues—effects that can be magnified when they form networks with traditional leaders (panel 7). Despite this, both groups are often ignored in efforts geared towards accelerating action in several areas of health.

Panel 7 **Landscape mapping and social network analysis of traditional leaders in six northern Nigerian states**

Traditional and religious institutions in northern Nigeria are among the most trusted and influential institutions in communities across the region. A landscape mapping and social network analysis of the network of traditional community leaders was conducted in six states (Bauchi, Borno, Kaduna, Kano, Sokoto, and Yobe) to describe the characteristics of the group, identify influential persons within the traditional institutions and communities, and describe how their social network influenced health-related decisions at the community level (see appendix for additional details on methodology).

The study analysed actors in separate influence and contact networks, revealing considerable diversity among the six states, in terms of governance of the clusters of traditional leaders in both their influence and contact networks. Each network includes 1000 actors who are mentioned the highest number of times in all interviews. In states such as Bauchi, the study revealed a network of influence with distinct cliques within several local areas and a separate cluster corresponding to actors with state-wide relevance and influence. Influencers in some local areas operated both at local and state levels; in other areas, influencers were only at the local level, and yet in some other areas, only at the state level. In contrast, influence networks in Sokoto did not display distinct local area or state-wide influence structures, likely due to a failure to establish robust governance structures that mix hierarchical and peer relationships. Another type of governance structure was observed in Borno where influence networks within all local areas are well-developed, but a state-wide influence network does not exist, nor are local area-level influence networks connected laterally to one another. This situation means that influencers are confined to a defined local area without access to a state-wide influence network to reinforce or extend their influence.

The composition of influence and contact networks in the six states as a function of actor type showed dominance of traditional leaders in influence networks whereas religious leaders dominated contact networks. The combination of traditional and religious leaders appears to be the mechanism for extending the influence of traditional leaders who have more restricted contact networks. This analysis informed the community engagement strategy developed in the six states to increase demand for routine immunisation and primary health-care services.

## Section 5: investing in the future of Nigeria—health for wealth

### Prosperity, macroeconomics, and health

**Figure 7:**
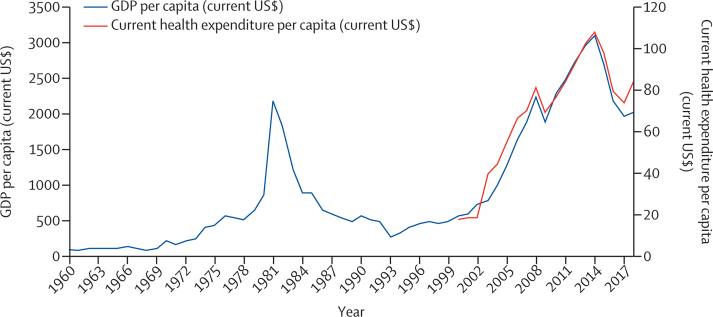
Total Health Expenditure per capita mirrors GDP per capita

The total GDP of Nigeria was estimated at $448 billion in 2019.^181^ Five sectors are the major contributors to Nigeria's GDP with the largest being agriculture contributing about 24·5% to the total nominal GDP in 2020,^182^ followed by trade at 13·9%, manufacturing at 12·8%, information and communication at 11·2%, construction at 7·6%, and mining and quarrying at 7·1%.^182^ The GDP per capita was US$2230 in 2019,^183^ the per capita total health expenditure was $72·7 and the proportion of total health expenditure relative to GDP was 3·6% in 2018.^184^ Moreover, most of this expenditure was private with public spending as a percentage of total health expenditure constituting only 16·3%, whereas private spending as a percentage of total health expenditure was 76·6% and out-of-pocket spending as a percentage of total health expenditure was 74·9%.^184^ External resources as a percentage of total health expenditure was 7·7%.^184^ Total health expenditure as a percentage of GDP has remained between 3·4% and 4·1% since 2006 (figure 7).

Nigeria hosted and signed the 2001 Abuja declaration,^185^ in which African governments committed to allocate at least 15% of government spending to health. The low levels of total health expenditure to GDP ratio and public spending on health, and the heavy burden of disease shows that it is important that overall health expenditure is increased. Without an increase in total health expenditure to GDP ratio, only an increase in GDP will lead to a rise in total health expenditure (from all sources, most currently being out-of-pocket). Nigeria should concurrently increase its GDP, the overall fiscal space, and the proportion of the budget allocated to health as outlined further on in this Commission.

Concerted advocacy and community engagement efforts from committed and powerful national and state-level institutions and civil society organisations could push health (including harm-reduction and pro-health multisectoral policies) and health-care funding up the political agenda, making it a key election issue. In subsequent elections, holding politicians to account at the ballot box for their previous promises to improve health and access to healthy environments, could engender sustained progress. The COVID-19 pandemic and other health security risks could present the opportunity to increase this advocacy through political engagement. The investment case for health spending in Nigeria is clear; health spending provides value for money and can be politically rewarding.

### The investment case

#### Value for money

Spending existing revenues in ways that efficiently and equitably decrease the burden of disease to in turn reduce health-care need is crucial, as is direct investment in multisectoral action to prevent disease (table 4) and better strategic purchasing in the health sector to improve diagnosis and treatment.

**Table 4:** Multisectoral actions for health with high benefit to cost ratios by policy area

	Benefits*	**Costs**†	**Benefit to cost ratio**‡^186^
Universal access to contraception	Around US$60 billion, given that Nigeria has around 20% of global maternal deaths, and around 10% of global newborn deaths and estimated, not counting the reduction in costs in maternal and newborn health services	$25 million in 2022 (of which $22 million is still required)	$120 benefits per $1 spent
Make beneficial ownership information public	An estimated $15·7 billion flows out of Nigeria illicitly each year including theft of billions of dollars of oil revenues;^187^ this money could entirely fund the aggressive scale-up of health coverage to achieve the health SDGs as shown in table 5	Creating and maintaining online beneficial ownership registries and associated incentive or enforcement mechanisms is likely to cost only a few million dollars (eg, a World Bank project in Nigeria is costed at $0·5 million^188^	$49 benefits per $1 spent
Reduce child malnutrition	8·7 million disability-adjusted life-years and 183 000 lives saved annually, 3 million cases of stunted growth among children aged younger than 5 years averted^189^	$837 million annually^189^	$45 benefits per $1 spent
Air pollution (eg, clean transport and industry, and renewable energy rather than fossil fuels)	2·1% GDP loss averted and 11 200 premature deaths (60% in children aged younger than 5 years) averted (according to a study in Lagos state)^190^	Implement and enforce vehicle and industry emissions standards, solar cells, and battery storage	..
Modern cooking fuels	35% reduction in disease incidence, around 1 h time savings per household per day, and forest preservation^191^	Switch to liquified petroleum gas fuel and improved stoves approximately $50 per family per year^191^	$15 benefits per $1 spent
Eliminate open defecation	Around $3·6 billion per year in lost health and time costs due to open defecation and poor sanitation (meaning disease and subsequent time off work?)^192^	Around $150 million per year government investment, supplemented by around $600 million per year by households to construct toilets for all households that have lacked them for 10 years or longer^192^	$6 benefits returned per $1 spent

* Annual health, social, economic, and environmental benefits.

† Annual costs for coverage of population of Nigeria in need.

‡ Global estimates, Nigeria likely to have higher benefit to cost ratio as per the preceding columns.

Rapidly increasing health expenditure, even by as little as $30 per capita per year (around $6 billion per year; table 5) could substantially improve coverage of essential health-care packages resulting in less sickness and death for millions. This increase in health expenditure in turn would improve wellbeing and economic output via increased individual, family, and community activity, and contribute to a rapid improvement in the nation's wealth. Delivering such an improvement in health and wealth would bring political dividends to national and state governments. The promise of this win-win-win situation therefore necessitates overcoming the barriers stopping it from happening.

**Table 5:** Estimated programme and health system strengthening costs 2021–30 extrapolated from the National Health Strategic Development Plan 2 (NSHDP2) (2018–22)

	Moderate scale-up scenario*			**Aggressive scale-up scenario**†		
	NSHDP2 programme costs (billion [₦]) for moderate scenario 2021–30‡	Cost per capita (US$, 2021–30 total)§	Cost per capita, per year ($, from 2021 increasing annually to 2030§	NSHDP2 programme costs (billion [₦]) for aggressive scenario 2021–30‡	Cost per capita ($, 2021–30 total)§	Cost per capita, per year ($, from 2021 increasing annually to 2030§
**Programme area**						
MRH	₦490 billion	$5·48	$0·50–$0·58	₦5403 billion	$57·61	$5·37–$6·07
Child health	₦181 billion	$1·94	$0·19–$0·19	₦2195 billion	$22·97	$0·86–$3·32
Immunisation	₦135 billion	$1·50	$0·11–$0·18	₦2210 billion	$23·02	$0·61–$3·57
Adolescent health	₦231 billion	$2·54	$0·11–$0·37	₦2694 billion	$28·51	$2·15–$3·46
Malaria	₦97 billion	$1·03	$0·10–$0·10	₦758 billion	$8·11	$0·81–$0·81
Tuberculosis	₦24 billion	$0·26	$0·03–$0·03	₦324 billion	$3·45	$0·31–$0·38
HIV/AIDS	₦201 billion	$2·23	$0·16–$0·27	₦2033 billion	$21·74	$2·17–$2·17
Nutrition	₦286 billion	$3·17	$0·21–$0·40	₦3179 billion	$33·72	$2·70–$3·95
WASH	₦59 billion	$0·66	$0·05–$0·08	₦529 billion	$5·63	$0·50–$0·62
Non-Communicable Diseases	₦265 billion	$2·92	$0·16–$0·40	₦2758 billion	$29·26	$2·37–$3·41
Mental health	₦85 billion	$0·94	$0·05–$0·13	₦840 billion	$8·90	$0·68–$1·07
Neglected tropical diseases	₦2 billion	$0·03	$0·003–$0·003	₦57 billion	$0·61	$0·06–$0·06
Health promotion and social determinants of health	₦5 billion	$0·05	$0·005–$0·05	₦69 billion	$0·74	$0·07–$0·07
Emergency hospital services	₦10 billion	$0·11	$0·01–$0·01	₦172 billion	$1·84	$0·18–$0·18
Public health emergencies, and preparedness and response	₦1 billion	$0·01	$0·001–$0·001	₦10 billion	$0·11	$0·01–$0·01
Total	₦2072 billion	$23	$2–$3	₦23233 billion	$246	$19–$29
**Health system strengthening**						
Programme activity costs	₦688 billion	$7·36	$0·74–$0·74	₦999 billion	$10·68	$1·07–$1·07
Human resources	₦6317 billion	$70·54	$6·07–$7·82	₦6430 billion	$68·46	$6·10–$7·45
Infrastructure	₦1074 billion	$11·49	$1·15–$1·15	₦1080 billion	$11·54	$1·15–$1·15
Logistics	₦3623 billion	$40·12	$2·65–$5·15	₦4475 billion	$47·24	$3·11–$5·98
Medicines, commodities, and supplies	₦8320 billion	$92·10	$6·00–$11·89	₦10261 billion	$108·27	$7·00–$13·82
Health financing	₦109 billion	$1·16	$0·12–$0·12	₦149 billion	$1·60	$0·16–$0·16
Health information systems	₦48 billion	$0·52	$0·05–$0·05	₦80 billion	$0·86	$0·09–$0·09
Governance	₦72 billion	$0·77	$0·08–$0·08	₦149 billion	$1·60	$0·16–$0·16
Total	₦20 252 billion	$224	$17–$27	₦23 625 billion	$250	$19–$30

MRH=Maternal and reproductive health. WASH=Water, sanitation, and hygiene. US$=US dollars (with an assumed exchange rate of 395 Naira per dollar).

* Moderate scale-up scenario. NSHDP2—average coverage increase of 17·5% during 2018–22, extrapolated to 2021–30.

† Aggressive scale-up scenario. NSHDP2—average coverage increase of 30% during 2018–22, extrapolated to 2020–30.

‡ 2021–30, a total of 10 years of costs calculated from NSHDP2 costs by extrapolating from 2020, 2021, and 2022 costs from Tables 54 and 55 (moderate scale-up) or Tables 56 and 57 (aggressive scale-up) of the NSHDP2^11^ to get costs for each year from 2021 to 2030, and then summing up those costs. Simple extrapolation of the trend was done by dragging cells in Excel (version 16) (assuming continued constant rate of change). Any negative trend was removed and the projected population increase was also accounted for so that the 2021 per capita cost was repeated for all years to 2030 and used to calculate the total costs for 2021–30 (a flat trend resulting from the removal of a negative trend is apparent in which the per capita costs stay the same throughout 2021–30).

§ Population of Nigeria for years 2021–30 taken from the UN Department for Economic and Social Affairs^1^

Increasing health expenditure as outlined in table 5 only constitutes approximately 2·5 times the current $11·85 (16·3%) public spending of total health expenditure per capita of $72·7 in 2019.^184^ The true costs required, however, are likely to be much higher than these costs extrapolated from the National Health Strategic Development Plan 2 (NSHDP2; 2018–22).

**Figure 8:**
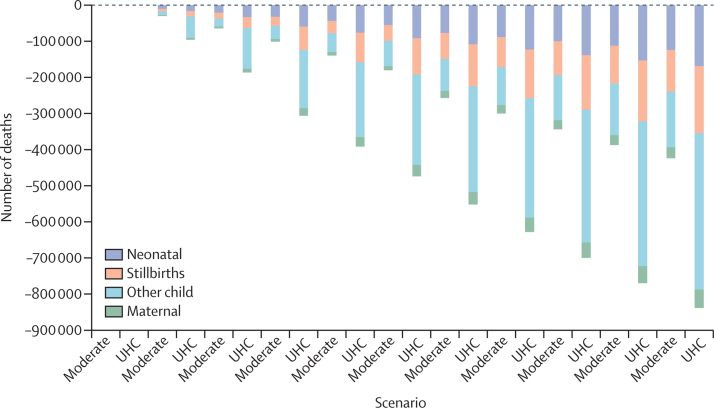
Maternal, neonatal, child deaths, and still births averted relative to baseline scenario UHC=universal health coverage

For example, given experience with the COVID-19 pandemic, far more than $0·01 per capita per year is likely to be needed for adequate provision for the programme area of public health emergencies, preparedness, and response. Similarly, for neglected tropical diseases to be addressed (figure 5), far more than $0·06 per capita per year expenditure is required. Investment in health promotion and social determinants of health also needs increasing from $0·07 per capita per year to several dollars per capita at least, especially considering such funding would reap many times the amount in benefits (table 4). It is also important to note that total health expenditure per capita has been as high as $108 in 2014,^193^ even though health coverage and outcomes are poor, which is likely to be related to the high proportion derived from out-of-pocket expenditure.^194^ Therefore, it would be prudent for the government to at least double annual health expenditure per capita to $168 or perhaps even triple it to $252, while dramatically increasing the proportion that is public expenditure (or pooled funding) and reducing out-of-pocket expenditure, as has been attempted in India and Ethiopia (panel 8, table 6). These increases would entail investments of ₦157 trillion to double annual health expenditure or ₦236 trillion to triple annual health expenditure for the whole of the 2021–30 period, or 10–15% of total GDP over the decade (2020 GDP for Nigeria was estimated at ₦152 trillion^182^). Nigerian Governments must recognise this as an investment in a prosperous nation rather than a cost.^212^ Given the size and importance of this investment, it must also be accompanied by measures that promote efficiency and effectiveness throughout the system, while prioritising interventions that give the most value (ie, health) for money. To illustrate the value of efficient spending on health, panel 9 and figure 8, show how investments in maternal, neonatal and child health can save millions of lives in Nigeria over the next decade. The estimated $10·5 per capita per year is slightly more than that estimated for maternal and reproductive health ($5–6) and child health ($1–3) combined from the NHSDP2 aggressive scale-up scenario (table 5).

Panel 8 **Country case studies of reduction of out-of-pocket expenditure to increase access to health care—India and Ethiopia**

**India**

India, like Nigeria, is a lower-middle income country with historically low health-care expenditure and a heavy reliance on out-of-pocket payments to finance care. Both countries have health systems shaped by the federal structures of their countries, large populations, and fragmented health systems with different levels of government, and complex combinations of public and private sectors that make wide-scale reform difficult. In the past few years, however, India has embarked on a series of reforms to extend health care and financial protection across the population that might offer some important insights as Nigeria pursues universal health coverage. In 2018, it was estimated that 50–60 million Indians were pushed into poverty each year as a result of health-care expenditure,^195^ with the effectiveness of the multitude of insurance schemes at protecting against catastrophic health-care expenditure limited by insufficient resourcing and coverage gaps.^196^, ^197^, ^198^, ^199^, ^200^, ^201^, ^202^ In the face of this challenge, the cabinet of the Indian Government approved the ambitious Ayushman Bharat Pradhan Mantri Jan Arogya Yojana (AB-PMJAY) in March, 2018. The scheme aims to build on existing schemes to provide publicly-funded health insurance cover of up to 500 000 Indian rupees (almost US$7000) per family per year to about 100 million of the most vulnerable Indian families (500 million people, 40% of India's population).^203^, ^204^, ^205^Benefits are India-wide such that a beneficiary can access cashless care across the country; however, state authorities are responsible for the implementation of the programme and they can choose the operating model, either using funding to pay a private insurance provider to cover services, provide services directly, or a mix of the two.^203^ The scheme has been designed to either replace or operate alongside other state-based initiatives.^203^ Expenditure under the programme is shared between the central and state governments in a prespecified ratio depending on legislative arrangements and the relative wealth of the states, with the Indian government covering between 60%–100% of expenditure. A substantially proportion of private providers have been empanelled under the scheme, reflecting the importance of the sector to care patterns in India. Although hospital care is covered under the scheme, AB-PMJAY is built on a substantial increase in primary care investment across India through the establishment of over 50 000 health and wellness centres across the country. The country has also established Health Technology Assessment in India, a new body formed to drive cost-effectiveness through health technology assessments of publicly-financed interventions.^206^ A number of other investments have been made attempting to ensure the system can effectively implement the programme with the establishment of new governance structures and substantial investment in IT systems across the country for example. The programme has also taken on substantial political importance in India, allowing the government to leverage widespread popular support for improved health care.^207^

**Ethiopia**

Ethiopia has made progress in provision of PHC, 208 and out-of-pocket payments expenditure has reduced from 47% in 2011 to 35% in 2018.^209^ Ethiopia's Community-Based Health Insurance scheme has improved access to care, reduced out-of-pocket payments, and also managed to improve quality of care to some extent via increasing health facility revenue that has then been used to reduce drug stockouts and make other improvements that have increased patient satisfaction.^210^ The Ethiopian government has committed to further reducing the effect of high out-of-pocket payments and catastrophic health expenditure by incorporating considerations of financial protection and equity in determining which services to fund. The Essential Health Services Package of Ethiopia 2019^211^ determines which services should be publicly funded and was devised based on seven criteria including burden of disease, cost-effectiveness, and financial risk protection (ie, the potential of interventions to avert catastrophic expenditure or incur financial hardship). Another of the seven criteria is equity, which assessed the effect on specified groups including economically poor individuals and those in remote areas, therefore also contributing to financial protection. All interventions were ranked on their financial risk protection potential and then this score (alongside those for the other criteria) was used in the decision to fund or not fund specific interventions. See Table 6 for a comparison of key health financing indicators between Nigeria, India and Ethiopia.

**Table 6:** Key health-care financing indicators for 2018 for Nigeria, India and Ethiopia

	**Nigeria**	**India**	**Ethiopia**
Gross national income per capita ($US)	2030	2120	800
Health-care expenditure as % of GDP	3·9	3·5	3·3
Health-care expenditure per capita ($US)	83·75	72·84	24·23
Proportion of health-care expenditure out-of-pocket	76·6	62·7	35·47
Out-of-pocket expenditure per person ($US)	64·16	45·64	8·59

Data are from The World Bank DataBank and WHO Global Health Expenditure database.

Panel 9 **Maternal, neonatal, and child mortality in Nigeria—saving millions of lives**

Although there have been improvements over the past 5–10 years, the level of maternal, neonatal, and child deaths are extremely high in Nigeria placing an immense health and economic burden on the population, severely curtailing economic and social development across the country and leaving Nigeria off-track to achieve the related sustainable development goals (panel 5). Although these rates are a national tragedy, experience in other nations suggest that the majority can be overcome with a package of existing, and often low-cost, interventions. To assess the potential effect of health system investment targeting these interventions, we used the UN inter-agency developed Lives Saved Tool (LiST) to dynamically project the health and cost effects between 2021 and 2030 of three scenarios of policy intervention: (1) baseline (no improvements in intervention coverage); (2) moderate increased investment (defined as linear progress to 20% increased coverage of interventions relative to baseline); and, (3) universal coverage (defined as linear progress to 90% coverage of interventions). Further details of the interventions included, the LiST tool, and projection methods are detailed in the appendix.

Under the baseline scenario, the maternal mortality rate worsened slightly by 2030 (a result of demographic changes), and neonatal and under-five mortality rates were largely stable. The results for the other scenarios are striking—figure 8 shows the numbers of deaths averted each year for the moderate and universal coverage scenarios. In total, over 309 000 maternal, 967 000 neonatal, and over 2·61 million child deaths could be averted under the universal health coverage scenario by 2030 relative to baseline. For the moderate scenario, over 160 500 maternal, 664 000 neonatal, and almost 806 000 child deaths could be averted relative to baseline. Importantly, much of this care is nurse, midwife, or community health worker-led (rather than doctor-led), although the scale-up scenarios require large increases in the health workforce particularly for these groups. Given this health impact, the costs required to implement the package are modest and increased investment in the area is likely to be considered very cost-effective under all commonly considered definitions.

An analysis of the estimated additional costs for these 10-year scale-up scenarios suggests that the universal health coverage scenario provides good value for money. Although the universal health coverage scenario is substantially more expensive than the other options, the per capita cost (using the population projections under each scenario) are relatively modest. Under the universal health coverage scenario for example, a total additional expenditure per person equivalent to less than $64 (₦ 26 612) per person will be required for the entire period (2021–30), comprised of steadily rising annual costs that will reach $10·5 (₦ 4366) per person in 2030 on top of baseline expenditure. This amount constitutes approximately a 13% increase on 2018 health spending of $83·75 (₦34 827) per capita. In contrast, the moderate scenario requires a total additional expenditure per person equivalent to just over $12 per (₦ 4990) person over the 2021–30 period, but which a much smaller reduction in maternal, neonatal and child deaths, as shown in figure 8.

Table 7 and table 8 are from an analysis by Ezenwaka and colleagues (Onwujeke O, unpublished), which detail the projected number of health workers and health facilities required to achieve a moderate scale-up of 5% coverage of health care per year for 10 years until 2030. This scale-up is a 63% increase in coverage, and a corresponding 63% increase in all cadres of health workers and health facilities could be needed to deliver this gain. Newly recruited health workers will require training, supportive supervision, and adequate salaries and benefits. New facilities will need to be planned, constructed, and maintained, with a key consideration being facility locations that reflect health needs. In an analysis of health facility data, we show a clear association between the quality of health facility infrastructure and childhood vaccination outcomes at state level (appendix pp 15–16).

**Table 7:** Health workers needed for moderate scale-up scenario by 2030 (5% per year for 10 years, 63% increase over the decade; Onwujeke O, unpublished)

	**Number of staff in 2020**	**Number of staff in 2030**
**Health service providers**		
Community health officers	6227	10 144
Dental technicians	1961	3194
Dental therapists	3384	5512
Doctors	68 415	111 441
Nurses and midwives	12 836	20 908
Medical laboratory scientists	20 001	32 581
Medical laboratory technicians	8533	13 899
Nursing assistants	1560	2542
Occupational therapists	35	57
Optometrists	2784	4535
Pharmacy assistants	107	175
Pharmacy technicians	1923	3134
Physiotherapists	2932	4776
Senior community health extension workers	44 673	72 767
Junior community health extension workers	29 607	48 227
**Health management and support personnel**		
Accountants	3762	6128
Administrative officers	7043	11 473
Catering, cooks, and stewards	1062	1730
Cleaners, labourers, and gardeners	329	537
Clerical officers	7055	11 493
Community health extension workers admins	1979	3223
Community health officer admins	566	922
Data processing officer	712	1161
Dental technician admin	240	391
Director admin	8736	14 230
Environmental health officer	6806	11 087
Health information management officer	1038	1691
Health record officer	4434	7223
Messengers	322	526
Mortuary officer	30	49
Nurse and midwives admin	2977	4850
Nurse and midwives tutor	201	328
Pharmacist admin	569	927
Pharmacy technician admin	253	413
Planning officer	553	902
Plaster technicians	40	66
Procurement officers	403	658
Senior auditors	93	152
Social worker	1013	151
Statistician	396	646
Store officers	1505	2452
Ward attendant	448	730
Watchman and security	751	1223
Works and maintenance	4696	7649

**Table 8:** Health facilities needed for moderate scale-up scenario by 2030 (5% per year for 10 years, 63% increase over the decade; Onwujeke O, unpublished)

	**Number of facilities**		**Average number of beds**		**Average occupancy bed rate (%)**	
	Baseline year (2020)	Target year (2030)	Baseline year (2020)	Target year (2030)	Baseline year (2020)	Target year (2030)
Primary	28 202	45 938	8	14	45·2	73·6
Secondary	1327	2162	91	163	57·3	93·4
Tertiary	104	169	346	621	63·9	95·0
Specialist hospital	35	57	147	264	59·5	97·0
Federal medical centre	24	40	308	553	..	..
Federal health agency	8	13	..	..	..	..
Federal ministry of health	1	2	..	..	..	..
Regulatory agencies	13	22	..	..	..	..
Health training institute	23	38	..	..	..	..

Multisectoral action on the determinants of health will play a key role in any effort to improve health, including those related to diet, water and sanitation, air quality, transport, habitation, and the environment.^213^ Local and state governments should also be empowered to take a place-based approach to the health of the population. This approach would include resource prioritisation for PHC and zoning authority to prioritise healthy environments, thereby, putting the health-in-all policies approach^214^ into action beyond words. One without the other is insufficient and simply results in an unchecked increase in disease burden and health-care needs that cannot be adequately addressed in PHC.

#### Political benefits

Targeted investments in the health sector that will improve financial and physical access to priority public health services and consequently improve health outcomes should markedly bolster the positive image that the citizenry have of the initiating level of government. Examples of past programmes that have paid off politically include the Saving One Million Lives initiative targeting maternal, newborn, and child health interventions, the NPHCDA Primary Healthcare Centre Revitalisation strategy targeted at ensuring there is at least one PHC in every ward across the country to promote access, and the Primary Health Care Under One Roof strategy to integrate management of PHCs for more efficiency, as well as the now defunct Subsidy Reinvestment and Empowerment Programme for Maternal and Child Health (SURE-P MCH) and National Health Insurance-Millennium Development Goals (NHIS-MDG) programmes. The branding of such special programmes is usually effective for galvanising political support and ensuring that the beneficiaries are aware of the sources of the interventions.

### Ensuring accountability and mitigating against corruption

Corruption has been defined as the abuse of entrusted power, such that a person, group, or organisation acquires undue benefits. These benefits might be financial, material, or non-material.^215^, ^216^ Health systems are especially susceptible,^217^, ^218^ often with life-threatening consequences. Yet, corruption in the health sector is often seen as intractable.^215^

Health sector corruption impedes access to health care, therefore, effective solutions are needed to tackle them. Universal health coverage should mean action on determinants of health and health-care provision. The Nigerian Government and commercial and industrial interests therefore need to be held accountable for ensuring Nigeria is a healthy country to live and grow in. Nigeria cannot achieve universal health coverage if the system is corrupt. The most common types of corruption in the Nigerian health system, which has to be eliminated for the country to make reasonable progress in achieving its health goals are absenteeism, procurement-related corruption, under-the-counter payments, health financing-related corruption, and employment-related corruption*.*^218^

Experience from other settings shows that although the health sector plays a crucial role in advocating for this approach, there is need for a cross-sectoral mechanism to facilitate and coordinate measures to address corruption (eg, the office of the governor or mayor or equivalent).^219^, ^220^ There is also a need to explicitly address accountability, value for money, and corruption in the Nigerian health sector. Institutional mechanisms to govern procurement, prevent informal payment, and discourage absenteeism also need to be set-up and managed with high-level oversight and accountability. Informal payments and employment-related corruption were most feasible to tackle. Frontline workers and policy makers agreed that tackling corrupt practices requires both vertical (eg, regulations and penalties) and horizontal approaches (eg, collective efforts of health workers, local government administrators, and community groups in the locality of health facilities and grassroots movements).^218^

To improve accountability, transparency, and efficiency, and reduce corruption, it will be important to strengthen public financial management systems within the health sector at the federal, state, and local government levels. This process will involve governments ensuring that expenditure tracking mechanisms are instituted and routinely applied at all levels of government for both government and donor spending, and the Ministries of Finance promptly transferring funds to institutions within the health sector against approved plans. There should be capacity building of managers at national and subnational levels on budget negotiation and management. Data systems and transparently publishing information will allow citizens to hold public officials to account, especially if revenue is raised primarily through taxation. Finally, given that many of the officials that are responsible for implementing these accountability systems are often directly or indirectly involved in the failings, we draw on the ideas of North^221^ to call for action that goes beyond individuals and focuses on institutions, broadly defined to include formal and informal processes to help in achieving accountability.

### Funding the health system

#### Funding sources and their shortcomings

**Figure 9:**
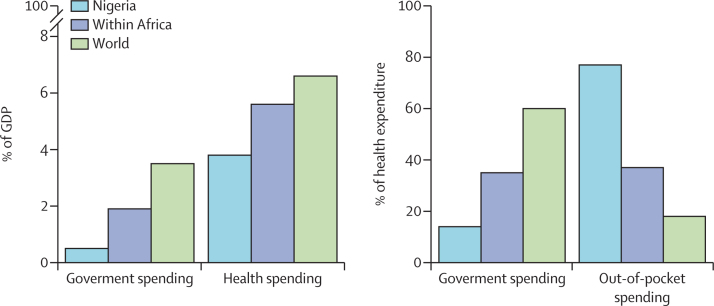
Health spending statistics per region, 2017 Data are from The World Bank DataBank.

Nigeria continues to spend very little on health and health care compared with its peers in the region and around the world (figure 9). As of 2018, the share of government spending assigned to health care was only 5·2%, slightly above the average of 3·4% between 1981 and 2018. The share of government spending has been as low as 0·2% (1992) with the highest share of spending in 2011 at about 7% (appendix p 33). Compared with the rest of Africa, Nigeria's Government spending on health care is only 0·5% of GDP, lower than the African regional average of 2% and the world average of 3·5% as of 2017 (figure 9).

Government spending in total health expenditure across four major categories (government spending, out-of-pocket spending, external development assistance, and prepaid private spending) is both lower and more volatile in Nigeria compared with other countries in the sub-Saharan Africa region.^222^ The share of government spending in total health expenditure was 26% on average between 1995 and 2014, lower than the sub-Saharan Africa average of 37%. Out-of-pocket spending is a much higher share of health expenditure in Nigeria than in sub-Saharan Africa (appendix p 34). There is a strong negative correlation between the share of government spending in total health expenditures and the share of out-of-pocket spending in total health spending in Nigeria at –0·92 (p<0·001), presenting a contrast with the sub-Saharan Africa region where there is no significant correlation between the two. In Nigeria, the share of out-of-pocket spending on health was 67% on average between 1995 and 2014, reaching 70% in 2014, which is much higher than the average of 31% in sub-Saharan Africa over the same period. Nigerians are forced to rely on out-of-pocket expenditure when government spending on health falls, imposing a substantial economic cost in a country where 40% of the population, nearly 83 million people, live below the poverty line of $382 per year according to the 2019 National Bureau of Statistics estimates.^223^

The National Strategic Health Development Plan 2 (2018–2022) set a target of reducing out-of-pocket expenditure to 35% of overall health expenditure, however the latest available data from 2019 show that out-of-pocket expenditure has not reduced and still accounts for over three-quarters (78%) of all health expenditure.^193^ Indeed out-of-pocket expenditure has not been below 70% since 2005. The vast majority of the burden of financing health care therefore is on the people of Nigeria, presenting a serious barrier to care-seeking, entrenching inequality, and inducing catastrophic health expenditure and medically-induced poverty for many Nigerians every year. The World Bank estimates that 50% of Nigerians were at risk of catastrophic health expenditure for surgical care in 2020, with the percentage varying from 38% to 54% between 2007 and 2020.^224^ Studies in Enugu and Anambra states have shown rates of 15%^225^ of actual catastrophic health expenditure in 2008 and 27%^226^ in 2010, with catastrophic spending measured at a threshold of 40% of non-food expenditure.

In the past two decades, external development assistance has grown in importance, constituting 7% as a share of total health spending in 2014.^222^ Countries that effectively utilise donor funding generally have stronger health systems, governance, and financing.^227^ However, the performance of donor funding in Nigeria has been limited by weak government coordination of donor activities, with sustainability also adversely affected by the failure of some state governments to pay counterpart funds, lateness in payment, and the problem of donor fatigue.^228^ The final major category for health financing is prepaid health spending, largely consisting of spending on private health insurance. Prepaid health spending has remained extremely low over time, averaging 3% of total health expenditure between 1995 and 2014, compared with 21% for sub-Saharan Africa over the same period.^222^ Although there were slight increases in the share of private health insurance in total health expenditure between 1995 and 2002, the share has fallen and is low, falling to near its lowest point at 0·8% in 2014 (compared with 21% for sub-Saharan Africa in 2014).

Health financing reform will be essential if Nigeria's health system is to deliver universal health coverage to its population, as the current spending is neither evidence nor impact driven, with inequitable distribution of resources, dysfunctional systems to protect individuals and households from catastrophic expenditure, and little attempt to minimise wastage*.*^228^ Reforms should focus on increasing government funding for health, improving resource management through strategic purchasing, altering the National Health Insurance Scheme (NHIS) legislation to require mandatory insurance coverage using a revised and more robust benefit package, and establishing strong systems for oversight and regulation of providers such as Health Maintenance Organisations. ^228^ Ultimately, to improve financial risk protection and the effectiveness of health financing mechanisms such as social health insurance in Nigeria, implementation bottlenecks must be addressed within the three health financing functions, which are revenue mobilisation, pooling, and purchasing.

#### Options for revenue mobilisation

Fiscal space for increased domestic funding of health services requires increases in overall government revenue and expenditure, and increasing share of government resources being devoted to the health sector.^229^ To increase the fiscal space, Nigeria also needs to rapidly diversify its economy—for example, by developing the digital, global, and local services sector and manufacturing—to ensure continued and increasing government revenues. As history has shown, the over-reliance on oil revenue for foreign exchange exposes the country to continuous financial shocks. Building and improving government institutions and corporate governance structures will increase investor confidence and promote foreign investments to strengthen Nigeria's macroeconomic foundations.^230^ Panel 10 provides ideas for expanding fiscal space and optimising government health expenditure in Nigeria.

Panel 10 **Increasing fiscal space and improving government expenditure on health in Nigeria**

**Increasing fiscal space for health**

• Improve tax collection: link national identification numbers with government-delivered benefits, banking, and government-issued permissions; institute automated pay as you earn tax collection via electronic payments
• Diversify sources of government revenue (80% of Nigeria's government revenue comes from oil receipts)^35^
• Institute health taxes on alcohol, tobacco, gambling, sugary drinks, fatty foods, luxury goods, flying, oil and gas profits, and telecommunications and social media
• Impose a levy on individuals seeking foreign exchange to pay for health treatment overseas
• Increase GDP by diversifying economic activity and increasing investor confidence
• Dedicate a proportion of already available VAT to social health insurance

**Increasing government expenditure on health**

• Institute legally-binding government commitments to distribute ring-fenced health funding, backed by sustained and focused lobbying from national stakeholders and the general public for universal health coverage
• Create incentives for fully implementing detailed costed health plans by local and state governments
• Incentivise delivery of programmes with rewards and promotions for health-care managers and enforce penalties for programme failures
• Legislate for open access to data on programme delivery and expenditure to allow scrutiny by the public at the local and national levels
• Institute universal government pre-paid health insurance for children, pregnant women, and vulnerable groups
• Improve government expenditure on health—greater health for the same resources
• Conduct studies to understand opportunity costs of alternative options for health expenditure to maximise health outcomes for a given amount of resources

Any consideration of fiscal space typically entails an examination of whether and how a government could feasibly increase its expenditure in the short-to-medium term.^231^ The tax-to-GDP ratio in Nigeria is very low, leaving scope for increasing domestic resource mobilisation through tax collection. The low tax-to-GDP ratio is associated with inadequate compliance and enforcement, weak revenue administrations, low taxpayer morale, poor governance, and corruption.^232^, ^233^ However, a multicountry study including Lagos state in Nigeria^232^ showed that increased tax revenue led to increases in public health spending in absolute although not necessarily real per capita terms. Indeed, the percentage of the government budget allocated to health declined for much of the period under review due to intersectoral competition in priority setting, fiscal federalism, Ministry of Finance perceptions of the health sector's absorptive capacity, weak investment cases made by the Ministry of Health, and insufficient parliamentary and civil society involvement. Ministries of Health, including Nigeria's FMoH and state-level Ministries of Health, must therefore strengthen their ability to negotiate for larger—and ring-fenced—allocations from government revenue through better planning, while showing improved performance and adequate absorptive capacity and making the case for the benefits of health investments.

Health has always been a stated national policy priority for the federal government and some state governments, as evidenced by numerous statements and policies including the 1995 Health Summit, the revisions of the National Health Policy in 2004 and 2016, the Health Sector Reform Programme (2003–07), commencement of the Formal Sector Social Health Insurance Programme of the National Health Insurance Scheme in 2005, the Office of the Senior Special Assistant the President on MDGs (Millennium Development Goals)—National Health Insurance Scheme Free Maternal and Child Health programme, the Midwives Service Scheme, the Presidential Summit on universal health coverage in 2014, the enactment of the Health Act with a dedicated amount of special funds (Basic Health Care Provision Fund), the development of the National Health Financing Policy in 2016, and the development of the National Strategic Health Development Plans 1 and 2, and the SURE-P MCH programme, among other initiatives. Differing emphases and funding streams for some diseases, such as AIDS, tuberculosis, and malaria,^234^, ^235^ or for select modes of care (medical compared with surgical services), have nonetheless created noticeable pathology-dependent variations in financing and access to care.

Revenue disbursement, largely from oil receipts, is extremely centralised in Nigeria. Apart from a constitutional requirement that 13% of gross oil revenue be shared among oil producing states in proportion to their production volumes, all revenues are remitted to the federal government.^236^ The remaining revenue is paid into a federation account, which also gathers revenues from corporate income taxes, custom and excise duties, and notably, value-added tax (VAT) revenue from state governments. Given the substantial share of revenues from oil in the account, the gross amount in the account fluctuates closely with exogenous changes in the export price of oil.^236^ Revenues are then shared by the federal government among the three levels of government according to a vertical and horizontal formula. State and local governments, including oil-producing states, have no control over the rate of federal allocations; the only tax revenue they directly raise and control is internally generated revenues, largely from personal income taxes and business registration and land leasing fees.^236^ States are thus heavily dependent on federal transfers for revenue; for example, between 2000 and 2016, transfers from the federal government comprised 81% of state revenues on average, with the result that political considerations often prevailed over population health needs in setting states' spending agendas.^236^

Health spending would therefore be improved by instituting a dedicated pre-determined budget at the federal and state levels, outside of the electoral cycle, and with mechanisms to ensure it is spent efficiently and equitably. The budget must be made public and subject to independent auditors to ensure equity in the distribution of resources and setting of health priorities. An increase in states' internally generated revenues would also lower state dependence on federal funding and refocus priorities on internal needs in determining health spending. A transparent, public process for assigning and using grants from international donor partners, subject to independent regular audits, is also needed. To address some of these issues, in 2014, Nigeria established the Basic Healthcare Provision Fund, financed by an annual federal government grant of not less than 1% of the Consolidated Revenue Fund, grants from external donors, and other sources. 50% of the funds gathered are to be administered by the NHIS to provide basic health services to citizens and for subsidy payments to state insurance agencies to provide health care to the very poor who are unable to afford premium payments; however, more far-reaching reform is needed to reach Nigeria's goal of universal health coverage.

#### Using pooling and insurance systems to manage revenues and reduce the burden on poor Nigerians

Pooling is the health financing function whereby collected health revenues are transferred to purchasing organisations, which manage revenues and distribute risks. Nigeria should strive to develop large pools because having small, scattered, and uncoordinated pools will not lead to efficient and equitable financial risk protection. However, in the case of multiple pools, such as the various State Social Health Insurance Schemes, the Formal Sector Social Health Insurance Programme, free programmes funded by the budget, community-based health insurance schemes, and private health insurance, risk equalisation can be achieved via mechanisms including a dedicated fund and health re-insurance, under the leadership of the Federal Ministry of Finance, FMoH, the National Council of Health, and the Nigeria Governors Forum.

To further improve pooling and management of revenue, the federal, state, and local governments should ensure the development and institutionalising of efficient, equitable, and transparent fund management systems. Development partners should move from their current opaque systems to ensure the pooling of donor funds that will be transparently managed. The government, through health and finance ministries, should ensure harmonisation and alignment of donor funding to health with national policies, strategies, and priorities. Third-party funds pooling agents can be public, quasi-public, or private entities depending on the context and preferences of the different levels of government.

Furthermore, the federal government needs to amend the legislation that established NHIS and revise the benefit package so that every citizen is covered by social health insurance, and so that it is implemented with strict oversight and regulation of Health Maintenance Organisations.^228^ Awareness and benefits of social health insurance should be increased and should be mandatory for all. At regular intervals, the NHIS's implementation strategy should be reviewed to fast track and improve the level of coverage among informal and formal sector workers, with the poorest Nigerians covered by government, with the objectives of providing universal financial risk protection and eliminating both the high level of out-of-pocket and the proportion of expenditure it covers. Federal and state-specific strategies should address context-related challenges of individual states (eg, the inability to reallocate funds into the Formal Sector Social Health Insurance Programme).

In keeping with our recommendations of localising particular aspects of health services provision, it is important to allocate more funds at the state and local government levels for purchasing health services, with evidence-based, strategic, and appropriately-tracked spending^137^ to ensure resources are used efficiently while removing financial barriers to access by reducing out-of-pocket expenditure in both absolute and relative terms.^136^ Innovative strategies are also needed to enable potential beneficiaries, especially in the informal sector, to better comprehend and accept the concept of prepayment methods of financing health care, and ensure all the formal sector employees are adequately informed about the Formal Sector Social Health Insurance Programme of the NHIS. State and local governments can establish a tax-based health financing mechanism targeted at vulnerable groups, the poorest groups, and individuals working in the informal sector of the economy to accelerate progress towards universal health coverage. Lessons can be learned from health insurance schemes in Ghana and Anambra State about potential strategies to expand health insurance coverage among informal sector workers (panel 11).

Panel 11 **Lessons on covering informal sector workers with health insurance in Ghana and Anambra State, Nigeria**

Ghana's National Health Insurance Scheme (NHIS) has been running for 16 years, and the Anambra State Health Insurance Scheme (ASHIS) has been in existence only since 2016. They both began in response to growing out-of-pocket health spending and stagnated government spending on health. Each scheme is mandatory by law, with different approaches to addressing the needs of people who are extremely poor, many of whom work in the informal sector. In less than 3 years, Anambra state health insurance scheme had covered over 100 000 active enrolees. Although this makes up only 2·5% of the population of Anambra, 36% of the enrolees are in the informal sector. Ghana's NHIS covers about 41% of the population and 29·8% of NHIS members are informal sector workers.

In Anambra, people in the informal sector are expected to pay a membership premium, but there is a Philanthropists Adoption Model that targets high-income residents of the state to purchase annual subscriptions for low-income indigenes through a mass campaign, enabling the scheme to capture a large chunk of the informal sector. The NHIS in Ghana is funded through a 2·5% tax on goods and services, 2·5% deduction from formal sector workers' social security contributions, and premiums from informal sector workers (paid progressively based on their income level, ranging from GHS7·20 (US$1·62) to GHS48 ($10·83) per person per year). Revenue from taxes funds about 70% of the scheme, whereas 23% comes from social security deductions, 5% from premiums, and 2% from sources such as donors. However, the population exempt from paying premiums is so large—for example, children (<18 years), pregnant women, and people living in extreme poverty—that it accounts for 65% of the total population covered by the scheme.

Anambra State's Health Insurance Agency has offices in all the state's 21 Local Government Areas (LGAs). The roll-out of the scheme was preceded by mass sensitisation on traditional and social media and in all health facilities, with an ongoing 24-hour call centre (for real-time resolution of enrolee or provider challenges) and annual Town Hall meetings with stakeholders and enrolees (to discuss and address service delivery issues). Like Anambra state, Ghana's Health Insurance Agency has offices in all districts of Ghana, through which people register for and engage with the public scheme, which operates alongside private schemes. In 2012, a central funding pool was created by combining district pools, which were too small to be sustainable and too inefficient to operate. Premiums are paid into the central pool, and the government provides subsidies to cover exempt groups.

Registration (including self-registration) of enrolees in the Anambra state health insurance scheme is done electronically using a locally developed insurance information management system equipped with biometric and facial identification features. In Ghana, all members of the NHIS register in person at a district office and renew their membership yearly in a process that includes an in-person interview to determine exempt status and biometric data collection. A new mobile renewal service, which allows members to use an SMS code and mobile money to renew their membership, has been linked to a recent surge in enrolment—from 10·2 million in 2017 to over 12 million in 2019. In both Ghana and Anambra, electronic platforms reduce the challenge of enrolees forgetting renewal dates, and the costs associated with in-person registration.

Most people in Ghana and Anambra state still do not enrol and many do not renew their membership due to the cost of premiums, challenges in belonging to the large informal sector, and the weak administrative capability of insurance agencies. In Ghana, members are not required to contribute any co-payments, co-insurance, or deductibles. In Anambra state, another reason for low membership is delays in reimbursement of both enrolees' and providers' claims, which causes dissatisfaction, particularly among poorer enrolees who have difficulty paying out of pocket. These issues are addressed through the 24-hour hotline for complaints and feedback, a feature which has generated much public support and confidence in the scheme. In Ghana, client expectations are managed at the district level through community outreach and education. Expanding the reach of insurance requires innovations that create convenient, quality, and timely services for providers and clients.

The government, through the FMoH, has tried to invest in improvements in health-care access by introducing policies like the Formal Sector Social Health Insurance Programme of the NHIS in 2005, aimed at improving health access through increasing uptake and coverage of health insurance in the country.^237^ The Formal Sector Social Health Insurance Programme is a prepaid plan in which participants pay fixed regular amounts or premiums, which are then pooled and transferred to Health Maintenance Organisations to cover the cost of health care. Although the objective is to share risk, uptake of the Formal Sector Social Health Insurance Programme and other health insurance schemes of the NHIS, like private prepaid insurance at 0·8%, has been low, with information asymmetry on the part of both citizens and health providers, and concerns about moral hazard and adverse selection by market providers impeding insurance uptake.^237^

Private health insurance schemes often do not cover individuals most in need, although they can be a useful stopgap before sufficient public provision is secured. Encouraging the use of mobile payments and apps to make and process insurance claims could reduce barriers to insurance coverage for some individuals, especially in urban areas,^238^ however, the larger barrier is poverty, meaning insufficient total premiums can be pooled to fund sufficient services for individuals most in need.^239^ The funding gap for the National Strategic Health Development Plan 2 2018–2022 moderate coverage scenario ($6·8 billion of a total estimated cost of $19·9 billion) could have been bridged via increasing state health insurance subscribers from 5% in 2018 to 30% in 2022,^11^ however the subscriber base remained low at 5% in 2020.^240^

Reducing the burden and financial risk from health-care spending for individuals, families, and communities will therefore require a dramatic provision of and access to pooled funding (insurance) or pre-paid government provision of health care. Alternatively, mandatory social insurance for health could be levied, although both would need to be done equitably to guard against increasing inequality and resulting health, social, and economic harms. Additionally, solutions for barriers to coverage of those working in the informal sector and individuals unemployed would need to be found.^240^ Levying taxes and social insurance is also difficult as the majority of work is in the informal sector in Nigeria. However, as has been seen in other countries such as India and Ethiopia (panel 8), should universal health coverage become an election issue nationally and at state level, concerted advocacy and community engagement efforts from committed institutions and civil society organisations could provide creative approaches to facilitate universal health coverage.^241^

#### Using strategic health purchasing to increase efficiency

Strategic health purchasing ensures that only needed services are purchased by identifying the most cost-effective ones with evidence of good value for money, and those essential for achieving health-related SDGs targets, universal health coverage, and other national priorities.^242^ The objectives of strategic purchasing are to enhance equity in the distribution of resources, increase efficiency (more health for the money), manage expenditure growth, and promote quality in health service delivery.^243^ Strategic health purchasing serves to enhance transparency and accountability of providers and purchasers to the population, which contributes to the ultimate goals of maximised health outcomes and equity in health gains, financial protection, and equity in financing as well as responsiveness.^243^, ^244^

Countries at all levels of income are considering or implementing reforms to increase the extent to which purchasing of services in the health system is strategic.^243^ Strategic health purchasing helped to achieve a pro-poor utilisation of health services in Thailand,^245^ equitable and high level of use of free maternal and child health services in Nigeria,^246^ and in many other health services in other countries.^247^ However, it is noted that improving the purchasing function is a continuous challenge for health system governance and require adaptations in how best to purchase services over time.^243^, ^245^, ^247^

Hence, strategic health purchasing should be adopted at all levels by all public and quasi-public purchasers, and become the main mechanism used by purchasers such as federal and state Ministries of Health, agencies, and other third-party financiers in the Nigerian health sector. In preparing budgets, government at all levels should ensure the institutionalisation of the medium-term sector strategy,^248^ introduced by the Budget Office in 2006, which outlines strategic objectives, activities, and budgets in the allocation of public sector health expenditure at all levels. Although the medium-term sector strategy is designed to achieve strategic health purchasing and lead to more efficient, equitable, and accountable deployment of resources, its implementation has been stalled and should become a top priority.

A broad-based, participatory medium-term sector strategy process will involve non-state actors such as civil society organisations, the private sector, consumer groups, and development partners to develop annual budgets and decide the use of other resources to purchase health services. Federal and state governments should entrench strategic health purchasing as the main mechanism for purchasing health services.^249^ The institutionalisation of strategic health purchasing within Nigeria will require strengthening the capacity of purchasing agencies and raising awareness of its benefits among decision makers in the Ministry of Finance and various departments, agencies, and programmes at the Ministries of Health at the federal and state levels.^228^, ^250^ One high priority strategic health purchasing intervention in Nigeria is creating the expectation that governments at all levels should purchase free services for high-priority life-saving public health services through increased use of government revenue, notably the Basic Healthcare Provision Fund. Such services will include: (1) immunisation prevention and treatment of HIV/AIDS, tuberculosis, and malaria; (2) some non-communicable diseases; and (3) maternal, neonatal, and child health services, especially antenatal, childbirth, and postnatal services (table 5, panel 9).

## Section 6: conclusions and recommendations

With Nigeria's large and growing population, ongoing governance and security challenges, and potential leadership role in Africa and globally, the decisions made today will determine whether Africa's most populous country will become a success story or a cautionary tale. A successful Nigeria will inarguably require strong investments in health, education, and basic public services, following a central organising principle of creating a healthy population (ie, a health-in-all-policies approach). Such an approach is the best pathway to human flourishing, economic development, and a legacy for the politicians who achieve it. The strength of institutions—in government, civil society, traditional and religious authorities, and within communities—can and must be harnessed to engender and reap the benefits of a virtuous cycle of prosperity and good health. Institutions, including formal structures such as the constitution and laws, and informal societal factors such as customary norms and values, are requisite to achieving the political and social accountability that continues to elude the nation. The alternative of business-as-usual risks a spiral of increasing poverty, inequality, and insecurity as the growing population is blighted by the interdependent challenges of lack of opportunity, poor education, and poor health. Delivering a health agenda for Nigeria is thus a matter of the utmost importance for Nigeria, the subregion, and the world.

In this Commission, we began by exploring how Nigeria's health system, writ large, evolved from a widely accessible community health infrastructure during the pre-colonial period, to an inegalitarian colonial inheritance, ultimately leading to a post-independence period of an unequally distributed, unbalanced, and weak health system despite multiple national plans over six decades. Nevertheless, the story of the Nigerian health system presents numerous successes that can serve as lessons to build upon. The overall progress over the past 50 years shows that most indicators have moved in the right direction, although there is much room for improvement. Further, Nigeria effectively utilised vertical programmes with international multilateral agency support to contain Guineaworm disease and wild-type poliomyelitis showing that the health system can, in particular circumstances, deliver. There is a distinct opportunity to fulfil Nigeria's constitutional promise to ensure health care to all persons and extend this to wider preventive health goals.

The giant of Africa—Africa's largest country in terms of population and economy—enjoys considerable unrealised potential. The time to achieve greatness is now, with health at the heart of the development agenda. The health system can become a positive reflection of Nigeria—with successful health reform the catalyst to show why Nigeria matters to Nigerians, giving good reason for patriotism, and serving as a model for wider societal change.

## Declaration of interests

FS, MNS, GA, and BLS are current heads of Nigerian Government health agencies. CI and SHA held leadership roles in the Nigerian Government during the period of writing of this Commission. SA is editor-in-chief of *BMJ Global Health*. IA was a Scientific and Technical Adviser to the Nigerian Government Presidential Task Force on COVID-19 and ZI is chair of the Nigerian national ethics committee. All other authors declare no competing interests.
