# Supplementary material for: The Lancet Nigeria Commission: investing in health and the future of the nation
Source: Lancet. 2022 Mar 19;399(10330):1155–200. doi: 10.1016/S0140-6736(21)02488-0 (PMC8943278; doi:10.1016/S0140-6736(21)02488-0)

THE LANCET

Supplementary appendix

This appendix formed part of the original submission and has been peer reviewed. We post it as supplied by the authors.

Supplement to: Abubakar I, Dalglish SL, Angell B, et al. The *Lancet* Nigeria Commission: investing in health and the future of the nation. *Lancet* 2022; published online March 15. https://doi.org/10.1016/S0140-6736(21)02488-0.

Web Appendix 1: Methodology for Key Informant Interviews

Part of the historical assessment that the Lancet Commission on Nigeria intends to undertake is to determine how the country's health system has evolved during these periods spanning pre-colonial to the post-colonial periods, including the periods of military and democratic rules. Our strategic objective is to answer the following questions: 1) how has Nigeria's health care system performed or oscillated between its different historical periods?; 2) are there lessons or successes or mistakes made that can help to inform the design of policies and practices for improving the country's health care system?; 3) who were the systemic actors and which institutions have helped or did not help Nigeria's health care evolution?; and 4) how can the country overcome the mistakes of the past in moving forward with a more proactive and resilient health care system?

We sought answers to these and related questions by conducting desk reviews of available publications relating to Nigeria's history and health development. Additionally, the panel undertook qualitative interviews with key informants, especially practitioners and knowledgeable agents in the country's health care delivery system, to elicit their knowledge, understanding, and perceptions of the trajectories over time. We specifically asked the interviewers to focus on identifying the challenges that the health care system has faced, and to proffer recommendations on ways to rectify the challenges.

Data source

Key informant interviews were conducted with key players in the Nigerian health care system.

Participants and recruitment

All participants were recruited purposefully. The criteria for selection were that the participant is currently or was in a key position in Nigeria's health sector and/or possessed professional training and experience in health service delivery in Nigeria. We approached up to 25 key informants based on their knowledge of the historical development and current state of Nigeria's healthcare system. Only those who accepted the fully explained protocol (n=10) were interviewed. See Table S1 below for a brief description of the informants. Of the 10 participants, 2 were non-doctors, one of whom was a former Minister of Health.

Table S1. Description of the participants

S/N	Archival Code	Position/Designation
1	KII01	Professor of Community Medicine
2	KII02	Former Minister of Health
3	KII03	Professor of Medicine
4	KII04	Professor of Medicine
5.	KII05	Minister of State for Health
6.	KII06	Professor of Cardiology
7.	KII07	Physician
8.	KII08	Former Minister of Health
9.	KII09	Retired Professor of Medicine
10.	KII10	Traditional ruler

Interview Guide

The idea to conduct the key informant interviews (KII) was accepted at a meeting of members of the Lancet Commission at a meeting held in London in January 2020. Thereafter, a member of the Commission, FEO, drafted a KII interview guide and circulated to other members of the commission. Comments were received which informed the final draft of the guide. The guide (shown in Appendix 1) consisted of 3 sections. In section 1 of the guide, we asked questions on the history of Nigeria and its health care system from the precolonial to the colonial periods, and specifically requested information on flash-points and illustrative success stories. In the second part, we fielded questions on Nigeria's history and its health care after independence (post-colonial period), and asked for information on actors, institutions, and key health policies that shaped health development during the period. We specifically asked questions on military rule and its consequences for health care, and also focused on the country's economy, financing mechanisms and debt burdens during the period and their implications for health care. In the final section of the guide, we asked specific questions on the performance of the various health reform measures that the country had undertaken over the years. These included questions on universal health coverage, primary health care, health policies and their implementations, health insurance, structural adjustment, and debt relief gains. In this final section, we also requested the informants to enumerate the challenges the country had faced in the provision of health care, and to identify their perspectives on ways to address the challenges. All questions were fielded in a value-free, and open-ended manner without any attempt to teleguide the answers, while the interviewers probed for further answers to fully illuminate the questions.

Data collection procedure

All the interviews were conducted through Zoom by one of the members of the Lancet Nigeria Commission, Professor Okonofua, and his assistant. The interviews were tape-recorded and transcribed verbatim. The average duration of the interviews was one hour and each interview ended when no further issues arose. All interviews were conducted in English, tape-recorded, and transcribed verbatim. Being a qualitative study, our sampling strategy focused on interviewing knowledgeable informants until reaching saturation. As such, we conducted the interviews and analysed them concurrently until the same information was being repeatedly mentioned by the interviewees.

Data analysis

The data were analysed by an expert in qualitative data analysis, and Professor Okonofua validated the data. All the transcripts were coded using Atlas.ti version 6.2.25. The analysis followed an iterative, deductive and inductive approaches to thematic analysis. The analysis was conducted as data were collected, and data collection continued until data saturation. Codes were generated from the interview guide and the project objectives, and the themes emerging from the narratives. Each transcript was coded after reading the transcripts several times to become familiar with the data. Similar codes were merged, and all the codes were grouped into sub-themes, and main themes.

Presentation

The results are presented thematically in narratives with apt quotations. Some results were presented in a network view as figures where necessary. There are four main themes: the history of

health and the health system in Nigeria, the major actors and the key milestones; the challenges of the Nigerian health care system; key policy flashpoints; and recommendations on ways to improve Nigeria's health care system. The challenges are presented using 21 sub-themes, the recommendations on ways to improve health care delivery and the health system are presented using 23 sub-themes, training of doctors is further presented in 4 sub-categories.

Ethical consideration

All the key informants were contacted in writing with detailed information about the Lancet Nigeria Commission project. The consent of each informant was obtained before the interview. All direct identifiers are removed from the report.

Web Appendix 2: Delphi process for selecting priority disease burden areas

Given the paucity of data and existing burden of disease analysis for Nigeria, the Burden of Disease Group undertook a prioritisation process to identify the most important areas for the group and commission to focus their efforts and analysis.

Methods

We conducted a modified electronic-Delphi (e-Delphi) priority-setting exercise with members of the Lancet Nigeria Commission and other key stakeholders in the Nigerian health system. The exercise was conducted using Survey Monkey over two rounds.

Round 1 – Prioritisation

The first round of the e-Delphi process asked respondents to rank the importance of further work examining conditions and risk-factors against four criteria:

1. **The Magnitude of Need –** to assess how important an issue the condition or risk factor was to the Nigerian population and health system.
2. **Available Knowledge** – to assess the importance that further knowledge of the burden of the condition for the Nigerian population and health system.
3. **Leverage –** to assess the potential for our work in this area to contribute to strengthening the Nigerian health system.
4. **Equity –** to assess whether work to address the specific condition or risk-factor would likely also act to reduce disparities across the population.

These criteria were adapted from the existing literature by a working group within the Burden of Disease (BoD) Working Group to include the most important factors the group was looking to consider.[1,2] Respondents were presented with a short description of each condition or risk-factor including mortality and morbidity (in for form of disability adjusted life years (DALYs)) estimates from the Global Burden of Disease Study and asked to rank the condition or risk-factor on a scale of 1-5 for each criteria by either typing a response or dragging a slide bar between 'Lowest Priority' and 'Highest Priority' (with the equivalent number displayed on the screen as they did so). They were also asked to identify any priority conditions for work within each category. An example question for maternal and neonatal conditions as presented to respondents is shown in Figure 1. Participants were asked to respond to these questions for 17 groups of conditions and 13 of risk factors. At the end of the survey respondents were asked if they would like to suggest any other conditions or risk factors that deserved further attention that we had not already asked about. Participants were given 1 week to respond. Average scores across all criteria and overall for each condition or risk-factor were calculated to feed into Round 2.

Round 2 - Validation

Results from Round 1 were used to identify conditions and risk-factors considered of highest priority and new conditions or risk-factors provided by respondents for inclusion in the second round. The LNC Burden of Disease Group reviewed all suggested conditions and risk-factors that weren't included in the first round and decided to include one new condition category: '*Diseases with epidemic potential (not elsewhere categorised)*'. A ranked list was presented to respondents along with the priority score from the first round (for all conditions ranked in the first round) and they were asked if they agreed with the ordering of the conditions or risk-factors as presented. If they selected 'yes' there were no further questions but if they responded 'no' they were asked to rearrange the list in order of priority (by either selecting the rank for each condition or risk-factor or

by dragging and dropping the list into the appropriate order). Figure 2 shows the question presented to respondents for the conditions.

Results

The survey was sent to 59 people comprising prominent Nigerian academics, health-sector policymakers and clinicians. Specifically, 47 academics (with expertise in various clinical disciplines, public health, public policy, health economics, history and other social sciences), 6 policymakers and 6 clinician-researchers working in the health system were approached through the e-Delphi process.

Round 1

23 people responded to the survey. The average scores from all respondents are presented in Table 1 and 2. Maternal and neonatal conditions received the highest average score of the conditions across all domains except for 'Available Knowledge' where cardiovascular diseases and diabetes and chronic kidney diseases received slightly higher average scores. While Maternal and neonatal conditions were also attributed with the highest burden in terms of DALYs in the Global Burden of Disease study, the results of the other conditions differed from the GBD rankings, for example cardiovascular diseases and diabetes and chronic kidney diseases were ranked as second and third most important areas for further research by respondents but rank fifth and ninth respectively in the GBD study.

For the Risk Factors, Child and maternal malnutrition, Unsafe water, sanitation and handwashing and High-systolic blood pressure received the highest average scores respectively. A number of specific suggestions were made for conditions to focus on with the categories which are presented in Appendix 1. There were also suggestions made for new areas to include in Round 2. The BoD Group assessed these and decided that most fit into existing categories apart from '*Diseases of Epidemic Potential*' suggested by one respondent. This was included in the second round of the process.

Round 2

The top 11 condition groups and the additional category (*Diseases with epidemic potential (not elsewhere categorised)*) and 5 risk-factor categories were presented to respondents. In total 28 respondents (out of 59 total invitations) completed the survey. The results of this round are presented in Table 2 and 3 alongside the rankings obtained from Round 1 and the GBD estimates. For the conditions, 54% (15) agreed with the prioritised list of conditions as presented and the remainder changed the order. The most common arguments made by respondents who changed the order highlighted the importance of traffic accidents in Nigeria and diseases of epidemic potential (both raised by 4 respondents). All responses provided are included in Appendix 2.

For the Risk Factors, 68% (18) of respondents agreed with the list of risk factors as initially prioritised while 9 changed the order. Everyone who changed the order ranked air pollution as the lowest priority (and agreed on other rankings apart from 2 who thought unsafe water, sanitation and handwashing should be the top priority). Once again, the reasons provided are included in Appendix 2.

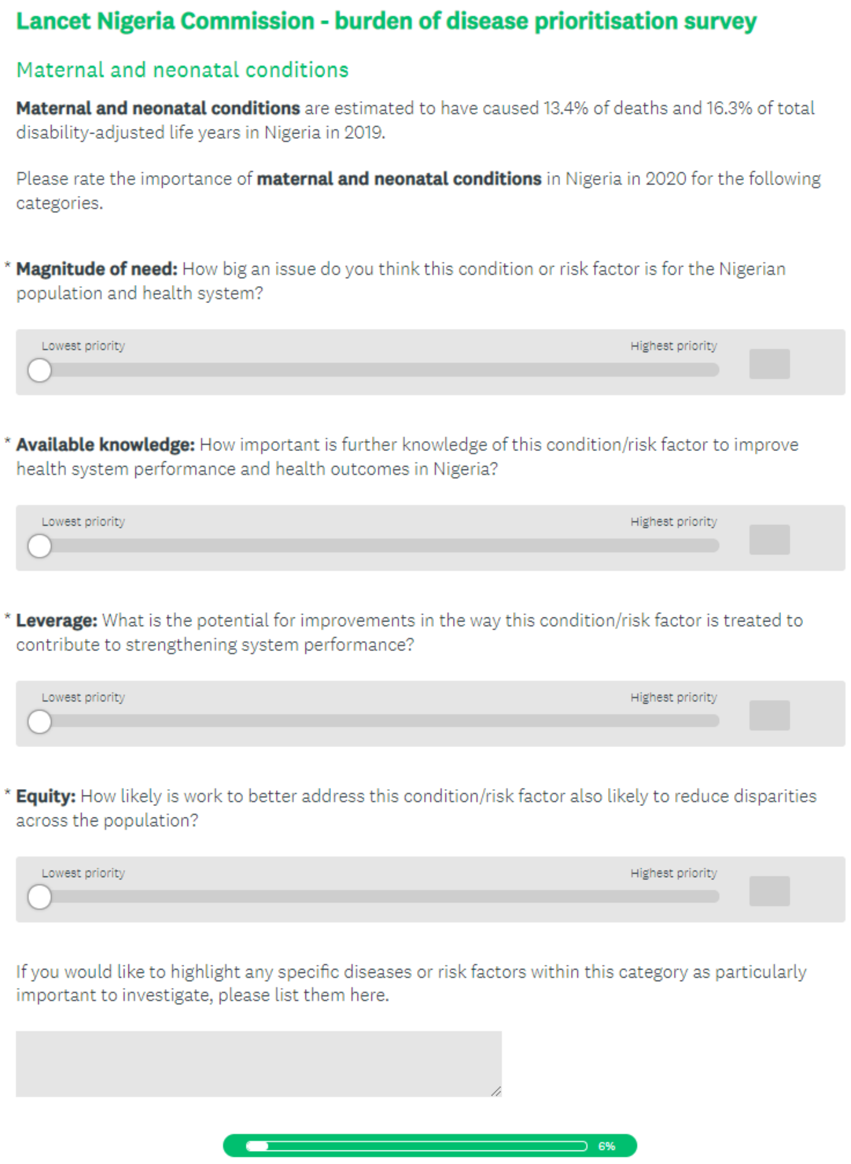

Lancet Nigeria Commission - burden of disease prioritisation survey

Maternal and neonatal conditions

Maternal and neonatal conditions are estimated to have caused 13.4% of deaths and 16.3% of total disability-adjusted life years in Nigeria in 2019.

Please rate the importance of **maternal and neonatal conditions** in Nigeria in 2020 for the following categories.

* **Magnitude of need:** How big an issue do you think this condition or risk factor is for the Nigerian population and health system?

Lowest priority | Highest priority

* **Available knowledge:** How important is further knowledge of this condition/risk factor to improve health system performance and health outcomes in Nigeria?

Lowest priority | Highest priority

* **Leverage:** What is the potential for improvements in the way this condition/risk factor is treated to contribute to strengthening system performance?

Lowest priority | Highest priority

* **Equity:** How likely is work to better address this condition/risk factor also likely to reduce disparities across the population?

Lowest priority | Highest priority

If you would like to highlight any specific diseases or risk factors within this category as particularly important to investigate, please list them here.

6%

Figure S1 - Example question from Round 1 of the prioritisation process

Lancet Nigeria Commission - Second Round Prioritisation Survey

Burden of Disease - Priority Conditions

In the first round of this prioritisation process, respondents identified 11 condition groups as high-priority and one additional category was put forward for consideration (diseases with epidemic potential, not otherwise classified). We are interested in your thoughts on the relative importance of these conditions to Nigeria in 2020, which are listed below from highest to lowest priority according to the scores assigned in the previous round (out of a maximum score of 5).

**Prioritised Conditions**	**Priority score**
1. Maternal and Neonatal Conditions	**4.30**
2. Cardiovascular diseases	**3.91**
3. Diabetes and Chronic Kidney Diseases	**3.77**
4. Neglected Tropical Diseases and Malaria	**3.75**
5. Respiratory Infections and TB	**3.74**
6. Neoplasms	**3.71**
7. Mental Disorders	**3.64**
8. Enteric infections	**3.62**
9. Transport Injuries	**3.58**
10. Nutritional deficiencies	**3.48**
11. HIV/AIDS and Sexually Transmitted Infections	**3.39**
12. Diseases with epidemic potential (not elsewhere categorised)	**Unranked**

* 1. This list will be used to prioritise the work of the commission. Do you agree with the ordering of the conditions presented here (from most to least important to Nigeria in 2020)?

◯ Yes

◯ No

Figure S2 - Example question from Round 2 of the prioritisation process

Prioritised list of Conditions	Magnitude of need score	Available Knowledge score	Leverage Score	Equity score	Overall score
**1. Maternal and Neonatal Conditions**	4.70	3.91	4.17	4.43	**4.30**
**2. Cardiovascular diseases**	4.30	4.04	3.78	3.52	**3.91**
**3. Diabetes and Chronic Kidney Diseases**	4.00	4.00	3.55	3.55	**3.77**
**4. Neglected Tropical Diseases and Malaria**	3.83	3.61	3.61	3.96	**3.75**
**5. Respiratory Infections and TB**	4.17	3.61	3.61	3.57	**3.74**
**6. Neoplasms**	3.65	3.87	3.61	3.70	**3.71**
**7. Mental Disorders**	3.73	3.73	3.45	3.64	**3.64**
**8. Enteric infections**	3.87	3.48	3.52	3.61	**3.62**
**9. Transport Injuries**	3.95	3.45	3.50	3.41	**3.58**
**10. Nutritional deficiencies**	3.52	3.52	3.14	3.71	**3.48**
**11. HIV/AIDS and Sexually Transmitted Infections**	3.39	3.17	3.30	3.70	**3.39**
**12. Unintentional Injury**	3.23	3.05	3.00	3.09	**3.09**
**13. Neurological Disorders**	3.09	3.32	2.86	2.86	**3.03**
**14. Self harm and violence**	3.00	3.00	2.64	2.73	**2.84**
**15. Chronic respiratory diseases**	2.68	3.05	2.82	2.50	**2.76**
**16. Digestive diseases**	2.48	2.83	2.43	2.30	**2.51**
**17. Musculoskeletal disorders**	2.19	2.43	2.14	2.05	**2.20**

Table 1 - Average scores from Round 1 of the prioritisation process for Condition groups

Risk Factor	Magnitude of need score	Available Knowledge score	Leverage Score	Equity score	Overall score
**1. Child and maternal malnutrition**	4.57	4.19	4.19	4.52	**4.37**
**2. Unsafe water, sanitation and handwashing**	4.52	4.10	4.05	4.67	**4.33**
**3. High-systolic blood pressure**	4.05	3.71	3.19	3.62	**3.64**
**4. Air Pollution**	3.76	3.76	3.29	3.52	**3.58**
**5. High fasting plasma glucose**	3.38	3.14	3.05	2.95	**3.13**

**6. Kidney dysfunction**	3.00	3.24	2.67	3.05	**2.99**
**7. High body-mass index**	3.10	3.05	2.81	2.76	**2.93**
**8. Unsafe sex**	3.14	2.71	2.71	3.05	**2.90**
**9. Dietary risks**	3.05	2.86	2.67	3.00	**2.89**
**10. Tobacco use**	2.81	2.57	2.48	2.29	**2.54**
**11. Alcohol use**	2.67	2.62	2.29	2.33	**2.48**
**12. High LDL cholesterol**	2.38	2.67	2.14	2.38	**2.39**
**13. Non-optimal temperature**	2.00	1.90	1.90	2.05	**1.96**

Table S2 - Average scores from Round 1 of the prioritisation process for Risk Factor groups

**Condition group**	**GBD Rank (DALYs)**	**Rank from Round 1**	**Average rank of those who re-ordered**	**Average rank of all respondents**
**Maternal and Neonatal Conditions**	1	1	1.23	**1.11**
**Cardiovascular diseases**	5	2	2.31	**2.15**
**Diabetes and Chronic Kidney Diseases**	9	3	4.31	**3.63**
**Neglected Tropical Diseases and Malaria**	4	4	5.31	**4.63**
**Respiratory Infections and TB**	3	5	5.92	**5.44**
**Neoplasms**	7	6	6.85	**6.41**
**Mental Disorders**	15	7	8.00	**7.48**
**Enteric infections**	2	8	9.00	**8.48**
**Transport Injuries**	14	9	8.08	**8.56**
**Nutritional deficiencies**	16	10	8.69	**9.37**
**HIV/AIDS and Sexually Transmitted Infections**	6	11	8.92	**10.00**
**Diseases with epidemic potential (not elsewhere categorised)**	10	NA	9.38	**10.74**

Table S3 - Average ranks from Round 2 of the prioritisation process for Condition groups

**Risk Factors**	**GBD Rank (DALYs)**	**Rank from Round 1**	**Average rank of those**	**Average rank of all respondents**

			that re-ordered	
**Child and maternal malnutrition**	1	1	1.22	1.07
**Unsafe water, sanitation and handwashing**	2	2	1.78	1.93
**High-systolic blood pressure**	4	3	3.00	3.00
**Air Pollution**	3	4	5.00	4.33
**High fasting plasma glucose**	6	5	4.00	4.67

Table S4 - Average ranks from Round 2 of the prioritisation process for risk factor groups

Web Appendix 3: GBD analysis methods

The data sources, strategies implemented to ensure data quality and external validity, statistical modelling and metrics for GBD 2019 have been reported in detail elsewhere.[4,5] Briefly, GBD 2019 lists 369 diseases and injuries and 87 risk factors for 204 countries and territories. We extracted data from GBD 2019 on the mortality and morbidity of conditions and risk-factors prioritised through the modified e-Delphi process. Health loss related to specific causes of diseases and injury were reported as estimates of the mortality, years of life lost (YLLs), years lived with disability (YLDs), and disability-adjusted life-years (DALYs). Historical estimates of the burden of all prioritised causes were extracted 1998 [we might want to drop the first year?]-2019. Other key health outcomes were also extracted to examine how the Nigerian health sector had performed over time. These were the maternal, neonatal and under-five mortality rates and numbers of deaths, the specific causes of maternal deaths over time and deaths associated with cardiometabolic risk factors over time.

Data Sources and Analyses

The overall approach used to estimate causes of death, disease incidence and prevalence, risk factors is the same as for GBD 2019. GBD relies on 1434 relevant data sources: censuses, household surveys, civil registration and vital statistics, disease registries, health service use, disease notifications, and other sources sourced from the published literature, governments, and collaborators. Details of the major sources of data used to assess causes of death in Nigeria are shown available - http://ghdx.healthdata.org/geography/Nigeria.

Mortality and Causes of Death

Vital registration data for Nigeria are scarce and were supplemented with information from specific surveys and verbal autopsy data. All cause of death data coded using International Classification of Diseases and Injuries (ICD) were mapped to the GBD cause list. YLLs were calculated as the product of deaths and the standard life expectancy at each age to calculate. Deaths and YLLs were calculated

based on the underlying cause of deaths estimates for all 25 of 369 causes of mortality, and for age groups and both sexes.

YLDS

YLDs were computed by sequela as prevalence multiplied by the disability weights (DWs) for the health state associated with that sequela. The uncertainty ranges reported around YLDs incorporate uncertainty in prevalence and uncertainty in the DWs. To do this, we took 1000 samples of comorbidity-corrected YLDs and 1000 samples of the DW to generate 1000 samples of the YLD distribution. We assumed no correlation in the uncertainty in prevalence and DWs. Data sources used for DWs in Nigeria include the Multi Country Study Survey 2000-2001, Long version, Nigeria, 2000 - 2001 Multi-Country Survey Study on Health and Responsiveness (MCSS), World Health Organization (WHO) and data from other sub-Saharan African settings

For GBD 2019, disability weights were measured for 220 conditions covering the 1160 disease and injury sequelae. Disability weights were generated from more than 30,000 respondents collected through population-based surveys in five countries which included one African country- Tanzania. Uncertainty in the disability weight for each sequela has been propagated into the estimates of YLDs for each disease and injury. Information about age-specific mortality rates, and about overall age-specific YLDs per person were combined into the overall measure of health expectancy, HALE, using a standard approach to extending the life table to capture adjustments for non-fatal health outcomes.

Risk factors

The risk-attributable fractions of disease burden by cause were calculated by modelling the effects of risk exposure levels, documented relative risks associated with risk exposure and specific health outcomes, and computed theoretical minimum risk counterfactual levels of risk exposure on

estimates for Nigeria and other country-level deaths, YLLs, YLDs, and DALYs. The estimation of attributable mortality, YLLs, YLDs, and DALYs for 87 risk factors and combinations of risk factors, at the global level, regionally, and for 204 countries and territories in is described in detail for GBD2019. Here, we assessed the contribution of the top 20 risk factors to all−ages DALYs in Nigeria for 2019.

Web Appendix 4: Financing and Economic analysis methods and additional material

Health Expenditure

Data taken from world bank, national health accounts, national bureau of statistics

Quality of health infrastructure analysis

Low levels of government spending on health are reflected in lower quality of health infrastructure in Nigeria. The quality of health facilities within household's neighbourhoods may affect their access to vaccination outcomes and may also influence their demand for these vaccines for their children. To explore this hypothesis, we use data on health facilities collected from a 2012 survey to examine the relationship between the quality of health infrastructure and child vaccination outcomes for children born in 2012

We examined the quality of health facilities at the state level in Nigeria using comprehensive data from a 2012 survey of infrastructure and staff functionality at 24,158 mostly (97%) public health facilities. The health facility dataset comes from an effort spearheaded by the Nigerian government and researchers from the country's Office of the Senior Special Assistant to the President on MDGs (OSSAP), in collaboration with the Sustainable Engineering Lab at Columbia University who conducted extensive, comprehensive surveys of schools and health facilities at local government areas (LGAs) in Nigeria.[7] Respondents answered questions about infrastructure access, service quality and staff numbers, among other indicators, over a 1-year period that ended in 2012. The surveys were collected from respondents at health facilities across 774 LGAs in Nigeria (sampling was quasi-random, with trained enumerators assigned to data collection in each LGA; approximately 10 LGAs in the initial survey were excluded due to missing data). According to the Federal Ministry of Health, Nigeria had 34,423 health facilities as of 2013, with some 70% of them being public facilities managed by largely federal and state governments.[8,9] Thus, our health facility sample represents about 70% of the universe of health facilities in Nigeria and 97% of public health facilities in the country.

There are around 21 health facilities per 100,000 population in each state in Nigeria. **Table** S5 shows the access statistics- about 16% of facilities report having no vaccines for child immunizations. 46% of facilities have no freezer or refrigerator to store vaccines. Some 41% of facilities have no access to functional power from the national grid, with 75% of facilities reporting having generators on average. Just 43% of facilities report having access to water from an improved source like a tube-well or piped water, while only 27% of facilities have access to emergency transport vehicles. 58% of facilities report having access to family planning services like contraceptives, 73% of facilities report having antenatal services, and just 13% of facilities report having access to caesarean services for pregnant women.

Table S5 Summary statistics at the LGA level, 2012

Statistic	N	Mean	St. Dev.	Min	Max
			Child Vaccination Outcomes, 2012		
BCG	508	0.638	0.362	0.000	1.000
DPT	508	0.611	0.358	0.000	1.000
Measles	508	0.266	0.227	0.000	1.000
Polio	508	0.763	0.257	0.000	1.000
			Health Facility Quality, 2012		
Nos. of Facilities /100,000	761	21.016	19.850	0.547	390.459
Health Infrastructure Quality Index	588	0.488	0.132	0.191	0.874
No Vaccine at Facility	652	0.161	0.146	0.000	0.853
Freezer for Vaccines	588	0.457	0.352	0.000	1.000
Grid Power Access	761	0.413	0.276	0.000	1.000
Generator Power Access	717	0.748	0.287	0.000	1.000
Water Access	761	0.428	0.222	0.000	1.000
Family Planning Services	761	0.582	0.225	0.000	1.000
Ante-Natal Services	761	0.730	0.217	0.000	1.000
Caesarian Services	761	0.127	0.140	0.000	0.818
Emergency Transport	761	0.274	0.218	0.000	1.000
Share Nurses	761	0.141	0.105	0.000	0.671
Share Doctors	761	0.082	0.083	0.000	0.551
Share CHEWs	761	0.567	0.236	0.018	1.000
Share Nurse/Midwives	761	0.211	0.130	0.000	0.719
Nos. of Nurses /100,000	761	14.270	22.794	0.000	338.365
Nos. of Doctors /100,000	761	9.298	23.024	0.000	381.730
Nos. of CHEWs /100,000	761	43.982	43.028	1.068	594.957
Nos. of Nurse/Midwives /100,000	761	19.736	29.924	0.000	427.354

Notes: Health Infrastructure Quality Index is an average of 8 variables namely: the share of facilities with ante-natal services, family planning services, emergency transport, a freezer for vaccines, vaccines at the facility, caesarian services, improved water supply and grid power access.

We construct a health quality infrastructure index which is an average of 8 public services reported available at the health facility in each LGA and state in Nigeria, namely: the share of facilities with ante-natal services, family planning services, emergency transport, a freezer for vaccines, vaccines at the facility, caesarean services, improved water supply and grid power access. On average, only 49% of facilities report access to these public services (calculated as the average of the 8 measures). Using the quality of health infrastructure as a proxy for per capita health spending (with the assumption that states that spend more on health infrastructure provision, have more health infrastructure), there is a robust, positive correlation ($p<0.001$) between health infrastructure and child vaccination outcomes as shown in **Figure 15.** The bivariate correlation between child vaccination outcomes for BCG, DPT, measles and polio at the state level in 2012 is stronger than the relationship between state per capita GDP (in 2010 USD) and child vaccination outcomes, as shown by the higher R^2 measures for health infrastructure quality and BCG/DPT vaccination (0.55) versus GDP per capita and BCG/DPT vaccination (0.26) in **Figure S3** (the correlation between per capita GDP and the health infrastructure quality index is 0.42 (p < 0.001).

Figure S3. Correlations between health infrastructure quality index, GDP per capita and child vaccination by state in Nigeria, 2012, with R^2 labelled

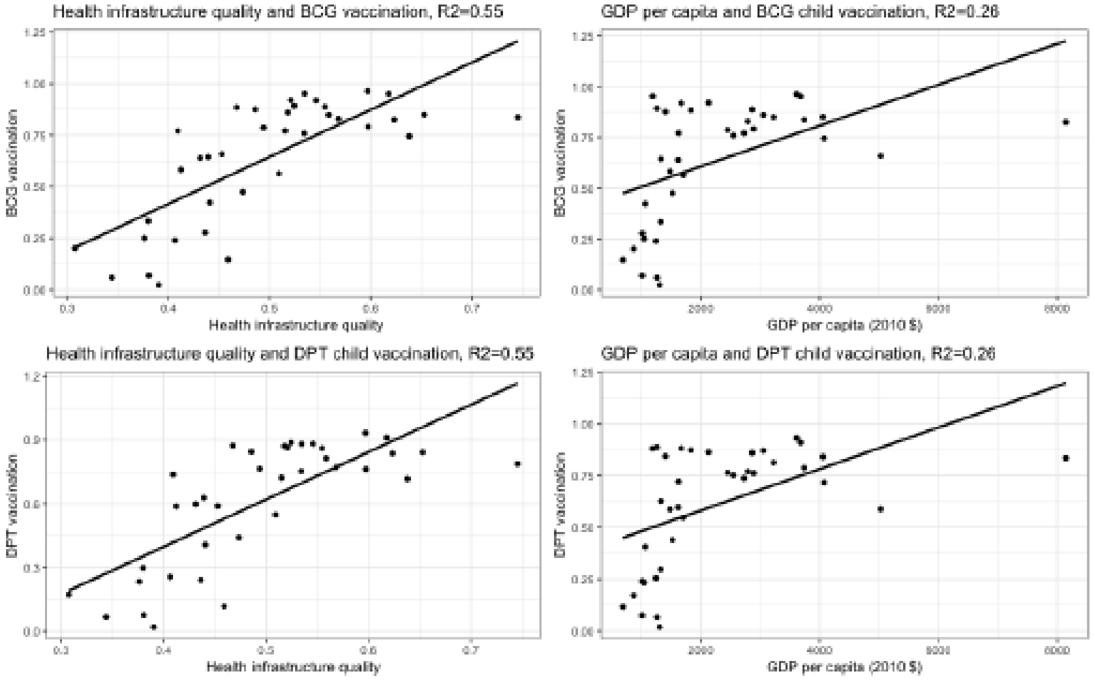

Supporting material for Panel on Maternal, neonatal and child mortality in Nigeria: saving millions of lives - interventions included, LiST tool, projection methods

Overcoming maternal, neonatal and child deaths in Nigeria: investment required to achieve SDGs 3.1, 3.2 and 3.3

Overview

The Sustainable Development Goals 3.1 and 3.2 call for a significant reduction in maternal mortality and an end of preventable neonatal and child deaths under five years old. The global community including Nigeria have committed to achieving these and the other SDGs by 2030, however, maternal and neonatal conditions remain the largest cause of death, disease and disability for the Nigerian population and current levels of neonatal, child and maternal mortality are well above the targets (Table 1). Overcoming this burden will have enormous impacts on the Nigerian population but requires a concerted policy approach and likely substantial health system investment. There has been limited public attention devoted to the level of investment required to meet these ambitious goals or the most effective or efficient package of interventions to achieve them. Using the best available data, we modelled three different policy scenarios (baseline, moderate improvement and universal coverage), to identify the health impacts of different packages of interventions and identify the level of investment required under each.

Method

We used the Lives Saved Tool (LiST) to model the health and cost impacts of the three scenarios across the Nigerian population between 2020 and 2030.

The Lives Saved Tool

LiST is a module within the Spectrum software package which was developed by a combination of UN agencies to project the impact of national demographic changes and government policy decisions on health system outcomes. The tool was initially developed based on the 'Child Survival, Neonatal Survival and Undernutrition' series in *The Lancet* but is regularly updated with new population and health data as they become available. It is linked to modules to estimate the impact of family-planning and AIDS interventions and models the impact of changing levels of coverage of included interventions on the numbers of neonatal, child and maternal deaths across the country.

Interventions included

All interventions with data in the tool were included in our projections. Effectiveness estimates were derived from the literature (http://www.avenirhealth.org/software-spectrum.php)and current coverage levels have been estimated from representative surveys or modelled estimates.

Cost data

Costs were calculated using an ingredients approach that estimated the amount and type of drugs, supplies and personnel time that would be required to deliver each intervention. These costs were assigned to each intervention based on WHO CHOICE and UNICEF drug supply cost data for Nigeria. The staff types and salaries used to calculate staff costs are outlined in Table 3. Salaries were assumed to increase by 3.1% annually and the cost of all consumables included allowances for wastage, inefficiencies and logistics costs.

Scenarios Modelled

Three scenarios were modelled: (1) a baseline scenario that assumed no increase in coverage of interventions over time; (2) a moderate scenario that assumed that planned increases under the NSHDPII were achieved and improvements continued until 2030; and (3) a universal health coverage scenario modelled as 90% coverage of all interventions. The impacts of all scenarios were modelled out to 2030, with 2020 used as the base year for the analysis. In addition, two further analyses were carried out to demonstrate the potential lives that could be saved in 2021 if (1) 90% coverage of these selected interventions was achieved immediately in Nigeria; and, (2) if coverage patterns experienced by the highest quintile of income earners was replicated across the population.

Results

Table 3 presents the overall mortality rate outcomes under each scenario. Under the baseline scenario whereby current coverage patterns are maintained, maternal mortality worsens by 2030 as a result of demographic changes (primarily a projected decrease in the fertility rate), while neonatal and under 5 mortality remain largely stable. Improvements in all outcomes are evident under the moderate and UHC scenarios, with greater decreases under the UHC scenario as expected. Under all scenarios modelled, Nigeria remains off-track from achieving SDG 3.1 and 3.2. Nonetheless, scaling-up the package of interventions under both scenarios results in substantial lives saved over the next decade across Nigeria. Figure 1 shows the number of maternal, neonatal and child deaths under each scenario, demonstrating the potential lives saved by these different policy packages. In total, over 309,000 maternal, 967,000 neonatal and over 2.61 million child deaths could be averted under the UHC scenario relative to baseline. For the Moderate scenario, over 160,500 maternal, 664,000 neonatal and almost 806,000 child deaths could be averted relative to baseline.

Table 4 and 5 show the level of additional resourcing required to achieve these results relative to baseline in terms of money and additional staff required respectively. While the UHC scenario is substantially more expensive than the other options, the per capita cost (using the population

projections under each scenario) are relatively modest. Figures 2 and 3 demonstrate the potential impacts of the different interventions on the numbers of deaths and stillbirths experienced showing that better management of labour could result in a large proportion of the projected benefits.

Table S6 - Maternal, neonatal and child mortality outcomes and targets for Nigeria

**Indicator**	**Current level**	**SDG Target for Nigeria**
Maternal mortality (per 100,000 live births)	917	Less than 140
Neonatal mortality (per 1000 live births)	36	12
Under-5 mortality (per 1000 live births)	117	25

Table S7 - Staff cadre and salaries used in projections

Staff cadre	Annual salary (NGN)
Generalists/primary care doctors	2,806,201.89
Ob\Gyns	4,212,397.36
Paediatricians	4,212,397.36
Other specialist doctors	4,212,397.36
Clinical officers/surgical technicians	1,733,135.04
Nurses	1,733,135.04
Midwives	1,733,135.04
Assistant nurses and midwives	1,312,760.18
Nursing aides	1,312,760.18
Laboratory technicians/assistants	1,733,135.04
Pharmaceutical technicians/assistants	1,733,135.04
Radiographers/X-ray technicians	1,733,135.04
Emergency medical technicians	1,733,135.04
Community health workers	1,006,754.64

Table S8 - Summary of mortality rates under each scenario

Scenario	2020	2021	2022	2023	2024	2025	2026	2027	2028	2029	2030
**Baseline**											
**Maternal mortality ratio (deaths per 100,000 live births)**	917	919.63	922.38	925.1	927.78	930.41	932.99	935.51	937.97	940.37	942.7
**Neonatal mortality rate (deaths per 1,000 live births)**	35.86	35.86	35.86	35.86	35.85	35.85	35.85	35.85	35.85	35.85	35.85
**Under five mortality rate (deaths per 1,000 live births)**	117.19	116.19	116.19	116.25	116.26	116.27	116.24	116.21	116.21	116.18	116.3
**Moderate Improvement**											
**Maternal mortality ratio (deaths per 100,000 live births)**	917	902.49	888.41	874.39	860.41	846.5	760.59	746.09	731.78	717.66	703.75
**Neonatal mortality rate (deaths per 1,000 live births)**	35.86	34.92	34.01	33.11	32.24	31.39	29.08	28.24	27.42	26.62	25.84
**Under five mortality rate (deaths per 1,000 live births)**	117.19	114.22	112.26	110.39	108.5	106.63	103.38	101.51	99.69	97.89	97.42
**UHC**											
**Maternal mortality ratio (deaths per 100,000 live births)**	917	863.19	813.48	695.24	651.41	611.27	574.62	541.28	511.1	483.94	459.66
**Neonatal mortality rate (deaths per 1,000 live births)**	35.87	34.25	32.72	30	28.53	27.14	25.81	24.54	23.33	22.19	21.09
**Under five mortality rate (deaths per 1,000 live births)**	117.19	107.05	99.26	91.32	84.97	79.17	73.89	69.08	64.73	60.78	57.22

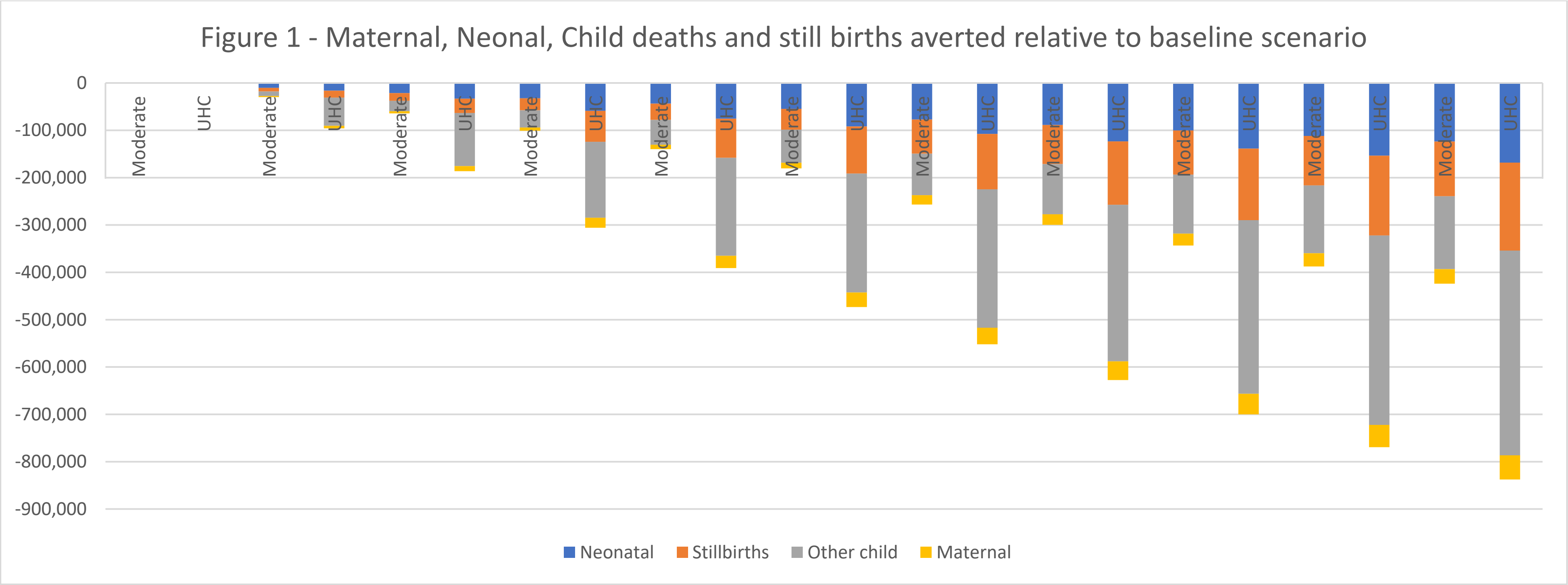

Figure 1 - Maternal, Neonal, Child deaths and still births averted relative to baseline scenario
0
-100,000
-200,000
-300,000
-400,000
-500,000
-600,000
-700,000
-800,000
-900,000
Moderate
UHC
Neonatal
Stillbirths
Other child
Maternal

Figure S4

	2020	2021	2022	2023	2024	2025	2026	2027	2028	2029	2030
**UHC**											
Additional total cost relative to baseline (Million NGN)	0	108,100	213,617	338,186	442,089	543,370	642,600	739,956	835,348	928,777	1,023,479
Additional total cost relative to baseline (million USD)	0	278	549	869	1,136	1,397	1,652	1,902	2,147	2,388	2,631
Additional total cost relative to baseline (USD per capita)	0	1.35	2.60	4.02	5.14	6.18	7.15	8.06	8.91	9.71	10.49
**Moderate**											
Additional total cost relative to baseline (NGN)	0	18,201	35,999	54,038	72,378	90,992	131,034	151,996	173,485	195,502	181,259
Additional total cost relative to baseline (million USD)	0	47	93	139	186	234	337	391	446	503	467
Additional total cost relative to baseline (USD per capita)	0	0.23	0.44	0.64	0.84	1.04	1.46	1.66	1.85	2.04	1.86

Table S9 - Additional cost relative to baseline for scale up scenarios

Table S10 - Additional FTE required under UHC scenario relative to baseline

Additional FTE required under UHC relative to baseline scenario	2020	2021	2022	2023	2024	2025	2026	2027	2028	2029	2030
Generalists/primary care doctors	0	206	371	498	590	649	681	689	680	656	622
Ob\Gyns	0	147	286	697	854	1,007	1,156	1,300	1,439	1,572	1,699
Other specialist doctors	0	12	25	43	56	69	82	95	108	121	134
Nurses	0	1,994	3,809	5,478	6,996	8,367	9,629	10,801	11,894	12,922	13,987
Midwives	0	2,388	4,685	8,877	11,272	13,626	15,931	18,182	20,368	22,481	24,513
Assistant nurses and midwives	0	597	1,191	2,295	2,936	3,569	4,196	4,815	5,423	6,020	6,601
Laboratory technicians/assistants	0	12	24	49	62	74	86	98	110	121	132
Community health workers	0	1,998	3,915	5,761	7,549	9,274	10,962	12,624	14,266	15,893	17,511
Other	0	9	17	77	90	102	114	125	136	147	157

Figure S5 - Potentially preventable deaths in 2021 in Nigeria if 90% coverage was achieved immediately by intervention

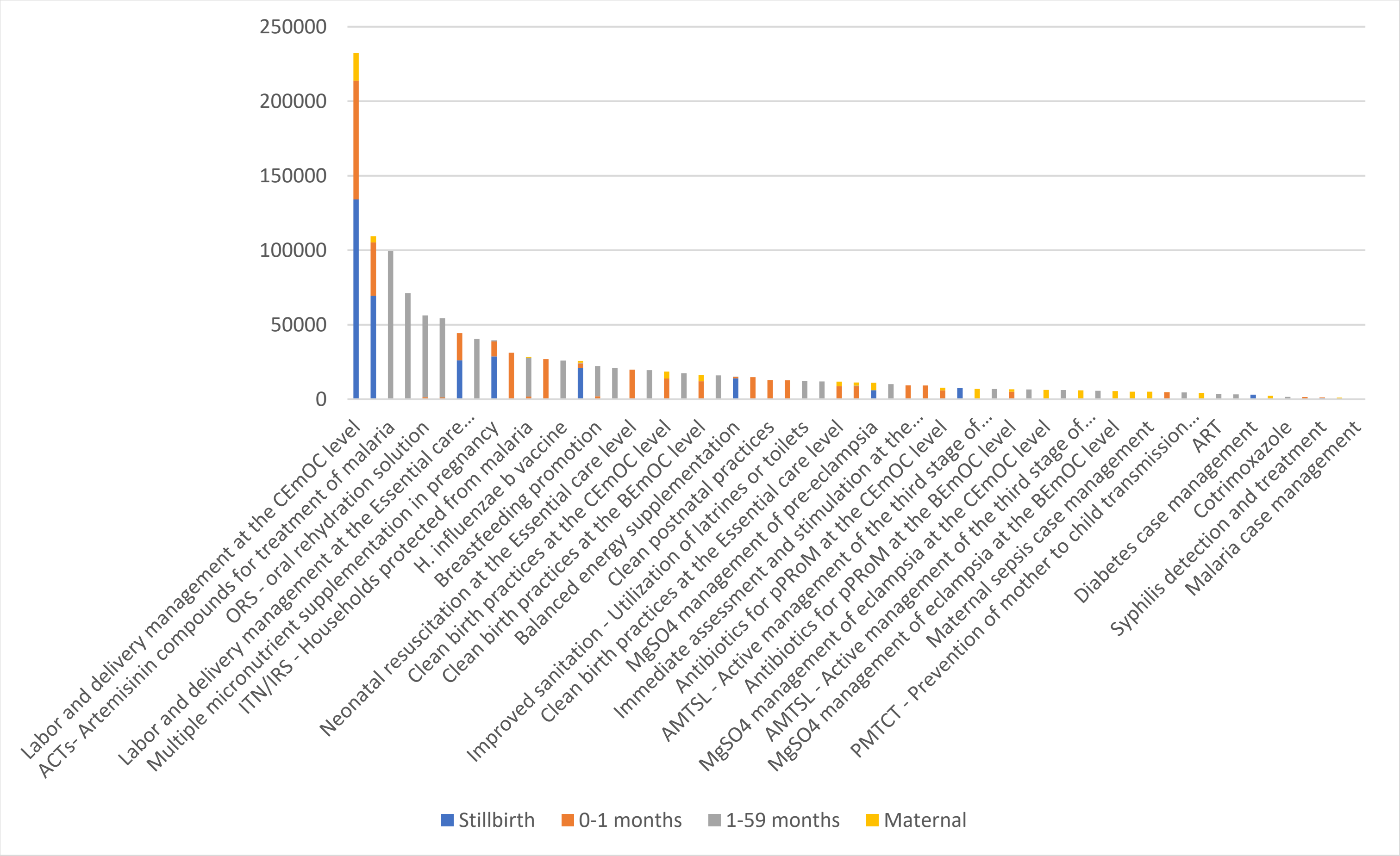

Figure S6 - Potentially preventable deaths in 2021 in Nigeria if coverage of top 20% was achieved across the population – closing the equity gap

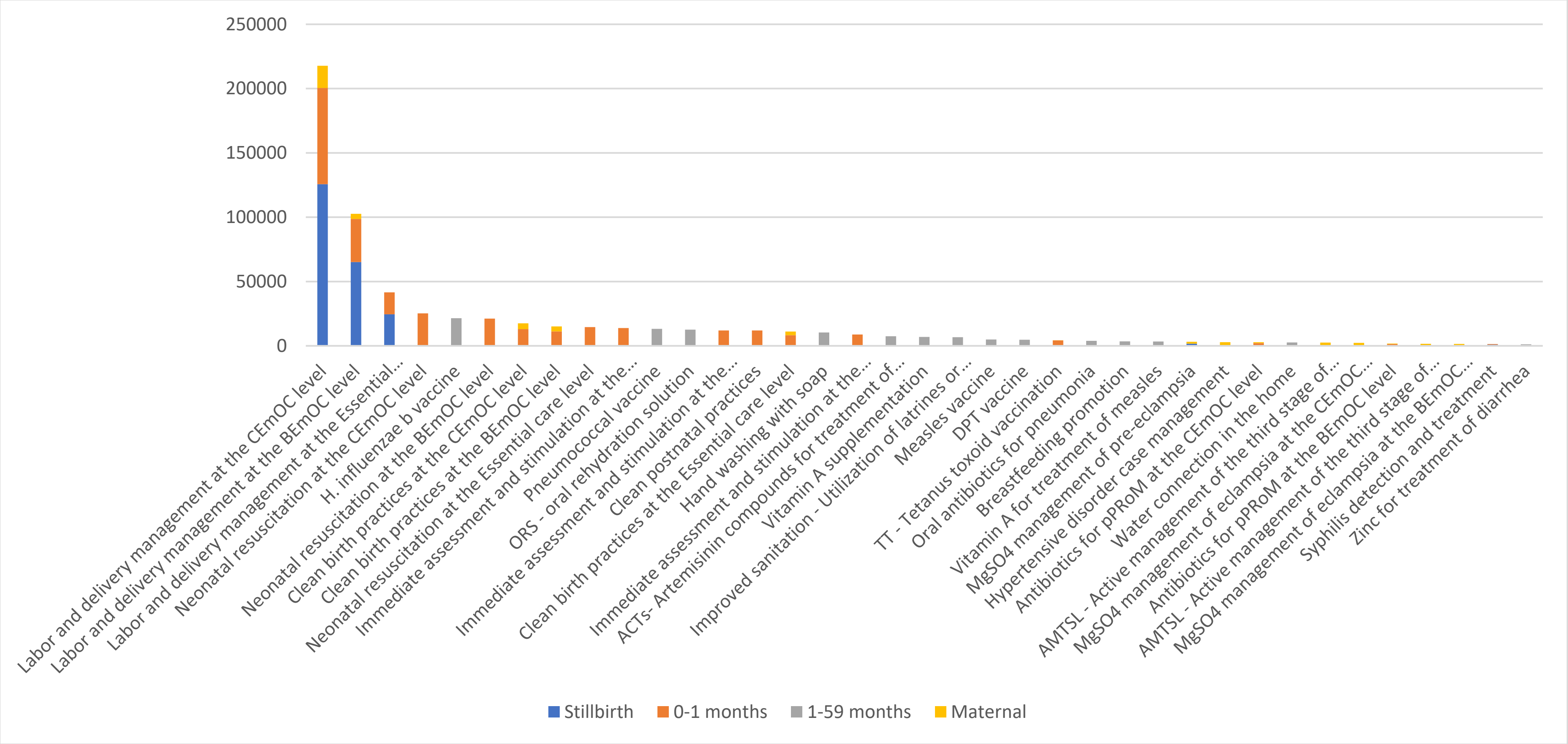

Details on *LiST* Effect sizes

Effect Sizes related to Maternal Causes of Death that were used

Cause of Death	Intervention	Effect
Hypertensive diseases	Comprehensive Emergency Obstetric Care	.95
	Calcium supplementation	.20
	Comprehensive Emergency Obstetric Care	.96
Infections	Antibiotics for pPRoM	.26 (.1)
	Clean practices and immediate essential newborn care (home)	.1 (.5)
Postpartum Hemorrhage	Essential care for all women and immediate essential newborn care (facility)	.1 (.5)
	Basic Emergency Obstetric Care	.5

Cause of Death	Intervention	Effect
Obstructed labor	Basic Emergency Obstetric Care	.08
	Comprehensive Emergency Obstetric Care	.99
Ectopic pregnancy	Ectopic pregnancy case management (BEmOC level)	.3
	Ectopic pregnancy case management (CEmOC level)	.9
Malaria	IPTp	.4
Other indirect causes	Tetanus toxoid immunization	.98 (.005)
Antepartum Hemorrhage	Basic Emergency Obstetric Care	.2

	Comprehensive Emergency Obstetric Care	.7
	Active management of the third state of labor	.27
	Basic Emergency Obstetric Care	.65

	Comprehensive Emergency Obstetric Care	.8
Note: All affected fractions are equal to 1 unless otherwise stated. All numbers in parentheses are the relevant affected fractions		

Effect Sizes related to Neonatal Causes of Death

Cause of Death	Intervention	Effect
Diarrhea	ORS	0.93
Sepsis Pneumonia	Syphilis detection and treatment	0.025
	Antibiotics for pPRoM	0.08
	Essential care for all women and immediate essential newborn care	0.25
	Basic emergency obstetric care	0.25
	Comprehensive emergency obstetric care	0.25

Cause of Death	Intervention	Effect
Prematurity	Antenatal corticosteroids for preterm labor	0.53
	Antibiotics for pPRoM	0.12
	Essential care for all women and immediate essential newborn care	0.10
	Basic emergency obstetric care	0.10
	Comprehensive emergency obstetric care	0.10
	Neonatal resuscitation (institutional)	0.10

	Clean practices and immediate essential newborn care (home)	0.20
	Preventive postnatal care (healthy practices & illness detection)	0.31
	Oral antibiotic case management of severe infection	0.42
	Injectable antibiotic case management of severe infection	0.68
	Case management of severe infection with full supportive care	0.83
Asphyxia	Essential care for all women and immediate essential newborn care	0.25
	Basic emergency obstetric care	0.40
	Comprehensive emergency obstetric care	0.80
	Neonatal resuscitation (institutional)	0.30
	Neonatal resuscitation (home)	0.20
	Case management of severe infection with full supportive care	0.05

	Neonatal resuscitation (home)	0.05
	Preventive postnatal care (healthy practices & illness detection)	0.35
	Kangaroo mother care	0.51
	Case management of severe illness with full supportive care	0.28
Tetanus	Tetanus toxoid	0.94
	Essential care for all women and immediate essential newborn care	0.36
	Basic emergency obstetric care	0.36
	Comprehensive emergency obstetric care	0.36
	Clean practices and immediate essential newborn care (home)	0.30
Congenital anomalies	Periconceptual Folic Acid	0.35
Other	Case management of serious neonatal illness	0.10

Table S11 Effect Sizes related to Childhood Causes of Death that were used

Cause of Death	Intervention	1-6 months		6-12 months		12-23 months		24-59 months	
		Effect	AF	Effect	AF	Effect	AF	Effect	AF
Diarrhea	Use of improved water source within 30 minutes	.17	1	.17	1	.17	1	.17	1
	Use of water connection in the home	.69	1	.69	1	.69	1	.69	1
	Improved excreta disposal (latrine/toilet)	.36	1	.36	1	.36	1	.36	1
	Hand washing with soap	.48	1	.48	1	.48	1	.48	1
	Hygienic disposal of children's stool	.20	1	.20	1	.20	1	.20	1
	Vitamin A for prevention	0	1	0.31	1	0.31	1	0.31	1
	Zinc for prevention	0	1	0.13	1	0.13	1	0.13	1
	Rotavirus vaccine	.74	0.39	0.74	0.39	0.74	0.39	0.74	0.39
	ORS	0.93	0.95	0.93	0.95	0.93	0.95	0.93	0.95
	Antibiotics for dysentery	0.99	0.05	0.99	0.05	0.99	0.05	0.99	0.05
	Zinc for treatment	0.23	1	0.23	1	0.23	1	0.23	1
Pneumonia	Zinc for prevention	0	1	0.15	1	0.15	1	0.15	1
	Hib vaccine	0.18	1	0.18	1	0.18	1	0	1
	Pneumococcal vaccine	0.24	1	0.24	1	0.24	1	0.24	1
	DPT vaccination	0.1	1	0.1	1	0.1	1	0.1	1

	Case management of pneumonia (oral antibiotics)	0.7	1	0.7	1	0.7	1	0.7	1
Measles	Measles vaccine	.85	1	0.85	1	0.85	1	0.85	1
	Vitamin A for measles treatment	0.62	1	0.62	1	0.62	1	0.62	1
Malaria	Insecticide treated materials/indoor residual spraying	0.55	1	0.55	1	0.55	1	0.55	1
	Antimalarials	0.84	1	0.84	1	0.84	1	0.84	1

References

1. Angell B, Dodd R, Palagyi A, et al. Primary health care financing interventions: a systematic review and stakeholder-driven research agenda for the Asia-Pacific region. *BMJ global health* 2019; **4**(Suppl 8): e001481.
2. Balabanova Y, Gilsdorf A, Buda S, et al. Communicable diseases prioritized for surveillance and epidemiological research: results of a standardized prioritization procedure in Germany, 2011. *PloS one* 2011; **6**(10): e25691.
3. Institute of Health Metrics and Evaluation. Global Burden of Disease Study 2019. 2020.
4. Vos T, Lim SS, Abbafati C, et al. Global burden of 369 diseases and injuries in 204 countries and territories, 1990–2019: a systematic analysis for the Global Burden of Disease Study 2019. *The Lancet* 2020; **396**(10258): 1204-22.
5. Collaborators G, Ärnlöv J. Global burden of 87 risk factors in 204 countries and territories, 1990–2019: a systematic analysis for the Global Burden of Disease Study 2019. *The Lancet* 2020; **396**(10258): 1223-49.
6. Office of the Senior Special Assistant to the President on MDGs (OSSAP). Nigeria MDG Information System (http://nmis.mdgs.gov.ng/. Accessed: 2015-09-30, 2012.
7. Archibong B, Modi V, Sherpa S. Geography of Infrastructure Functionality at Schools in Nigeria: Evidence From Spatial Data Analysis Across Local Government Areas. *Papers in Applied Geography* 2015; **1**(2): 176-83.
8. Makinde OA, Sule A, Ayankogbe O, Boone D. Distribution of health facilities in Nigeria: Implications and options for Universal Health Coverage. *The International Journal of Health Planning and Management* 2018; **33**(4): e1179-e92.
9. Okpani AI, Abimbola S. Operationalizing universal health coverage in Nigeria through social health insurance. *Niger Med J* 2015; **56**(5): 305-10.

Web Appendix 5. Additional figures

Figure S7. Nigeria population structure 1950 and 2020. Data from United Nations, Department of Economic and Social Affairs, Population Division (2019). World Population Prospects 2019, Online Edition. Rev. 1.

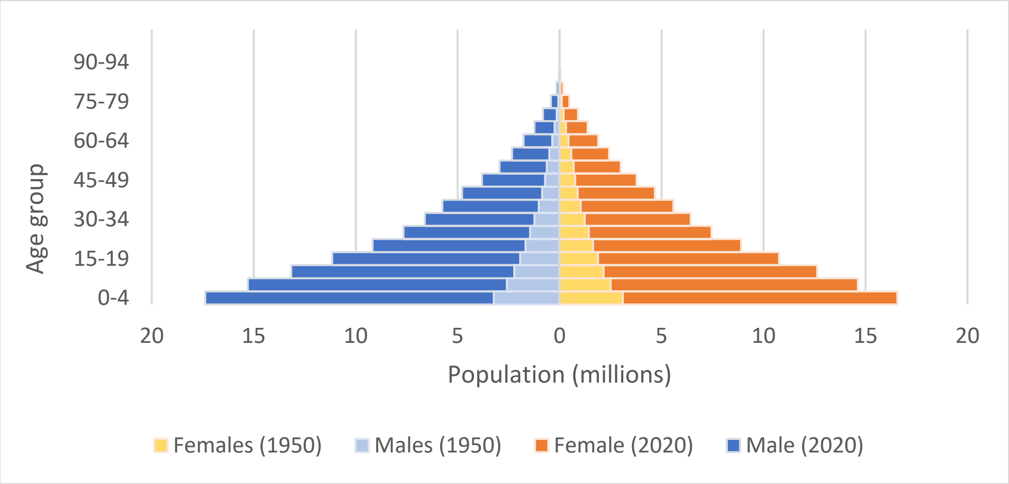

Figure S8: Percentage of women age 15-49 with a secondary education or higher, Nigeria DHS 1999-2018

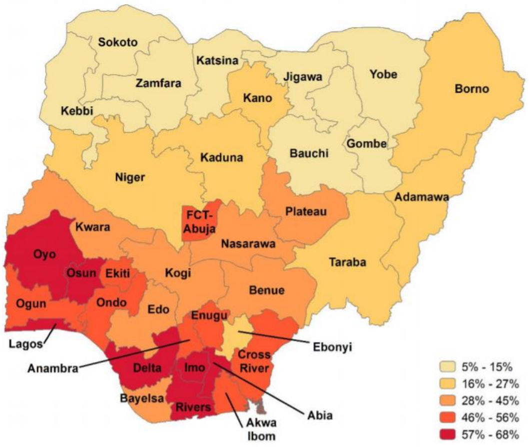

Source: National Population Council, 2019

Figure S9: Life expectancy at birth in Nigeria 1950-2020. Data from United Nations, Department of Economic and Social Affairs, Population Division (2019). World Population Prospects 2019, Online Edition. Rev. 1.

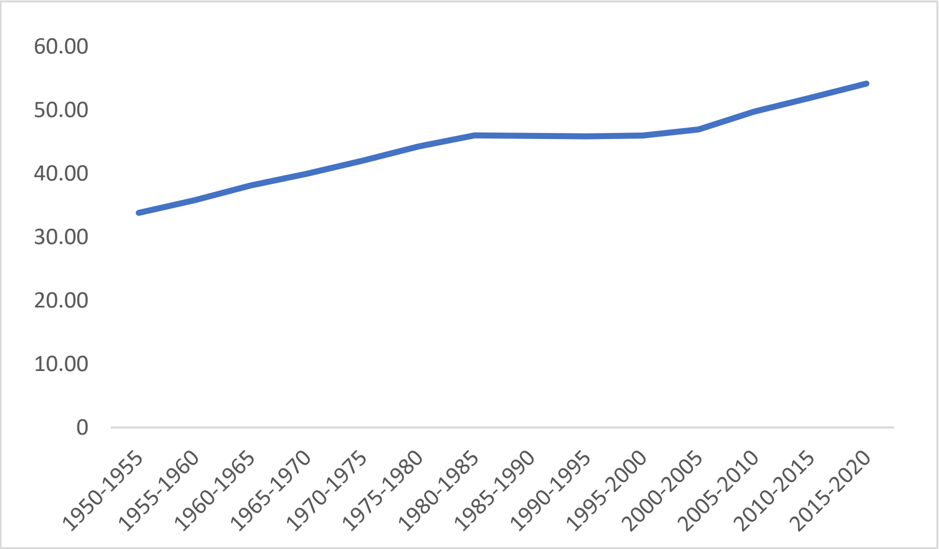

Figure S11. Trend in percent of total (a) DALYs and (b) deaths attributed to cardiometabolic risk factors in Nigeria, 1990-2019

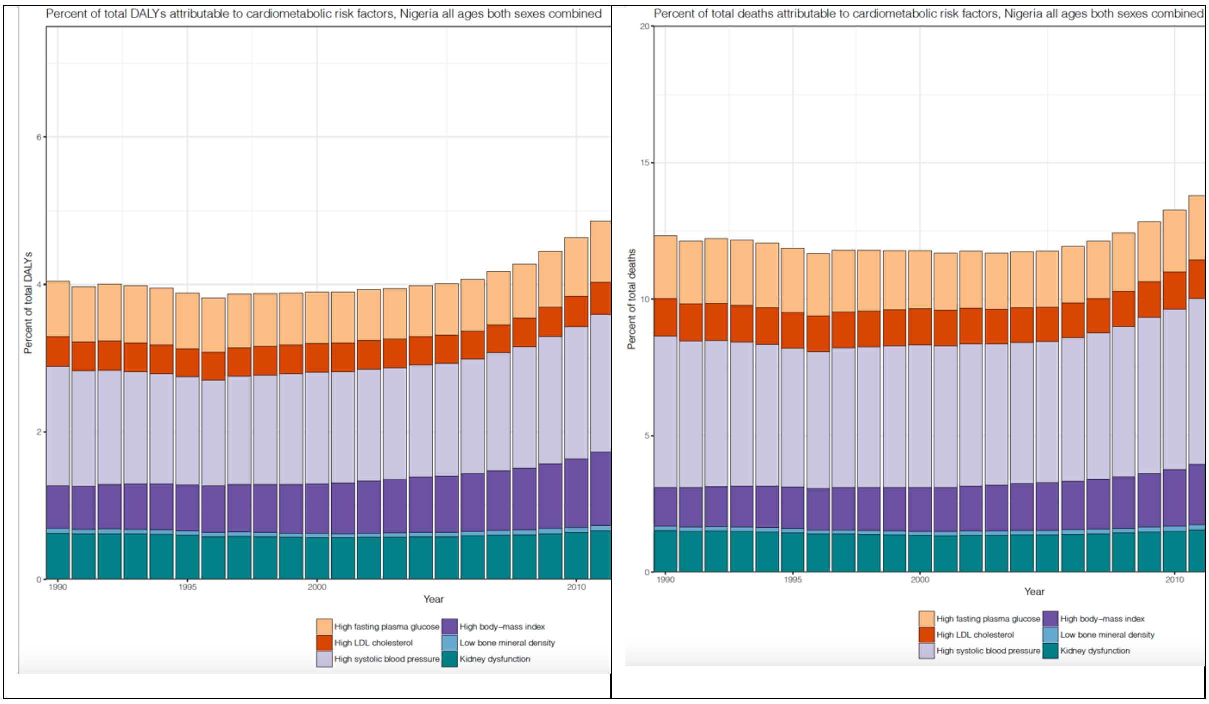

Source: IHME

Figure S12. Share of government spending on health and education in Nigeria, 1981-2018 (Source: Nigeria NBS)

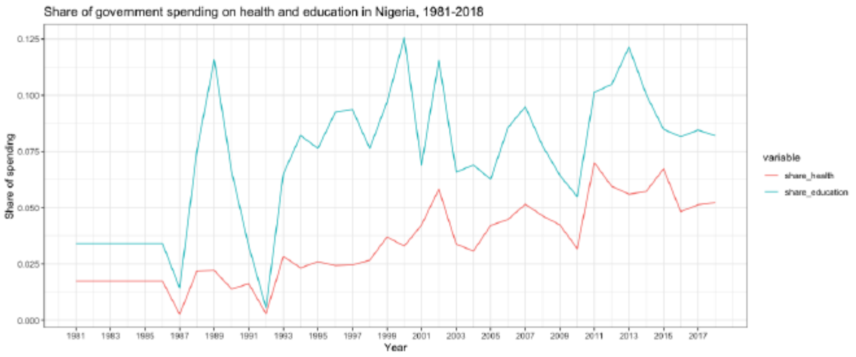

Figure S13. Share of spending in total health expenditures in Nigeria and SSA, 1995-2014 (Source: IHME)

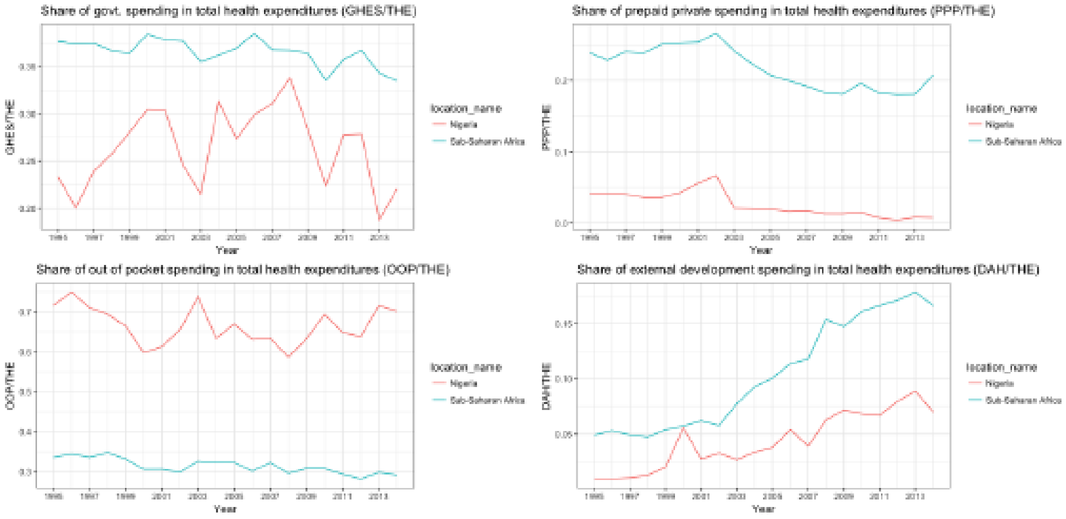

Supplement: Supplementary appendix [file mmc1.pdf]
